# Cytosolic nucleic acid sensing as driver of critical illness: mechanisms and advances in therapy

**DOI:** 10.1038/s41392-025-02174-2

**Published:** 2025-03-19

**Authors:** Zhaorong Chen, Rayk Behrendt, Lennart Wild, Martin Schlee, Christian Bode

**Affiliations:** 1 Department of Anesthesiology and Intensive Care Medicine, University Hospital Bonn, 53127 Bonn, Germany; 2 Institute of Clinical Chemistry and Clinical Pharmacology, University Hospital Bonn, 53127 Bonn, Germany

**Keywords:** Inflammation, Trauma, Molecular medicine, Infectious diseases

## Abstract

Nucleic acids from both self- and non-self-sources act as vital danger signals that trigger immune responses. Critical illnesses such as acute respiratory distress syndrome, sepsis, trauma and ischemia lead to the aberrant cytosolic accumulation and massive release of nucleic acids that are detected by antiviral innate immune receptors in the endosome or cytosol. Activation of receptors for deoxyribonucleic acids and ribonucleic acids triggers inflammation, a major contributor to morbidity and mortality in critically ill patients. In the past decade, there has been growing recognition of the therapeutic potential of targeting nucleic acid sensing in critical care. This review summarizes current knowledge of nucleic acid sensing in acute respiratory distress syndrome, sepsis, trauma and ischemia. Given the extensive research on nucleic acid sensing in common pathological conditions like cancer, autoimmune disorders, metabolic disorders and aging, we provide a comprehensive summary of nucleic acid sensing beyond critical illness to offer insights that may inform its role in critical conditions. Additionally, we discuss potential therapeutic strategies that specifically target nucleic acid sensing. By examining nucleic acid sources, sensor activation and function, as well as the impact of regulating these pathways across various acute diseases, we highlight the driving role of nucleic acid sensing in critical illness.

## Introduction

Misplaced nucleic acid (NA) in cytoplasm and circulation originates from host cells and exogenous sources such as pathogens, serving as damage-associated molecular patterns (DAMPs) and pathogen-associated molecular patterns (PAMPs) to initiate danger signals via NA sensors. The discovery of NA-sensing receptors dates back to the last century. Deoxyribonucleic acid (DNA) sensing pathways include toll-like receptor (TLR) 9 signaling that increases the release of type I interferon (IFN) and proinflammatory cytokines, the cyclic GMP-AMP synthase (cGAS)-stimulator of IFN genes (STING) pathway that predominantly initiates type I IFN response, and the absent in melanoma (AIM) 2 inflammasome that produces interleukin-1β (IL-1β) and IL-18 and triggers pyroptosis.^1^ Conversely, ribonucleic acid (RNA) sensing pathways comprise TLR3-TIR (Toll/interleukin-1 receptor) domain-containing adaptor protein inducing interferon beta (TRIF), TLR7/8-myeloid differentiation primary response protein (MyD88), and retinoic acid-inducible gene I (RIG-I)/melanoma differentiation-associated protein 5 (MDA5)-mitochondrial antiviral signaling (MAVS) signaling that induce the expression of type I and III IFNs and proinflammatory cytokines.^2^ Immunity mediated by NA not only defends against pathogen but also triggers sterile inflammation and drives the progression of non-infectious diseases.

The contribution from NA sensing to DAMP- and PAMP-driven host responses has been widely understood in the past decade, especially in inflammatory diseases, cancer, autoimmune diseases, metabolic disorder and aging.^3–6^ In recent years, NA sensing is gradually recognized as the driver of critical conditions. Growing numbers of studies focus on the role of NA sensing in critical care medicine, providing potential and promising targets for disease management. Here, we review present knowledge of NA sensing in acute respiratory distress syndrome (ARDS), sepsis, trauma and ischemia. We also briefly summarize current understanding of NA sensing in non-critical conditions, including cancer, autoimmune disease, metabolic disorder and aging. Our discussion on the driving role of NA and downstream signaling in critical illness sheds light on the future exploration of NA sensing-based therapeutic strategy from bench to bedside.

## Discovery of nucleic acid sensors

**Fig. 1:**
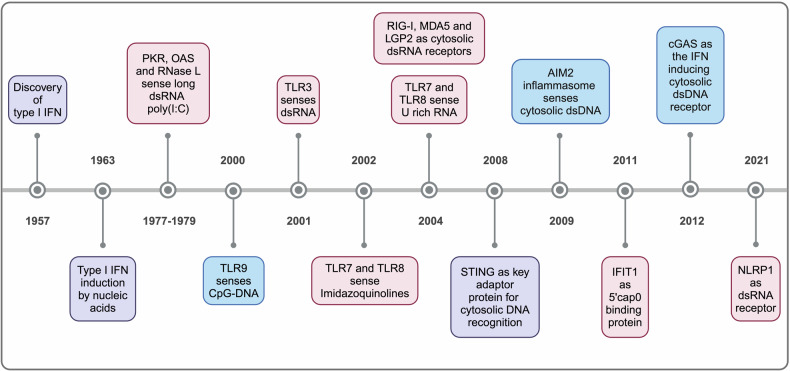
Timeline diagram of milestones in nucleic acid sensing research. Significant discoveries of fundamental DNA sensors (blue), RNA sensors (red) and key signaling proteins (purple) are demonstrated in chronological order. Figure created with BioRender.com

In 1957, a factor named IFN that interferes with viral replication was discovered (Fig. 1).^7^ Several years later, Rotem, Cox and Isaacs found that NAs are able to induce the secretion of type I IFN in NA-treated chicken and mouse cells in 1963.^8^ The immunostimulatory NAs suggested the presence of NA sensing.^8–11^ However, it was not until 2000 that the Akira lab identified TLR9 as the first NA sensor, recognizing unmethylated cytosine-phosphate-guanosine (CpG) DNA (Fig. 1).^12^ Since CpG oligodeoxynucleotides (ODNs) were applied extracellularly in the study, the basis of cytosolic DNA sensing remained unclear. Amid debates on TLR9-independent IFN inducing mechanisms, the key adaptor protein for cytosolic DNA recognition, STING, was discovered in 2008.^13–16^ One year later, the pyrin and hematopoietic interferon-inducible nuclear domain protein AIM2 was reported as a novel intracellular DNA detector and inflammasome activator, now regarded as a non-redundant inflammasome activating DNA sensor.^17–20^ While the AIM2 inflammasome triggers caspase-1 activation and the release of IL-1β and IL-18, it does not account for the IFN production. This indicated the presence of at least one additional cytosolic DNA receptor. In 2012, the lab of Zhijian James Chen made a significant breakthrough by discovering the cytoplasmic DNA sensor cGAS.^21,22^ They found that the endogenous second messenger 2´3´-cyclic-GMP-AMP (cGAMP) activates the adaptor protein STING, with cGAS generating cGAMP upon DNA recognition.^21,22^ The discovery of the cGAS-STING pathway is notable for its fundamental role in cytosolic DNA recognition and its relevance to various diseases, highlighting its potential as a therapeutic target.

The field of RNA sensors developed alongside that of DNA sensors. While RNA molecules were the first NA found to be immunostimulatory, the earliest identified RNA sensors, 2′-5′ oligoadenylate synthase (OAS) and protein kinase R (PKR), primarily degrade RNA and restrict messenger RNA (mRNA) translation, respectively, without inducing an IFN response.^23–27^ It took years before TLR3 was identified in 2001 as a crucial receptor that induces an IFN response and nuclear factor kappa-light-chain-enhancer of activated B cells (NF-κB) activation upon recognition of polyinosinic:polycytidylic acid [poly(I:C)], a synthetic RNA regarded as a surrogate of natural occurring viral double-stranded RNA (dsRNA),^28^ while TRIF was later shown to be the adaptor protein for TLR3.^29–32^ Advances in RNA synthesis technology enabled the significant identification of single-stranded RNA (ssRNA) as a ligand for TLR7 and TLR8 in 2004, with MyD88 acting as an adaptor in TLR signaling.^33–37^ In the same year, researchers identified the cytosolic helicases RIG-I, MDA5 and laboratory of genetics and physiology 2 (LGP2) as new dsRNA receptors.^38–45^ While both RIG-I and MDA5 signal via MAVS,^46–49^ LGP2 cannot interact with MAVS but influences the activity of RIG-I and MDA5. The function and mechanism of LGP2 remain controversial. Further progress on RNA sensors was made when interferon-induced protein with tetratricopeptide repeats 1 (IFIT1) and the NLR family pyrin domain containing (NLRP) 1 inflammasome were identified as novel dsRNA receptors in 2011 and 2021, respectively.^50–52^

## Nucleic acid sensors

### DNA sensors

#### TLR9

TLR9 is a DNA sensor that responds to deoxyribonuclease (DNase) II cleavage products of microbial- and other pathogen-derived DNA containing unmethylated CpG dinucleotides.^1,53^ In humans, TLR9 is predominantly expressed in B cells and plasmacytoid dendritic cells (pDCs),^54,55^ while under some circumstances neutrophils, platelets and erythrocytes can also express TLR9.^56–59^ In mice, TLR9 is expressed in dendritic cells, macrophages and B lymphocytes.^1^ As an intracellular member of the TLR family, TLR9 is composed of a leucine-rich repeat domain that recognizes DNA and a Toll/interleukin-1 receptor domain that initiates downstream signaling. In an inactive state, TLR9 is retained in the endoplasmic reticulum,^60^ from where it translocates to endolysosomes through the Golgi complex.^61^ Upon endolysosomal acidification, the leucine-rich repeat region is cleaved, converting inactive TLR9 into a functional form that binds to CpG-DNA.^62,63^ The Toll/interleukin-1 receptor domain then recruits MyD88 and activates MyD88-dependent pathways.^64^ Of note, TLR9 also detects self-DNA especially mitochondrial DNA (mtDNA), as mitochondria are bacteria-derived evolutionary endosymbionts and mtDNA shares many similarities with bacterial DNA.^65,66^

**Fig. 2:**
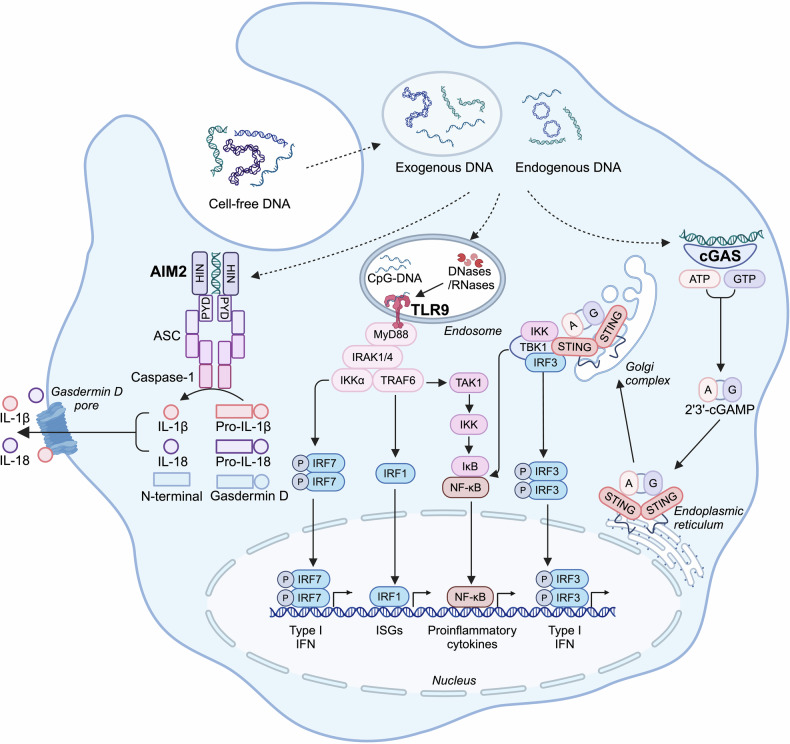
DNA sensing pathways. Distinct sensing pathways are activated by DNA derived from exogenous and endogenous sources. TLR9 detects CpG-DNA and recruits MyD88 to induce IRF7-mediated type I IFN production, IRF1-mediated ISGs expression and NF-κB-mediated pro-inflammatory cytokine production. Upon recognition of dsDNA, cGAS catalyzes cGAMP synthesis to activate STING that initiates IRF3-mediated type I IFN response and NF-κB-mediated inflammatory response. Furthermore, dsDNA-bound AIM2 drives inflammasome assembly with ASC and caspase-1, triggering the release of IL-1β and IL-18 as well as pyroptosis. Figure created with BioRender.com. ATP adenosine triphosphate, GTP guanosine-5’-triphosphate, HIN hematopoietic interferon-inducible nuclear, ISGs interferon-stimulated genes, IκB inhibitor of nuclear factor kappa B, PYD pyrin domain

As shown in Fig. 2, a key function mediated by TLR9-MyD88 signaling is the type I IFN response.^67^ On one hand, the interaction between TLR9 and MyD88 induces a complex formed with tumor necrosis factor receptor–associated factor (TRAF) 6, IL-1 receptor-associated kinase (IRAK) 1 and IRAK4, which activates interferon regulatory factor (IRF) 7 to translocate into nucleus and induce type I IFN production.^68–70^ On the other hand, MyD88 promotes nuclear translocation of IRF1 to induce expression of IFN-stimulated genes.^71,72^ Another event triggered by TLR9-MyD88 signaling is the activation of NF-κB. The recruited TRAF6 activates TGF-β associated kinase 1 (TAK1), which phosphorylates inhibitor of nuclear factor-κB kinase (IKK) complex and leads to NF-κB activation and proinflammatory cytokine release.^73^ In addition, TAK1 results in activation of mitogen-activated protein kinase (MAPK) family and activator protein-1 family.^74^

#### cGAS-STING

The cGAS-STING signaling is the only non-redundant pathway for sensing cytosolic double-stranded DNA (dsDNA) and activating IFN production.^75^ This pathway is active across human and murine cells, including dendritic cells (DCs), macrophages, T cells, endothelial and epithelial cells.^76^ Briefly, mammalian cGAS catalyzes the synthesis of secondary messenger 2´3´-cGAMP upon recognition of DNA.^22^ cGAMP then activates STING that eventually initiates Type I IFN production via IRF3 (Fig. 2).^21,77,78^

The cytoplasmic nucleotidyl transferase cGAS recognizes long B-form DNA via DNA phosphate-sugar backbone in a sequence-independent but length-dependent manner and short Y-form DNA in a sequence dependent manner.^79–81^ The cytosolic DNA detected by cGAS in not limited to pathogen-derived DNA but also includes self-DNA.^82^ DNA-bound cGAS undergoes a structural switch, termed conformation transition, and forms a catalytic pocket to synthesize cGAMP from GTP and ATP.^79,83^ Importantly, cGAS is also present in the nucleus. Here tethering to chromatin prevents it’s activation by genomic DNA while it can still be activated by naked DNA during G1/0-G2 phase.^84,85^ During the G2/M-M phase, phosphorylation of the N-terminus of cGAS prevents its activation by any DNA.^84^

STING, the sensor protein of cGAMP, plays the central role in downstream signaling. When binding to cGAMP, STING undergoes a conformational switch, oligomerization and translocation from endoplasmic reticulum membrane to Golgi, endowing itself the signaling competence.^86–88^ Inducing Type I IFN response is the best-studied function of STING signaling. Functional STING recruits and activates TANK-binding kinase 1 (TBK1), which in turn phosphorylates STING and subsequently phosphorylates the recruited transcription factor IRF3. Phosphorylated IRF3 forms dimers and drives the transcription of Type I IFN encoding genes.^89–91^ Secondly, STING also leads to NF-κB activation and induces transcription of inflammatory cytokines via TBK1.^92^ Two other functions of STING are mediating autophagy^93^ and programmed cell death.^94^

#### AIM2 inflammasome

AIM2 is a cytoplasmic dsDNA sensor from the AIM2-like receptor family that functions via a multimeric protein complex called inflammasome. It is mainly expressed in myeloid-lineage cells, T cells, B cells as well as epithelial and endothelial cells.^95–98^ It has a C-terminal hematopoietic interferon-inducible nuclear domain that directly binds to DNA and an N-terminal pyrin domain that mediates protein-protein interactions.^99^ When detecting cytosolic dsDNA, the positively charged hematopoietic interferon-inducible nuclear domain binds to the sugar-phosphate backbone of B-form DNA in a sequence-independent manner.^100^ As shown in Fig. 2, DNA-bound AIM2 undergoes oligomerization and drives inflammasome assembly, recruiting its adaptor protein, apoptosis-associated speck-like protein containing a caspase recruitment domain (ASC). ASC further cleaves procaspase-1 to active caspase-1 which in turn cleaves pro-IL-1β, pro-IL-18 and gasdermin D (GSDMD).^20,99^ Cleaved GSDMD induces pore formation on the plasma membrane and triggers pyroptosis, while mature IL-1β and IL-18 are released via the GSDMD pore.^99,101^ AIM2 inflammasome-induced cytokine release and pyroptotic cell death are crucial parts of innate immunity responding to a wide range of pathogens.^102–104^ Importantly, AIM2 also detects self-DNA released from nuclei, mitochondria or lysosomes.^105,106^

The crosstalk between AIM2 inflammasome and cGAS-STING signaling has been actively discussed in previous studies. On one hand, STING-induced type I IFN is important for AIM2 inflammasome activation.^107,108^ On the other hand, AIM2 inflammasome negatively regulates the cGAS-STING pathway via inhibiting STING-TKB1 interaction, interfering with type I IFN production and promoting cell death.^109,110^ Moreover, caspase-1 can cleave cGAS and dampen cGAS-STING signaling upon inflammasome activation.^111^ Interestingly, our previous study found that ODN A151 containing human telomeric DNA motif TTAGGG could suppress both cGAS activation and AIM2 inflammasome presumably via competing with DNA.^112,113^ In conclusion, the AIM2 inflammasome contributes directly and indirectly to DNA sensing.

#### Other DNA sensors

Beyond cGAS, other DNA sensors were described to act in the STING-dependent pathway during DNA sensing. Human interferon-γ-inducible protein 16 (IFI16) and DEAD-box helicase 41 (DDX41) were observed to interact with STING upon DNA recognition.^114–116^ IFI16 belongs to AIM2-like receptor family and its murine homolog is IFI204.^114,117^ IFI16 is expressed in human monocytes, lymphocytes, B cells, neutrophils, endothelial and epithelial cells,^98,114,118–120^ while its murine homolog IFI204 is found in mouse myeloid cells, monocyte/macrophage lineage and embryonic fibroblasts.^114,121^ Unlike cGAS and AIM2, IFI16 is a predominantly nuclear protein that oligomerizes inside nucleus upon DNA binding and translocates into cytoplasm where it drives inflammasome assembly.^1,122–124^ On the other hand, DDX41, a DEAD-box type RNA helicase, binds to DNA via its DEAD domain.^125^ It is primarily expressed in dendritic cells, myeloid cells, macrophages and neutrophils.^115,119,126,127^ DDX41 was described to associate with DNA and STING to enhance a type I IFN response and NF-κB.^115,125^

Z-DNA binding protein 1 (ZBP1), also known as DNA-dependent activator of interferon regulatory factors (DAI), is another cytosolic NA sensor expressed in human B cells as well as murine fibroblasts and T cells.^128,129^ It induces TBK1-IRF3-IFN I and NF-κB signaling, facilitates inflammasome activation and triggers PANoptosis.^130^ In addition, ZBP1 was described to cooperate with cGAS to sustain IFN signaling upon mtDNA recognition.^131^ Other DNA binding proteins that were connected to DNA-induced IFN induction include DExH-box helicase (DHX) 9, DHX36 and RNA polymerase III.^132^ Conceptually, these sensors may have an important role in shaping the immune response downstream of a cGAS-induced IFN-mediated feed-forward response.

### RNA sensors

#### TLR3/7/8

**Fig. 3:**
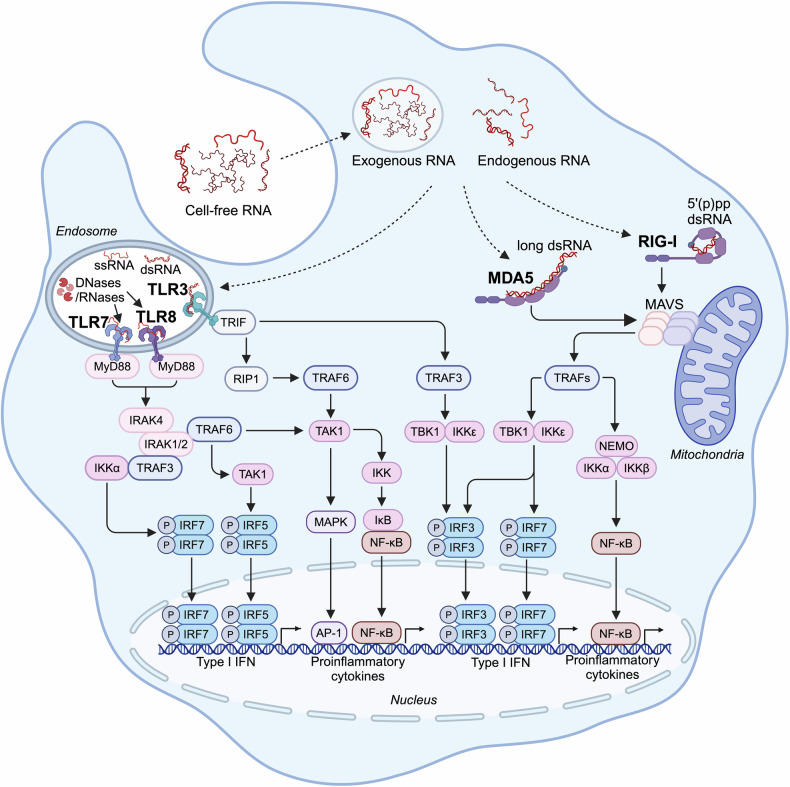
RNA sensing pathways. RNA originating from exogenous and endogenous sources activates downstream RNA sensing pathways. In endosome, TLR3 detects dsRNA and initiates signaling through TRIF, TRAF3 and IRF3 to induce type I IFNs as well as via TRIF, TRAF6, MAPK and NF-κB to the production of proinflammatory cytokines. TLR7 and TLR8 detect ssRNA and induces the expression of type I IFN, chemokines and cytokines via MyD88 signaling and downstream activation of IRF5/7, MAPK and NF-κB. In cytoplasm, RIG-I and MDA5 senses 5’(p)pp dsRNA and long ssRNA, respectively. Activated RIG-I and MDA5 multimerize to enable MAVS to recruit kinases, activating NF-κB and IRF5/7 and inducing transcription of antiviral proteins, chemokines and cytokines. Figure created with BioRender.com. 5’(p)pp dsRNA 5’diphosphorylated or 5’triphosphorylated double-stranded RNA, AP-1 activator protein-1, IκB inhibitor of nuclear factor kappa B, NEMO nuclear factor κB essential modulator, RIP1 receptor-interacting protein 1

The RNA recognizing TLR3, 7 and 8 are localized in the endosome where they recognize long dsRNA (TLR3), short U rich dsRNA (TLR7) or ssRNA (TLR7 and 8) (Fig. 3).^28,133,134^

While TLR7 and 8 are predominantly expressed in immune cells such as pDCs and B cells (TLR7) as well as monocytes and myeloid dendritic cells (TLR8),^55^ TLR3 was also found at the surface of fibroblasts and tumor cells.^135^ Of note, the actual ligands for TLR7 and 8 are RNA cleavage products of ribonucleases (RNases).^136,137^ So far, RNase T2, RNase 2,^138,139^ PLD exonucleases^140^ and RNase 6^141^ have been identified to be involved in RNA cleavage that enables TLR7/8 activation. In contrast to TLR7/8, human TLR3 is activated by long dsRNA (>35 base pair, bp).^142^ Notably, shorter dsRNA such as small interfering RNA (siRNA) can be recognized by mouse TLR3 but not human TLR3, which challenges the usage of mouse models to study the effects of siRNAs and TLR3 agonists.^143,144^ TLR7 in pDCs induces signaling via the adapters MyD88, IRAK1 and IKKα kinase, downstream TRAF6 and MAPKs as well as NF-κB and IRF5/7 activation, leading to the expression of type I IFN, chemokines and cytokines.^134^ In contrast, TLR3 signals via TRIF, TRAF3 and IRF3 to induce type I IFN and via TRIF/TRAF6, MAPK and NF-κB to induce proinflammatory cytokines.^134^

#### RIG-I-like receptor

The RIG-I-like receptor (RLR) family of RNA sensors comprises RIG-I (also known as DDX58), MDA5 (also known as IFIH1) and LGP2 (also known as DHX58).^42^ RIG-I and MDA5 are composed of an N-terminal tandem caspase recruitment domain (CARD), a central helicase domain and a C-terminal domain. Upon activation by their ligand dsRNA, RIG-I and MDA5 multimerize and assemble their CARDs in a way that the CARD-containing adaptor molecule, MAVS protein (also known as interferon-β promoter stimulator 1, virus-induced signaling adaptor and caspase activation recruitment domain adaptor inducing IFN-β), multimerizes to form a signaling platform. This platform recruits and activates kinases (TBK1, IKKε and IKKα/β) to activate transcription factors including NF-κB and IRF3/5/7 that upregulate the transcription of genes coding for antiviral effector proteins, chemokines and cytokines.^46–49,145–150^ The RLR-induced antiviral response is dominated by type I IFN and IFN-stimulated genes. LGP2 lacks the CARDs and therefore is not able to induce a MAVS dependent signaling cascade. Studies about the effects of LGP2 on MDA5 and RIG-I activity are controversial. While initial studies using cell lines overexpressing LGP2 suggested that LGP2 inhibits RIG-I and MDA5 activation by competitively binding,^43,151^ later in vivo studies with LGP2 deficient mice (*Dhx58*^-/-^) exhibited a supporting role of LGP2 for MDA5 activation and to lesser extent also for RIG-I.^152^ The C-terminal domain of RIG-I recognizes and binds to the base paired end of 5’triphosphorylated (5’ppp) or diphosphorylated (pp) dsRNA.^39,153^ While RIG-I is activated by short (p)pp-dsRNA ( > 20 bp) but also long dsRNA,^39,153^ MDA5 recognizes only very long dsRNA( > 1000 bp).^154^ In contrast to RIG-I ligands, the exact RNA recognition motif of MDA5 is not well characterized and the minimal length and structure requirements of a biologically relevant dsRNAs remain unclear. By deamination of A to I, the adenosine deaminase acting on RNA 1 (ADAR1) resolves secondary structures and therefore prevents innate immune responses to endogenous RNAs by MDA5 with LGP2 as a co-receptor.^155–157^

#### Other RNA sensors

Analogous to the dsDNA recognizing AIM2 inflammasome, recently NLRP1 inflammasome was found to be activated by very long dsRNA poly(I:C) or (+)ssRNA virus infection resulting in consequent proteolytic activation of caspase-1, IL-1β and the pore forming GSDMD inducing pyroptosis.^52^ To prevent virus replication, antiviral effector proteins frequently target the translation of viral genes. PKR and OAS1 were found to be type I IFN-induced, and to bind and be activated by long ( > 60 bp) dsRNA.^23,24,26,27^ Upon dsRNA binding, PKR multimerizes and phosphorylates the translation initiation factor, eukaryotic initiation factor 2α, leading to inhibition of genome wide translation. Similarly, OAS1 oligomerizes on long dsRNA and subsequently synthesizes 2’,5’ oligomers of adenosine which serves as a second messenger that activates RNase L. RNase L degrades viral and endogenous RNA, including ribosomal RNA (rRNA) which also blocks genome wide protein production.^23,24,26,27^ By contrast, the 5’cap binding protein IFIT1^50,51^ recognizes hypomethylated cap-0 structures at the 5’end of viral mRNA and prevents recruitment of the cap binding translation initiation factor, eukaryotic initiation factor 4E, and therefore interferes with mRNA translation of cap-0 mRNA.^158^

## Ligands for nucleic acid sensors

### Non-self-DNA

Microbial DNA, primarily due to its unmethylated CpG motifs, was the first DNA type identified as an immunostimulant.^159–161^ This supports the idea that DNA sensing pathways can distinguish non-self- from self-DNA to defend against pathogens like bacteria, viruses and fungi, which act as immune danger signals (Table 1).^1,162^ Foreign DNA exposure can also occur through transplantation, transfusion, gut translocation and pregnancy.^163^

**Table 1:** Release of nucleic acids in critical illness

Critical illnesses	Nucleic acids	Samples	Findings	Ref.
ARDS	cfDNA	Human plasma	High levels in patients with severe influenza; positively correlated with CRP levels and disease severity.	^772^
			Increased in severe H1N1 IAV infection compared to mild cases; positively correlated with SOFA scores.	^773^
		Mouse BALF	Increased during MRSA-induced pneumonia.	^269^
			Increased in IAV-infected mice.	^303,304^
	Bacterial DNA	Human whole blood	Correlated with CRP value, WBC count and LOS in patients with community-acquired pneumonia.	^774^
		Human pleural fluid	Positively correlated with serum CRP, pleural fluid neutrophil count, pleural fluid glucose and LOS.	^775^
	mtDNA	Human whole blood	Associated with 28-day mortality in sepsis-associated ARDS cases but not in septic patients without ARDS.	^776^
		Mouse BALF	Peaked at day 4 post-infection in IAV-infected mice.	^298^
			Elevated in diABZI-induce ARDS.	^301^
	MPO-DNA complex	Human plasma	High levels in patients with severe influenza; positively correlated with disease severity.	^772^
			More in severe H1N1 IAV infection than mild H1N1 infection and healthy controls.	^773^
		Human bronchial aspirates	Correlated with severity of gram-negative bacterial pneumonia-induced ARDS; increased in non-survivors compared with survivors; correlated with IL-1β and caspase-1 levels in aspirates.	^280^
	Histone-DNA complex	Human plasma	Increased in severe H1N1 IAV infection compared to mild cases; positively correlated with SOFA scores.	^773^
	Neutrophil-derived DNA	Mouse BALF	Induced by intratracheal administration of STING agonist diABZI.	^301^
	Viral RNA	Human respiratory samples	The proportion of defective viral genomes in isolated viruses is lower in samples from severe or fatal IAV cases than in those from mild IAV patients.	^777^
	evRNA	Mouse serum and BALF	Microvesicle-containing miRNA-223 and miRNA-142 are increased post-intratracheal instillation of LPS or *Klebsiella pneumoniae*.	^778^
COVID-19	cfDNA	Human plasma	Increased in COVID-19 compared with influenza or respiratory syncytial virus infection.	^238^
			Higher in COVID-19 ICU patients than non-COVID-19 ICU patients.	^779^
			Increased in severe COVID-19 compared with non-COVID controls and COVID-19 convalescents.	^780^
			cfDNA fragmentation pattern reflects tissue injury.	^216^
			Distinct end-motif patterns of cfDNA in non-survived COVID-19 patients.	^217^
		Human serum	Increased in ventilated COVID-19 patients; correlated with CRP, D-dimer, LDH and neutrophil count.	^781^
			Related to disease severity; independent risk factor for mortality and need for intensive care.	^782^
	Bacterial DNA	Human whole blood	Higher in non-survived patients; positively associated with neutrophil count and plasma IFN-α level.	^783^
		Human plasma	Associated with secondary infections and mortality in COVID-19 patients.	^784,785^
			Increased in severe or ICU patients compared with moderate cases or healthy controls.	^786^
		Human serum	Increased in hospitalized patients; higher in severe and fatal cases.	^787^
	mtDNA	Human whole blood	Positively correlated with anti-spike IgG antibody titers and IFN-γ levels induced by T cells in elderly patients.	^788^
		Human plasma	Elevated in patients who decease or require ICU admission.	^789^
			Negatively associated with LVEF in COVID-19.	^790^
			Increased in COVID-19 cases compared with IAV or RSV cases.	^238^
			Increased in asymptomatic cases compared with symptomatic cases.	^791^
			Significantly lower abundance and longer fragment size of cell-free mtDNA.	^217^
		Human serum	Increased in COVID-19 patients compared with healthy individuals and patients with comorbidities.	^376,792^
			Negatively correlated with SpO_2_/FiO_2_ ratio; associated with the requirement for oxygen therapy.	^793^
		Human peripheral blood leukocytes	Lower in type 2 diabetes patients with post-COVID-19 syndrome than those without; independent factor associated with post-COVID-19 syndrome development in type 2 diabetes patients.	^794^
		Human RBCs	Elevated in COVID-19 patients compared with healthy individuals; increased with disease severity; correlated with anemia.	^59^
	nDNA	Human plasma	Elevated in COVID-19 patients compared with patients with IAV and RSV infection; higher levels in non-survived patients compared with survived patients; positively correlated with COVID-19 severity.	^238^
			Increased in symptomatic patients compared with asymptomatic cases.	^791^
		Human serum	Negatively correlated with SpO_2_/FiO_2_ ratio; associated with the requirement for oxygen therapy or mechanical ventilation during hospital stay.	^793^
	MPO-DNA complex	Human plasma	Increased in COVID-19 patients compared with healthy donors and convalescent patients; elevated in COVID-19 non-survivors compared with COVID-19 survivors; correlated with SOFA score.	^795^
			Increased in severe COVID-19 compared with non-COVID controls and COVID-19 convalescents.	^780^
			Elevated in critical COVID-19 patients; correlated with PaO_2_/FiO_2_ ratio and SOFA score.	^676^
			Increased in COVID-19 patients compared with healthy controls; associated with disease severity.	^404^
		Human serum	Increased in COVID-19 patients; higher levels in hospitalized patients with mechanical ventilation.	^781^
			Increased in COVID-19 patients compared with healthy controls.	^782^
	citH3-DNA complex	Human plasma	Elevated in critical COVID-19 patients; correlated with CRP, the neutrophil/lymphocyte ratio and ROX index; predictive potential for hospitalization among outpatients with non-severe COVID-19.	^676^
		Human BALF	Increased in patients with CAPA compared with those without; associated with increased 90-day mortality.	^796^
	NE-DNA complex	Human serum	Increased in COVID-19 patients compared with healthy controls.	^782^
	cfRNA	Human plasma	380 upregulated cfRNAs in COVID-19 patients compared to healthy controls; 7 upregulated cfRNAs show potential to predict COVID-19.	^797^
	Viral RNA	Human blood	Persistent SARS-CoV-2 RNA in post-COVID-19 condition.	^798^
			Indicator for severe cases.	^799^
			Higher in COVID-19 non-survivors than survivors.	^800^
		Human plasma	More SARS-CoV-2 RNAemia in severe cases than in mild cases.	^801^
			Higher loads in critically ill patients; positively correlated with levels of CXCL10, LDH, IL-10, IL-6, IL-15 and MPO; negatively correlated with oxygen saturation and lymphocytes and monocyte counts.	^802^
		Human serum	Associated with increased mortality risk in hospitalized COVID-19 patients.	^803^
	Host mRNA	Human whole blood	Prolactin mRNA/TLR3 mRNA ratio is associated with disease severity and prognosis.	^383^
	mtRNA	Human blood	Small (in contrast to long non-coding) mtRNAs change expression during COVID-19 recovery.	^804^
	miRNA	Human plasma	Associated with COVID-19-related death in cancer patients.	^805^
			miRNA-195-5p is correlated with severity of COVID-19.	^801^
	evRNA	Human plasma	Viral RNA is detected in plasma exosomes from COVID-19 patients.	^374,806^
			miRNA-21 and let-7b in EVs are increased in COVID-19 patients compared to healthy controls and associated with disease severity.	^404^
Sepsis	cfDNA	Human plasma	Associated with severity of sepsis; potential to distinguish between patients with infection and those with sepsis.	^807^
			Highly predictive of mortality in sepsis; enhanced accuracy with protein C and MODS scores.	^808^
			Elevated total and cardiac-specific cfDNA in sepsis; cardiac cfDNA may predict 90-day mortality.	^240^
			Correlates with vasopressor-inotropic score and SOFA score in septic shock.	^809^
			Positively correlated with plasma MPO levels.	^528^
			Elevated in septic ICU patients; increased short cfDNA fragments; correlates with SOFA score; useful for sepsis diagnosis and mortality prediction.	^218^
			Higher in ICU non-survivors; independent predictor of ICU mortality; correlates with SOFA score.	^810^
			Potential biomarker for prediction and early diagnosis of sepsis in burn-injured patients.	^811^
			Elevated in septic ICU patients especially those with AKI; higher in non-survivors; correlates with caspase-3, IL-6, and IL-18 levels.	^812^
			Increased in septic shock patients compared with healthy individuals.	^813^
			More short fragments in sepsis patients compared to those in non-sepsis controls.	^218^
			Increased short fragments 30-167 bp in septic patients, with higher 30-147 bp ratios in those with sepsis-induced AKI or coagulation dysfunction.	^219^
		Human serum	Increased in sepsis patients compared with healthy controls.	^683^
			Increased in abdominal sepsis patients; positively correlated with enterocyte injury markers.	^517^
			Elevated in septic patients; positively correlated with PaO_2_/FiO_2_ and coagulation markers.	^493^
			Associated with mortality in patients with sepsis-induced AKI.	^814^
			More in sepsis patients than non-sepsis patients and healthy controls; positively correlated with SOFA score, levels of PCT and indicators of coagulation and kidney damage; independent risk factor for sepsis.	^815^
		Mouse plasma	Increased in CLP-treated mice compared with sham group.	^493,813,816^
		Mouse serum	Elevated in septic mice compared with the control group.	^683^
			Elevated mouse genomic DNA in severe CLP model compared with sham group and moderate CLP model.	^719^
		Mouse BALF	Elevated in septic mice compared with the control group.	^481,683,813^
	Bacterial DNA	Human whole blood	Higher in patients with intravascular *Staphylococcus aureus* infection than those with extravascular infection.	^817^
			Associated with sepsis-associated mortality.	^818^
		Human plasma	Decreased diversity in septic ICU patients; related to SOFA score, sepsis and 28-day mortality.	^218^
		Mouse serum	Increased in CLP mouse model.	^166^
	Viral DNA	Human whole blood	Increased in septic cases than non-septic critically ill cases and healthy individuals.	^819^
		Human plasma	Viral DNAemia for cytomegalovirus, human herpesvirus 6, BK polyomavirus and human adenovirus is associated with increased sepsis mortality.	^820^
			Associated with immunosuppression and the risk of secondary infection in septic children.	^821^
			Increased in septic cases than non-septic critically ill cases and healthy individuals; associated with risk of fungal and opportunistic bacterial infections.	^819^
			Associated with hyperferritinemia in children with severe sepsis.	^822^
	mtDNA	Human plasma	Increased with severity of sepsis.	^807^
			Increased in patients with MDR bacterial septic shock; elevated in those who die compared to survivors.	^469^
			Higher levels in sepsis children than non-sepsis critically ill children and healthy controls.	^823^
			Elevated in early sepsis and decreased after acute phase; higher in non-survivors and those with AKI/RRT; improves RRT and mortality prediction with creatinine and APACHE II, respectively.	^824^
			Elevated at 48 h in septic patients; linked to higher ARDS risk.	^540^
			Higher levels in sepsis patients than healthy controls.	^465,466^
		Human serum	Higher levels in sepsis patients compared with healthy controls; associated with elevated 30-day mortality.	^825^
			Higher in sepsis-induced ALI; correlated with ventilation duration and injury severity.	^488^
		Human RBCs	Higher RBC-associated mtDNA in septic patients with anemia; linked to anemia and severity in COVID-19 with secondary sepsis.	^59^
		Mouse plasma	Increased after CLP compared to sham group.	^466,513^
			Increased after LPS injection.	^466,483^
		Mouse plasma and RBCs	Higher levels of RBC-bound mtDNA than plasma mtDNA in septic mice.	^59^
		Mouse serum	Increased in CLP-treated mice.	^518^
		Mouse peritoneal fluid	Increased after CLP.	^513^
		Mouse BALF	Increased with peak at 24 h post-LPS administration; correlated with TNF-α, IL-1β, and IL-6 in BALF.	^481^
	nDNA	Human plasma	Increased with sepsis severity; higher in ICU than non-ICU patients; improves diagnosis when combined with SOFA.	^807^
			Elevated early in sepsis and decreased post-acute phase; higher in 28-day non-survivors and in patients with AKI/RRT; enhances RRT and 28-day mortality prediction with serum creatinine and APACHE II scores, respectively.	^824^
			Higher levels in sepsis patients than healthy controls.	^465,826^
			Correlates with SOFA score and positive 16S rDNA; high nDNA concentrations are linked to sepsis at admission.	^826^
	MPO-DNA complex	Human plasma	Elevated in septic patients compared with healthy controls.	^493^
			Increased in septic shock patients; linked to mortality and positively correlated with SOFA scores.	^813^
		Human serum	Elevated in sepsis; higher in non-survivors; positively linked to SOFA score.	^683^
			Higher in sepsis patients than non-sepsis and healthy controls; positively correlates with SOFA scores, PCT, coagulation and organ damage markers; improves CRP’s diagnostic accuracy in sepsis.	^815^
		Mouse plasma	Increased in CLP-treated mice.	^493,813^
		Mouse serum	Increased in CLP-treated mice.	^683^
		Mouse BALF	Higher in LPS-stimulated mice compared to sham controls.	^494^
	citH3-DNA complex	Human serum	Increased in abdominal sepsis patients compared with healthy individuals; positively correlated with markers of enterocyte injury, serum D-lactate and intestinal fatty-acid binding protein.	^517^
	NE-DNA complex	Mouse BALF	Higher in septic mice compared with sham controls.	^494^
	cfRNA	Human plasma	Increased in septic patients compared with healthy volunteers.	^827,828^
		Mouse plasma	Increased in CLP-subjected mice with a peak at day 7.	^505,827^
	Host RNA	Human blood	Transcriptomic sepsis scores based on host mRNA levels can help diagnose sepsis.	^829^
	miRNA	Human plasma	miRNA-146a-5p, miRNA-10a-5p, miRNA-22-3p and miRNA-122-5p are abundant in septic patients; miRNA-146a-5p is associated with sepsis outcome predictors blood lactate and coagulopathy.	^827^
			More miRNA-146-3p, miRNA-147b, miRNA-155 and miRNA-223 in patients with sepsis and sepsis shock compared to healthy controls; potential biomarkers for sepsis diagnosis and sepsis shock prediction.	^830^
		Mouse plasma	miRNA-146a-5p, miRNA-10a-5p, miRNA-22-3p and miRNA-122-5p are abundant in CLP-treated mice.	^827^
			miRNA-146a-5p and miRNA-145–5p are increased in CLP-treated mice.	^505^
		Mouse BALF	miRNA-146a-5p is increased in CLP-challenged mice.	^496^
	evRNA	Human plasma	Correlates with sepsis severity; higher in septic shock; may predict septic shock after ICU admission.	^831^
Trauma	cfDNA	Human plasma	Higher levels in trauma patients than healthy individuals.	^528^
			Correlated with endotheliopathy markers syndecan-1 and thrombomodulin in trauma patients; higher in non-survivors than in survivors.	^524^
			Increased in moderate and severe trauma cases compared with healthy controls; higher in penetrating trauma than blunt trauma.	^527^
			Higher levels in severe trauma patients at admission to the emergency room than in patients with moderate trauma.	^525^
		Human serum	Increased levels in patients at 1, 4–12 and 48–72 h post-trauma compared to healthy controls; higher in those with both traumatic brain injury and extracranial injuries than in patients with isolated injuries.	^832^
			Higher levels in severe trauma patients at admission to the emergency room than in patients with moderate trauma.	^525^
			Elevated in children with burn injuries than in healthy controls; higher levels in full-thickness burns than in partial-thickness burns.	^833^
		Dog plasma	Higher levels in dogs with trauma than healthy dogs.	^834^
	mtDNA	Human plasma	Elevated levels after admission in trauma patients; linked to higher ARDS risk.	^540^
			Higher levels in the elderly trauma patients than young patients.	^835^
		Human plasma and RBCs	Increased mtDNA levels and the ratio of plasma mtDNA to RBC-bound mtDNA in trauma patients compared with healthy controls.	^58^
	NETs	Human neutrophils	Formation after trauma and subsequent surgery; mainly containing mtDNA.	^836^
	cfRNA	Mouse serum	Detectable in mice with lung contusion.	^549^
		Mouse BALF	Detectable in mice with lung contusion.	^549^
	miRNA	Mouse plasma	miRNA-7a-5p, miRNA-142, let-7j, miRNA-802 and miRNA-146a-5p induce proinflammatory macrophage response.	^548^
AMI	cfDNA	Human plasma	Cardiac-specific cfDNA is significantly elevated in acute ST-elevation MI, correlates with troponin and creatine phosphokinase, declines after revascularization, and is undetectable in healthy controls.	^240^
			DNA fragments of 150-200 bp, 300-400 bp, and 500-600 bp are increased in patients; the 150-200 bp/500-600 bp ratio in MI is significantly higher than those in other cardiac diseases.	^220^
		Mouse plasma	Increases at 6 h and returns to sham level at 24 h post-MI.	^837^
	mtDNA	Human plasma	Higher levels in the AMI group on hospital day 1 compared with non-MI controls (with MI risk) and healthy individuals; decreased shortly after PCI.	^838^
	nDNA	Human plasma	Increased in the AMI patients on hospital day 1 compared with non-MI individuals (with MI risk) and healthy controls; decreased shortly after PCI.	^838^
	citH3-DNA complex	Human plasma	Increased in MI patients compared with healthy controls.	^837^
		Mouse plasma	Increased at 6 h post-MI.	^837^
	MPO-DNA complex	Human serum	Potential to predict adverse left ventricular remodeling at 6 months after primary PCI in ST-elevation MI patients.	^839^
	NE-DNA complex	Human plasma	Increased in MI patients compared with healthy controls.	^837^
		Mouse plasma	Increased at 6 h post-reperfusion.	^837^
	mtRNA	Human plasma	The mitochondrial long noncoding RNA uc022bqs.1 is downregulated early after MI but upregulated during later stage.	^840^
	miRNA	Human blood	Various miRNAs in circulation function as diagnostic and prognostic biomarkers for AMI.	^841,842^
Myocardial IR	cfDNA	Human plasma	Elevated in post-CPB samples compared with pre-CPB samples.	^561^
		Human serum	Increased in CA patients 1 week after ROSC; higher in non-survivors; correlated with APACHE II score; predictors of 28-day mortality on day 1 after ROSC.	^843^
		Mouse plasma	Increased at 5 and 15 min after reperfusion compared with sham controls.	^561^
	mtDNA	Mouse plasma	The major kind of cfDNA that is increased at 5 and 15 min after reperfusion compared with sham group.	^561^
		Rat plasma	Elevated in myocardial IR rat compared with control counterparts.	^559^
	Host RNA	Mouse plasma	Increased post-myocardial IR, including miRNA‐208a, miRNA‐499, miRNA‐1 and miRNA‐133.	^574^
Hepatic IR	cfDNA	Human plasma	Elevated post-liver transplantation and rapidly decreased within a week; linked to CRP, leukocytosis, granulocytosis and liver abscess followed by sepsis; independent predictor of 1-year survival.	^844^
		Human serum	Peaked on day 1 post-liver transplantation; declined with recovery; correlated with ALT, AST and TBIL.	^845^
		Mouse serum	Increased at 1 and 6 h after reperfusion compared with sham controls.	^585^
			Increased in hepatic IR mice compared with sham group; positively correlated with serum ALT and AST.	^846^
			Higher in hepatic IR mice than sham controls.	^586^
	mtDNA	Human plasma	Elevated in liver transplant patients; higher in early postoperative allograft dysfunction; correlated with plasma AST and ALT.	^847^
		Mouse serum	Increased at 6 h after reperfusion compared with sham controls.	^848^
			Higher in hepatic IR mice than sham controls.	^586^
	citH3-DNA complex	Mouse serum	Increased in hepatic IR mice compared to sham group; positively correlated with serum ALT and AST.	^846^
Renal IR	cfDNA	Human urine	Donor-derived cfDNA is increased in renal transplant patients with acute rejection, correlating with protein/creatinine ratio and glomerular filtration rate.	^849^
		Mouse plasma	Increased at 24 h after reperfusion compared with sham group.	^688^
			Increased at 6 h post-reperfusion compared with sham-operated mice.	^850^
		Mouse serum	Increased in renal IRI group compared with sham group.	^687,851^
		Rat plasma	Increased at 48 h after reperfusion compared with sham controls; negatively correlated with GFR.	^852^
		Rat urine	Elevated at 48 h post-reperfusion compared with sham controls; negatively correlated with GFR.	^852^
	mtDNA	Human plasma	Increased in renal transplant cases compared with healthy individuals.	^853^
			Released during living donor kidney transplantation; derived from kidney or the whole body; potential to predicted 1- and 24-month eGFR and acute rejection episodes.	^854^
		Human urine	Increased in renal transplant patients; correlated with cold ischemia time and renal function; elevated in DGF patients.	^853^
		Mouse plasma	Increased in mice with renal IR compared with sham controls.	^592,853^
		Mouse urine	Elevated in ischemic AKI mice compared with the sham group.	^853^
	NE-DNA complex	Mouse plasma	Increased at 6 and 15 h post-reperfusion compared with sham group.	^850^

*AKI* acute kidney injury, *ALI* acute lung injury, *ALT* alanine aminotransferase, *AMI* acute myocardial infarction, *APACHE* acute physiology and chronic health evaluation, *ARDS* acute respiratory distress syndrome, *AST* aspartate aminotransferase, *BALF* bronchoalveolar lavage fluid, *bp* base pair, *CA* cardiac arrest, *CAPA* COVID-19-associated pulmonary aspergillosis, *cfDNA* cell-free DNA, *cfRNA* cell-free RNA, *citH3* citrullinated histone H3, *CLP* cecal ligation and puncture, *COVID-19* coronavirus disease 2019, *CPB* cardiopulmonary bypass, *CRP* C-reactive protein, *CXCL10* C-X-C motif chemokine ligand 10, *DGF* delayed graft function, *diABZI* dimeric amidobenzimidazole, *DNA* deoxyribonucleic acid, *eGFR* estimated glomerular filtration rate, *evRNA* extracellular vesicle RNA, *FiO*_*2*_ fraction of inspired oxygen, *GFR* glomerular filtration rate, *IAV* influenza virus A, *ICU* intensive care unit, *IFN* interferon, *IgG* immunoglobulin G, *IL* interleukin, *IR* ischemia/reperfusion, *IRI* ischemia/reperfusion injury, *LDH* lactate dehydrogenase, *LOS* length of hospital stay, *LPS* lipopolysaccharides, *LVEF* left ventricular ejection fraction, *MDR* multi-drug resistant, *MI* myocardial infarction, *miRNA* microRNA, *MODS* multiple organ dysfunction syndrome, *MPO* myeloperoxidase, *mRNA* messenger RNA, *MRSA* methicillin-resistant *Staphylococcus aureus*, *mtDNA* mitochondrial DNA, *mtRNA* mitochondrial RNA, *nDNA* nuclear DNA, *NE* neutrophil elastase, *NETs* neutrophil extracellular traps, *PaO*_*2*_ arterial partial pressure of oxygen, *PCI* percutaneous coronary intervention, *PCT* procalcitonin, *RBCs* red blood cells, *rDNA* ribosomal DNA, *Ref*. reference, *RNA* ribonucleic acid, *ROSC* restoration of spontaneous circulation, *ROX* respiratory rate-oxygenation, *RRT* renal replacement therapy, *RSV* respiratory syncytial virus, *SARS-CoV-2* severe acute respiratory syndrome coronavirus 2, *SOFA* Sequential Organ Failure Assessment, *SpO*_*2*_ oxygen saturation, *TBIL* total bilirubin, *TLR* toll-like receptor, *TNF-α* tumor necrosis factor α, *WBC* white blood cell

**Fig. 4:**
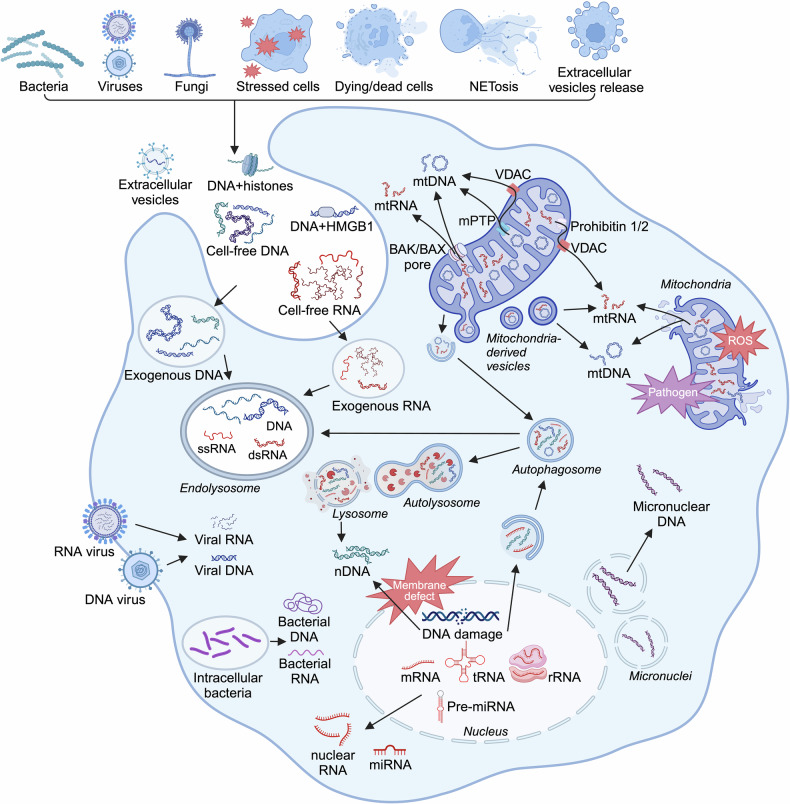
Ligands for nucleic acid sensors. Mislocated nucleic acids are predominantly ascribed to pathogen infection, cell stress and damage, cell death and active release mechanisms such as NETosis and extracellular vesicles. Cell-free DNA could bind to proteins such as histones and HMGB1. In addition, the impairment of subcellular components including mitochondria, nuclei, micronuclei and lysosome causes cytosolic leakage of host nucleic acids. Figure created with BioRender.com. ROS reactive oxygen species

DNA sensing mediates antibacterial immunity via bacterial DNA recognition.^164^ During infections, microbial breakdown and pathogen-derived membrane vesicles release bacterial DNA into the circulation (Fig. 4).^165^ Intestinal microbial DNA also contributes to the increase in circulating bacterial DNA via gut translocation.^166^ Nevertheless, bacterial DNA itself is considered non-pathogenic and non-lethal.^167^ Actually, the existence of bacterial DNA is observed in the blood of both healthy volunteers and sepsis patients, yet the bacterial diversity in these two groups presents significant difference.^168^ Thus, bacteria might translocate into the bloodstream continuously, but they alone are probably not enough to cause severe disease. As for viral infection, viral genome recognition is the very first step of host antiviral defense. DNA sensors recognize viral genomes from DNA viruses, regulating inflammatory cascades and programmed cell death.^169–171^ In addition, antifungal immunity also involves fungal DNA detection.^172^

### Self-DNA

The source of endogenous DNA plays a key role in critical illness. DNA from various origins activates different sensors—TLR9 mainly detects unmethylated CpG-rich mtDNA,^173^ while nuclear DNA (nDNA) or micronuclear DNA in the cytoplasm may activate cGAS-STING pathway,^6^ however this concept is still debated.^174–176^ Cellular compartmentalization also affects sensor access, with nuclear sensors detecting DNA directly. mtDNA is well-studied in this context, but the roles of nDNA and micronuclear DNA need further research. Identifying DNA sources may aid in developing therapies to reduce harmful DNA release and accumulation in critical illness. The following sources of DNA might play a role in critical illness.

#### Cytosolic self-DNA

The impairment of subcellular components such as mitochondria and nuclei causes intracellular disorder and the release of self-DNA into the cytoplasm that activates the same signaling pathways as non-self-DNA (Fig. 4). The aberrant accumulation of mislocated self-DNA activates endogenous DNA sensing systems, resulting in cytokine release and cell death that initiates immune responses and clears out damaged cells, respectively.

mtDNA is one of the best characterized self-DNA ligands. Under mitochondrial stress, the opening of mitochondrial permeability transition pores (mPTP) permits mtDNA to translocate into the cytoplasm (Fig. 4).^177,178^ The pro-apoptotic proteins Bcl-2-associated protein X (BAX) and Bcl-2 homologs antagonist/killer (BAK) also contribute to mtDNA release through apoptotic pores on mitochondrial outer membrane.^179^ Additionally, voltage-dependent anion channel (VDAC) oligomerizes under stress and forms mitochondrial pores that allow mtDNA release.^180^ BAX/BAK- and VDAC-mediated mechanisms have distinct functions: BAX and BAK are at the core of intrinsic pathway of apoptosis,^181^ while VDAC primarily regulates metabolite and ion transport between mitochondria and cytosol.^182^ mtDNA release through BAX/BAK pores activates cGAS-STING-dependent IFN induction, but apoptotic caspases suppress IFN induction.^183,184^ In contrast, VDAC-mediated mtDNA release strongly promotes inflammation and immune activation.^185^ Besides, oxidative damage also leads to subsequent mtDNA release.^186–188^

Genomic DNA is a notable source of immunostimulatory DNA. The accumulation of cytoplasmic chromatin fragments, or nDNA, results mainly from nuclear membrane defects, impaired lysosomal degradation, autophagy and mitochondrial oxidation.^6^ In addition, genomic DNA released from micronuclei could be detected by cytosolic DNA sensors and drive inflammatory cytokine storms.^189^ Micronuclei are unstable nuclear envelopes easy to rupture and release entrapped chromosomes, of which formation is initiated by DNA damage, chromosomal mis-segregation, mitotic dysfunction and cell fusion, especially during bacterial and RNA viral infections.^6,189–191^

#### Cell-free self-DNA

Cell-free DNA (cfDNA) is present in the bloodstream of healthy individuals, primarily originating from host cells like white blood cells.^192^ Thus, DNAemia^193^ – DNA detected in the blood – does not inherently indicate illness. However, cfDNA patterns change significantly in pathological conditions, especially in critical illnesses (Table 1).

Various cellular breakdown mechanisms lead to cfDNA release, including apoptosis, autophagy, necrosis, necroptosis, pyroptosis and NETosis (Fig. 4).^132,163,194^ In particular, NETosis is a special form of cell death in which neutrophils immobilize and kill invading pathogens by intentionally releasing host NA-based net-like structures, termed neutrophil extracellular traps (NETs).^195^ The active release of DNA occurs not only during NETosis, but also in the form of erythroblast enucleation, virtosomes and extracellular vesicles (EVs), including exosomes, microvesicles and apoptotic bodies.^196,197^ Exosomes form through inward plasma membrane invagination, while microvesicles and apoptotic bodies emerge via direct plasma membrane budding and membrane blebbing during apoptosis, respectively.^198–200^ Of note, over 90% of plasma cfDNA is located in plasma exosomes.^201^ DNA cargo in vesicular transport includes genomic DNA, mtDNA and even viral DNA, collectively termed extracellular vesicle DNA.^202–204^ Importantly, EVs are not only derived from host cells but also from pathogens.^205^ Although the role of EVs in critical illness has been well reviewed,^206–208^ their cargo DNA is relatively less discussed. In particular, the complex composition of EVs poses a challenge to confirm the contribution of contained DNA. In general, DNA in the extracellular environment undergoes internalization by immune cells and enhances innate immune responses and cell death, addressing pathological conditions but also accelerating disease progression.^209–212^

While the cfDNA fragmentation profile aids in early cancer detection,^213^ monitoring transplant rejection,^214^ and non-invasive prenatal testing,^215^ its role in critical illness remains underexplored. A study found that patients with coronavirus disease 2019 (COVID-19) exhibit higher levels of short cfDNA fragments (< 150 bp), fewer intermediate fragments (150-250 bp), and similar levels of long fragments ( > 250 bp) compared to controls, with severe cases showing a marked reduction in long fragments.^216^ Specific end motifs, 5′-CCCA and CT-5′-CC, are elevated in COVID-19. After treatment, short fragments decrease in severe cases, while long fragments increase in all cases. Additionally, cfDNA fragmentation signals align with tissue-specific open chromatin regions, indicating organ injury in COVID-19.^216^ Another study found that non-surviving COVID-19 patients display distinct “CC” start motifs in cfDNA and lower levels of longer cell-free mtDNA fragments in plasma. These fragmentation patterns may serve as biomarkers to distinguish between survivors and non-survivors.^217^ cfDNA fragmentation in sepsis patients shows more short fragments (30-167 bp) compared to controls, with a diverse distribution of 147-167 bp fragments.^218,219^ Patients with sepsis-induced acute kidney injury (AKI) or coagulation issues have an even higher ratio of 30-147 bp fragments.^219^ In myocardial infarction (MI), increased cfDNA fragments, likely from apoptosis, show a unique 150-200 bp/500-600 bp ratio, suggesting potential as an MI biomarker.^220^

Importantly, the modifications of DNA exert significant impacts on DNA recognition, including protein modification, oxidization and methylation. In critical illness, the stimulatory self-DNA could be naked or protein bound. The association with proteins is crucial for the DNA recognition and immune activation. It is assumed that circulating genomic DNA is more likely bind to histones.^221^ Especially during the formation of NETs, nDNA and mtDNA are dramatically released and interact with citrullinated histone H3, myeloperoxidase and neutrophil elastase.^195^ Another non-histone protein, high mobility group box 1 protein (HMGB1), also binds to nDNA and mtDNA.^222,223^ Free HMGB1 is a key player in critical diseases,^224–226^ yet it is unclear whether its complex with DNA also makes a contribution. Likewise, mtDNA could be released in associated with mitochondrial transcription factor A (TFAM).^227^ A recent study showed that TFAM mediates autophagic removal of leaked mtDNA to restrict inflammation,^228^ however, the role of TFAM-bound DNA in critical illness remains poorly understood. Both protein-associated and naked DNA are ligands for TLR9, cGAS and AIM2, while the protein binding further augments DNA sensor activation and amplifies the immunogenic potential of DNA.^80,222,227,229,230^ Moreover, DNA-binding proteins may improve the stability of extracellular DNA while naked DNA is more likely to be degraded by circulating DNases.^221,231^ However, most of the studies showing the contributing role of self-DNA to critical illness do not directly demonstrate the protein modification of self-DNA, possibly owning to the difficulty in delineating naked DNA and protein bound DNA accurately.

Oxidation and methylation are key DNA modifications. In critical illness, oxidative stress damages self-DNA and promotes inflammation.^232,233^ For example, influenza A virus (IAV) proteins trigger oxidized DNA release in macrophages, activating the AIM2 inflammasome,^234^ while lipopolysaccharides (LPS) in in vitro sepsis models induces oxidized mtDNA release that activates the cGAS-STING pathway.^235,236^ Liver ischemia/reperfusion (IR) also triggers cGAS-STING pathway in macrophages through DNA oxidation.^237^ DNA methylation helps differentiate self- from non-self-DNA, with endogenous DNA typically methylated to prevent overactivation while unmethylated mitochondrial DNA can stimulate TLR9.^173^ DNA methylation signatures trace cfDNA origins, correlating with severity and outcomes in COVID-19^238,239^ as well as indicating cardiomyocyte death in sepsis and MI.^240^

### Cell-free RNA

Cell-free RNA (cfRNA) is composed of mRNA, long non-coding RNA, transfer RNA (tRNA), microRNA (miRNA), circular RNA and so on.^241^ In common with cfDNA, cfRNA is derived from exogenous and endogenous sources via similar mechanisms. Likewise, RNA species packed in EVs make up an important form of cfRNA termed extracellular vesicle RNA.^205,242^ The release of cfRNA in blood and fluids could be found in critical illnesses, which displays clinical significance as diagnostic and prognostic biomarker for disease management (Table 1).

### Cytosolic non-self-RNA

NA receptors are the first line of antiviral defense. Since NAs are the main and critical component of viruses, they represent a logic target structure for antiviral immunity. Due to the need of RNA template-dependent replication of RNA, RNA viruses cannot avoid the generation of dsRNA during their live cycle. However, viral RNA binding proteins can minimize the length of emerging dsRNA. Using an antibody that recognizes 40mer dsRNA, Weber et al. showed that (+)ssRNA, dsRNA and also dsDNA-viruses (herpes simplex virus, HSV) produce substantial amounts of dsRNA, while no dsRNA ( > 40mer) could be observed in (-)ssRNA viruses.^243^ While long dsRNA can stimulate all above mentioned receptors except IFIT1 and TLR8, the genomic RNA of (-)ssRNA viruses only stimulate RIG-I^153,244,245^ or ssRNA receptors including TLR7 and 8.^33,34^

Notably, it was found that (-)ssRNA viruses in single clonal virus preparations are also poor stimulators of RIG-I, which recognizes short (p)pp-dsRNA. However, batch expansion of the same (-)ssRNA viruses led to the generation of virus-derived defective interfering particles containing aberrant long copy back dsRNA that is generated by errors of the viral RNA polymerase and represent a very strong RIG-I activator.^246^

The dsRNA from DNA viruses is generated by converging transcripts of opposite genome strands. Therefore, HSV-1 stimulates not only DNA receptors but also RNA receptors (including RIG-I-like helicases and TLR3), and the recognition of HSV infection by the dsRNA receptor TLR3 plays a pivotal role in the antiviral immune response against HSV in the brain.^247–250^

### Cytosolic self-RNA

In eukaryotic cells, RNA is generated in the nucleus (nuclear RNA), from where it is transported to the cytosol and the endosymbiotic mitochondria (Fig. 4). The nuclear RNA is highly modified at internal and/or the 5’ends. Base conversion by ADAR1 in endogenous RNA (regions) with strong secondary structure such as genomic Arthrobacter luteus elements was suggested to prevent recognition by dsRNA receptors like MDA5 or PKR.^251,252^ However, a direct inhibition of a pattern recognition receptor by ADAR1 without the need of RNA editing was also reported.^253^ Modifications at or near the 5’end of all RNA types prevent recognition by RIG-I or IFIT1, as tRNA and rRNA possess 5’monophosphorylated ends, which were shown to prevent RIG-I activation.^254^ 5’cap1/2 modifications, especially the N1-2’O-methylation are critical to prevent recognition of capped RNA such as mRNA by RIG-I or IFIT1.^50,51,244,255^ By contrast, RNAs generated in the endosymbiotic mitochondria (mitochondrial RNA, mtRNA) rather resemble prokaryotic RNA concerning structure and modification. Human mitochondria express multiple 16.6-kb circular dsDNA genome copies that are bidirectionally transcribed. The mitochondrial genome encodes 13 mt-mRNAs, two mt-rRNAs and 22 mt-tRNAs.^256^ The mt-tRNAs are excised from the two long polycistronic precursor RNAs by RNase P and Z, leading to the release of the mt-mRNAs, which are located between the tRNAs on the mitochondrial genomic DNA strands.^257^ RNase Z cleavage results in 3’OH and 5’P termini, thus generating 5’phosphorylated mt-mRNAs.^258^ Mitochondria have a high content of dsRNA which, if released to the cytosol in the situation of mitochondrial barrier impairment, are accessible and recognized by MDA5 and PKR.^259,260^ By contrast, recognition of long mitochondrial dsRNA species such as SncmtRNA^261^ by RIG-I is prohibited by the presence of a 5’monophosphorylated end.^254^

## Nucleic acid sensing in ARDS

ARDS is a rapidly progressive condition in critically ill patients with a mortality up to 40%.^262^ The risk factors of ARDS include pneumonia, non-pulmonary sepsis, aspiration and trauma. To date, clinicians and scientists are still facing the challenge of developing novel and effective clinical strategies for ARDS owning to its heterogeneity of etiology, progression and individual therapeutic response. In this section, we mainly focus on the activated NA sensing in pneumonia-related ARDS (Table 2), while sepsis-induced acute lung injury (ALI) and ARDS will be discussed in the corresponding sections.

**Table 2:** Nucleic acid sensing in critical illness

Illnesses	Pathways	Nuclear acids	Models/cells	Pathogens/stimuli	Interventions on pathways	Main findings	Ref.
ARDS	TLR9	cfDNA	Mice	MRSA	TLR9 knockout	Elevated cfDNA in BALF activates TLR9, triggers inflammation, reduces bacterial load and inhibits lung consolidation.	^269^
		Bacterial DNA	Mice	CpG-DNA	TLR9 knockout	Bacterial CpG-ODN causes lung inflammation via TLR9.	^266^
			EA.hy926 cells; human PMNs	*E. coli* DNA; LPS	ODN A151	*E. coli* DNA promotes PMN-endothelial adherence and increases permeability possibly via TLR9.	^267^
			RAW 264.7 cells; mouse BMDMs	*Staphylococcus aureus* membrane vesicles	TLR9/MyD88/IRF3/IRF7 knockout; endosomal acidification inhibitor chloroquine	*Staphylococcus aureus* secretes vesicles with bacterial DNA that activates TLR9-dependent IFN response in macrophages.	^165^
		Fungal DNA	Mice; human pDCs	*Aspergillus fumigatus* DNA	TLR9 knockout; CpG methylase; CpG-specific endonucleases	TLR9 detects CpG motifs in *Aspergillus fumigatus* DNA and triggers the secretion of proinflammatory cytokines from DCs.	^172^
		mtDNA	Human PMNs	*E. coli*	-	CpG-DNA and mtDNA inhibit phagocytosis-induced apoptosis and promote neutrophil elastase release via TLR9.	^855^
			Mice; mouse alveolar macrophages	IAV	TLR9 knockout	Influenza-induced mtDNA leakage in lung structural cells activates TLR9-mediated inflammation.	^295^
	TLR9/STING	mtDNA; NETs	Mice; mouse BMDMs; human airway epithelial cells	STING agonist diABZI	cGAS/STING/IFNAR/TLR9/AIM2 knockout; cGAMP; DNase I; PAD inhibitor Cl-amidine	Airway exposure to diABZI induces mtDNA release, NET formation, DNA sensor upregulation and STING-dependent type I IFN response in lung, together with TLR9-mediated neutrophilic inflammation, PANoptosis and ARDS.	^301^
	TLR9/ZBP1	mtDNA	Mice; mouse BMDMs and alveolar macrophages	LPS	ZBP1 siRNA	LPS induces ZBP1-mediated necroptosis, releasing mtDNA that activates TLR9 and NF-κB in macrophages and causing lung injury in mice.	^486^
	cGAS-STING	Bacterial DNA	Mice; mouse BMDMs; human PBMCs; THP-1 cells	*Legionella pneumophila*; bacterial DNA	STING knockout; cGAS siRNA	cGAS-STING pathway is required for macrophage’s immune response towards *Legionella pneumophila* DNA and microbial clearance in mouse lungs.	^273^
			Mice; mouse BMDMs	*Legionella pneumophila*	IFN-β/IFNAR knockout	*Legionella pneumophila* releases bacterial DNA into macrophages, activating cGAS-STING and triggering IFN response.	^272^
			Mice; mouse BMDMs; human PBMCs	*Streptococcus pneumoniae*; bacterial DNA	cGAS/STING knockout; cGAS siRNA	cGAS-STING pathway senses pneumococcal DNA in mouse macrophages to induce IFN production.	^274^
			Mice; mouse BMDMs; human/murine alveolar macrophages	*Streptococcus pneumoniae*; bacterial DNA	IRF3/IFNAR knockout; STING siRNA	Cytoplasmic detection of bacterial DNA appears to induce STING-IRF3-mediated IFN production from *Streptococcus pneumoniae*-infected cells.	^275^
		Host apoptotic DNA	Mouse monocytes	*Streptococcus pneumoniae*	cGAS/STING knockout	Host apoptotic DNA is required for IFN-γ induction during *Streptococcus pneumonia* stimulation.	^856^
		mtDNA	Aged mice; mouse BMDMs	IAV	STING inhibitor H151; mtDNA depletion by ethidium bromide	Cytoplasmic mtDNA leakage triggers cGAS-STING activation in macrophages, worsening inflammation in viral pneumonia.	^297^
			A549 cells; human bronchial epithelial cells; 293 T cell	IAV	STING knockout; STING agonist diABZI; DNase I	TREX1 facilitates IAV replication by degrading cytosolic mtDNA and inhibiting cGAS-STING activation.	^300^
		Micronuclear DNA	RAW264.7 cells; RAW-Lucia-ISG cells; human mesenchymal stem cells	*Burkholderia pseudomallei*	cGAS/STING knockout	*Burkholderia pseudomallei* induces cell fusion and micronuclei formation that activates cGAS-STING-mediated autophagic cell death.	^190^
	cGAS/DDX41	mtDNA	Mice; A549 cells; MEFs; HEK293FT cells	IAV	cGAS/STING knockout; DDX41 siRNA; mtDNA depletion by ethidium bromide	IAV M2 protein induces mtDNA leakage into cytoplasm to drive cGAS/DDX41-dependent antiviral immunity. Nonstructural protein 1 of IAV impedes STING-dependent signaling via interference with DNA recognition.	^298^
	STING	mtDNA	Mice; A549 cells; MEFs	*Streptococcus pneumoniae*	STING knockout	*Streptococcus pneumoniae* hydrogen peroxide induces mitochondrial dysfunction and mtDNA leakage, triggering STING-mediated IFN response in alveolar epithelial cells.	^186^
			Mice; RAW264.7 cells; THP-1 cells; mouse primary peritoneal macrophages; MEFs	*Streptococcus pneumoniae*	STING knockout	*Streptococcus pneumoniae* main virulence factor pneumolysin induces mitochondrial oxidative damage and mtDNA leakage that triggers STING-mediated type I IFN response in macrophages.	^276^
	AIM2 inflammasome	cfDNA	Mice	IAV	AIM2 knockout; AAV-DNase I	Host DNA could be detected by AIM2 to induce protective immune response against IAV infection.	^303^
			Mice; human/mouse alveolar macrophages and type II cells	IAV	AIM2/ASC knockout; AIM2 siRNA	IAV activates AIM2 inflammasome, promoting DNA release and inflammation in the lung. AIM2 knockout reduces injury and improves survival without affecting viral clearance or immunity.	^304^
		Bacterial DNA	Mice; mouse BMDMs; human and murine alveolar macrophages	*Streptococcus pneumoniae*; bacterial DNA	AIM2 siRNA	Cytoplasmic detection of bacterial DNA appears to activate AIM2 inflammasome in *Streptococcus pneumoniae*-infected cells.	^275^
			Mice; mouse peritoneal macrophages	*Streptococcus pneumoniae*; bacterial DNA	AIM2/ASC/caspase-1 knockout; AIM2 siRNA; DNase I	Cytosolic bacterial DNA in macrophages activates AIM2 inflammasome that protects against *Streptococcus pneumoniae* infection.	^279^
		mtDNA; nDNA	J774A.1 macrophages; mouse BMDMs; HEK293FT cells	IAV	AIM2 knockout	IAV M2 and PB1-F2 proteins induce oxidization and cytosolic leakage of nDNA and mtDNA in mouse macrophages, initiating AIM2 inflammasome-dependent IL-1β production.	^234^
		NETs	Mice	LPS	AIM2 siRNA; DNase I; PAD inhibitor BB-Cl-amidine; caspase 1 inhibitor Ac-YVAD-cmk	NETs released during ARDS induce pyroptosis of alveolar macrophages in a DNA- and AIM2-dependent way.	^280^
	ZBP1-STING	Bacterial DNA	Mice; mouse primary nasal epithelial cells, BMDCs and BMDMs	*Streptococcus pneumoniae*; bacterial DNA	ZBP1/STING/IFNAR knockout; DNase	*Streptococcus pneumoniae* DNA upregulates IFN-β via the ZBP1-STING-TBK1-IRF3 axis, aiding bacterial clearance.	^281^
	ZBP1	Viral RNA	Mice; MEFs	IAV	ZBP1 knockout; ZBP1 sgRNA	ZBP1 recognizes IAV RNA and activates RIPK3-dependent apoptosis and necroptosis. ZBP1 knockout mice show impaired IAV control and increased susceptibility.	^353^
			Mice; MEFs; mouse neutrophils; A549 cells; HT-29 cells	IAV	ZBP1 knockout; RNase A; RNase III	ZBP1 senses defective viral genomes and Z-RNA generated during IAV replication, eliciting RIPK3-mediated MLKL activation in the nucleus and downstream necroptosis of IAV-infected cells.	^354^
	TLR3	Bacterial RNA	Human DCs	*Streptococcus pneumoniae*; bacterial RNA	TLR3/TRIF siRNA; TLR3/dsRNA complex inhibitor	*Streptococcus pneumoniae* RNA stimulates TLR3-TRIF pathway and subsequent IL-12p70 secretion from human DCs.	^283^
			U937 cells	*Pseudomonas aeruginosa* outer membrane vesicles	TLR3/dsRNA complex inhibitor	*Pseudomonas aeruginosa* outer membrane vesicles contain small RNAs that stimulate IFN production by macrophages via TLR3.	^284^
		Viral RNA	Mice; mouse BMDCs and lung epithelial cells	Synthetic viral RNA; *Staphylococcus aureus*	IFNLR1 knockout; poly(I:C); TLR7/8 agonist R848; RIG-I agonist triphosphate hairpin RNA	TLR3-induced IFN-λ production by lung DCs causes epithelial barrier damage, increasing susceptibility to bacterial superinfection in mice.	^314^
			BEAS-2B cells	IAV	-	TLR3 is the primary sensor that initiates proinflammatory response to IAV infection in human lung epithelial cells.	^308^
		Host RNA	Mice; mouse alveolar macrophages	*Klebsiella pneumoniae*	TLR3 knockout; TLR3/dsRNA complex inhibitor	In bacterial pneumonia, TLR3 detects dsRNA from necrotic cells. TLR3 deletion or inhibition enhances macrophage function and improves survival in *Klebsiella pneumoniae*-infected mice.	^282^
		Poly(I:C)	Mice	Poly(I:C)	TLR3/dsRNA complex inhibitor	Poly(I:C) induces pulmonary NET formation and ALI via TLR3.	^740^
			Obese mice	Poly(I:C)	-	Poly(I:C) upregulates pulmonary TLR3 expression and induces lung inflammation and injury in via TLR3 signaling.	^739^
	TLR3/7	Bacterial RNA	RAW 264.7 cells; mouse BMDMs	*Staphylococcus aureus* membrane vesicles	TLR3/TLR7/TRIF knockout; endosomal acidification inhibitor chloroquine	*Staphylococcus aureus* delivers bacterial RNA to macrophages via membrane vesicles and induce TLR-dependent IFN response.	^165^
	TLR3/MAVS	Poly(I:C)	Mice	Poly(I:C); *Streptococcus pneumoniae*; MRSA	TLR3/MAVS/IFNAR knockout; TLR7 agonist imiquimod and gardiquimod; anti-mouse IFNAR1 antibody	Poly(I:C)-pretreated mice show impaired clearance of *Streptococcus pneumoniae* and MRSA in lungs due to TLR3 and MAVS activation.	^741^
	TLR7	Viral RNA	Mice; pDCs; cDCs	IAV	TLR7/MyD88 knockout; endosomal acidification inhibitor chloroquine	Murine pDCs require TLR7-MyD88 activation for IFN-α production following the recognition of influenza genomic RNA.	^34^
	TLR8	Bacterial RNA	Human PBMCs, monocytes and macrophages	*Staphylococcus aureus*	TLR7/TLR8/IRF5 siRNA; TLR7/8 agonist CL075; RNAse A	*Staphylococcus aureus* RNA activates TLR8 in human monocytes, inducing IFN-β and IL-12 via IRF5 activation.	^288^
	RIG-I	Bacterial RNA	Mouse BMDMs	*Legionella pneumophila* RNA	RIG-I shRNA	*Legionella pneumophila* RNA upregulates type I IFN in macrophages via RIG-I.	^290^
		Viral RNA	Ferrets; A549 cells; HEK293T cells	IAV	RIG-I knockout	Mini viral RNAs from IAV, produced during dysregulated replication, are sensed by RIG-I to induce an IFN response.	^857^
			BEAS-2B cells	IAV	-	RIG-I, not MDA5, senses IAV and mediates proinflammatory and antiviral responses in human lung epithelial cells.	^308^
			Human NK cells	IAV	RIG-I crRNA; RIG-I agonist triphosphate RNA	IAV activates NF-κB and type I IFN response in human NK cells via RIG-I signaling.	^340^
		Host RNA	Human primary lung fibroblasts; MEFs; HEK293T cells	IAV	RIG-I/MDA5/cGAS/IFI16 knockout; RIG-I/*RNA5SP141* siRNA; poly(I:C)	Host RNA *RNA5SP141* translocates from the nucleus to cytoplasm, where it binds to RIG-I and induces type I IFN expression, thereby contributing to antiviral immunity against IAV.	^858^
		rRNA	Human PBMCs; HEK293T cells	IAV	RIG-I agonist 5’triphosphate RNA; RNase A	Self-RNA fragments from the 45S rRNA spacer region act as RIG-I ligands in IAV-infected HEK293 cells.	^859^
		miRNA	A549 cells	IAV	RIG-I siRNA; endosomal acidification inhibitor chloroquine	IAV-induced miRNA-136 triggers RIG-I signaling, boosting IL-6 and IFN-β expression in airway epithelial cells.	^334^
		Short duplex RNA	Mice; human lung airway chips and lung alveolus chips; A549 cells; THP-1 cells; HAP1 cells	IAV; SARS-CoV-2	RIG-I/MDA5/TLR3/TLR8/IRF3/IRF7 knockout	Self-assembling short duplex RNAs directly bind to RIG-I and potently induce type I and type III IFN responses against IAV and SARS-CoV-2 in differentiated human lung epithelial and endothelial cells in organ chips.	^420^
	RIG-I-MAVS	Viral RNA	Mouse primary lung fibroblasts and BMDMs	IAV	RIG-I/MAVS/IFNAR1/ZBP1/MyD88/TRIF knockout	RIG-I-MAVS signaling upon IAV RNA recognition is required by ZBP1 activation and downstream programmed cell death during IAV infection.	^307^
			A549 cells; HEK293T cells	IAV	RIG-I/MAVS knockout; MAVS siRNA	Nuclear RIG-I senses IAV RNA replication, triggering type I IFN and antiviral immunity via MAVS signaling.	^332^
			A549 cells	IAV; IAV RNA	MAVS knockout	Defective IAV RNA increases the surface presentation of human leukocyte antigen proteins in human lung epithelial cells via RIG-I-MAVS pathway.	^342^
	MDA5	Fungal RNA	Mice; mouse primary fibroblast	*Aspergillus fumigatus*; fungal RNA	MDA5/MAVS/IFNAR knockout	MDA-MAVS-dependent IFN response to *Aspergillus fumigatus* dsRNA triggers neutrophil antifungal activity in mouse lung infection.	^436^
	NOD2	Viral RNA	Mice; human primary bronchial epithelial cells; A549 cells; HEK293T cells; mouse BMDMs; MEFs	IAV; RSV; viral ssRNA	NOD2/MAVS knockout; NOD2/MAVS siRNA	NOD2 senses viral ssRNA genome and induces type I IFN response via MAVS-IRF3 axis in cells infected with IAV or RSV.	^348^
	DDX6/RIG-I	Viral RNA	A549 cells; HEK293T cells	IAV	DDX6/RIG-I siRNA	DDX6 co-senses IAV with RIG-I, binding viral RNA to enhance signaling and IFN-β induction.	^349^
	DHX16/RIG-I	Viral RNA	Mice; MEFs; A549 cells; Calu-3 cells	IAV; IAV RNA	RIG-I knockout; DHX16 PPMO knockdown; DHX16 siRNA; DXH16 overexpression	DHX16 binds IAV RNA and forms a complex with RIG-I to enhance IFN induction during influenza infection.	^350^
	IFI16/RIG-I	Viral RNA	Mice; A549 cells; HEK293T cells	IAV	IFI16/IFI204/RIG-I/IFNAR1 knockout	IFI16 promotes IAV-induced RIG-I activation by binding viral RNA and RIG-I. IFI16 also upregulates RIG-I expression by binding its promoter.	^352^
COVID-19	TLR9	cfDNA	Mouse primary proximal tubular cells	SARS-CoV-2	TLR9 inhibitor ODN 2088	Elevated cfDNA from COVID-19 plasma induces mitochondrial ROS overproduction in mouse renal cells, which could be inhibited by TLR9 inhibitor ODN 2088.	^238^
		mtDNA	Mice; Human umbilical vein endothelial cells	SARS-CoV-2	TLR9 knockout; TLR9 inhibitor ODN 2088	Mitochondrial impairment and mtDNA release induced by SARS-Cov-2 activates TLR9 signaling and inflammatory response in endothelial cells.	^376^
	cGAS-STING	mtDNA	Mice; human alveolar epithelial cells, lung microvascular endothelial cells and macrophages; THP-1 cells	SARS-CoV-2	cGAS knockout; STING shRNA; STING inhibitor H151; mtDNA depletion by 2′,3′-dideoxycytidine; VDAC1 inhibitor VBIT-4	SARS-CoV-2 induces mitochondrial dysfunction in pulmonary endothelial cells and activates cGAS–STING pathway via mtDNA leakage, leading to type I IFN production and cell death.	^361^
			A549 cells; Calu3 cells; Huh7 cells; HEK-293T cells	SARS-CoV-2	cGAS sgRNA	SARS-CoV-2 leads to the cytoplasmic accumulation of mtDNA, which triggers cGAS-STING to activate IFN-I signaling.	^368^
		nDNA	Mice; Calu-3 cells; HeLa-ACE2 cells; THP-1 cells; SARS-CoV-2 Spike expressing HEK293T cells	SARS-CoV-2	cGAS/STING knockout; STING agonist diABZI	SARS-CoV-2-induced cell fusion increases cytoplasmic chromatin that activates cGAS-STING-mediated antiviral response.	^367^
		Micronuclear DNA	Hela-ACE2 cells	SARS-CoV-2	-	SARS-CoV-2 infection induces syncytia formation and micronuclei production, which activate the cGAS-STING pathway.	^370^
		Micronuclear DNA	A549; HEK 293T cells; Vero E6 cells; HeLa-ACE2 cells; THP-1 cells	SARS-CoV-2	cGAS/STING knockout; cGAS/STING siRNA	Cell fusion and formation of micronuclei induced by SARS-CoV-2 spike protein contributes to cGAS-STING activation.	^371^
	STING	mtDNA; nDNA	Human primary airway epithelial cells and PBMCs; Vero E6 cells; human lung samples; patient BALF	SARS-CoV-2	STING inhibitor H151; caspase-1 inhibitor VX-765; IL-1Ra; benzonase	Genomic DNA and mtDNA from infected human airway epithelial cells are taken up by PBMCs, activating inflammasome and IL-1β production in SARS-CoV-2-primed PBMCs in a STING-dependent way. The released IL-1β in turn triggers IL-6 production from epithelial cells and PBMCs.	^369^
	TLR3	Viral RNA	Human PBMCs and T cells; J1.1 cells; Jurkat cells	COVID-19 patients-derived plasma exosomes	dsRNA/TLR3 complex inhibitor	COVID-19 plasma exosomes with viral dsRNA upregulate TLR3 and stimulate IL-6 and TNF-α production from PBMCs.	^374^
	TLR7/8	Viral RNA	Mice; human and mouse cDCs and pDCs; RAW264.7 cells; T cells	SARS-CoV-2 ssRNA	MyD88 knockout; TLR8/MyD88/TRIF/MAVS siRNA; TLR8 inhibitor CU-CPT9a; TLR7/8 agonist HIV-1–derived RNA40	SARS-CoV-2 ssRNA interacts with TLR7/8, activating MyD88 signaling, IFN production, DC maturation and type 1 T helper cell polarization in COVID-19 immunity.	^406^
			THP-1 cells; mouse TLR7 reporter HEK cells; human TLR7/8 reporter HEK cells	SARS-CoV-2 RNA	TLR7/8 agonist R848; TLR7 agonist loxoribine	Several ssRNA fragments from SARS-CoV-2 genome are identified as TLR7/8 ligands that stimulate cytokine release from human macrophages, including fragments s_6747, s_24432 and s_28225.	^860^
		miRNA	Human neutrophils and platelets; HL-60 cells	SARS-CoV-2 spike protein; COVID-19 patients-derived plasma EVs	TLR7/TLR8 siRNA; TLR8 inhibitor Cu-CPT9a	SARS-CoV-2 spike protein activates platelets, upregulating miRNA-21 and let-7b. Platelet-derived vesicles transfer miRNAs to neutrophils, activating TLR7/8, enhancing NET formation and upregulating IL-1β, TNF-α, and IL-8.	^404^
	TLR8	Viral RNA	Human neutrophils	SARS-CoV-2 ssRNA	TLR8 inhibitor CU-CPT9a; TLR7/8 agonist R848	The ssRNA of SARS-CoV-2 activates neutrophils to produce TNF-α, CXCL8 and IL-1Ra, and release NETs via TLR8.	^403^
			Human PBMC-derived macrophages	SARS-CoV-2 ssRNA	TLR7/TLR8/MyD88/NLRP3 siRNA; TLR8 agonist ssRNA40 and ssRNA41	GU-rich ssRNA derived from SARS-CoV-2 activates NLRP3 inflammasome and triggers TLR8-dependent IL-1β production from human macrophages.	^402^
	RIG-I	Viral RNA	A549 cells; Calu-3 cells; human pulmonary alveolar epithelial cells and bronchial epithelial cells	SARS-CoV-2	RIG-I/MAVS knockout; RIG-I/MDA5/TLR3 siRNA	RIG-I binds to the 3’ untranslated region of the SARS-CoV-2 RNA via helicase domains. This interaction directly blocks viral RNA-dependent RNA polymerase and abrogates SARS-CoV-2 replication in human lung cells in a MAVS- and IFN-independent manner.	^419^
			Hamster; human nasal epithelial cells; Calu-3 cells; MRC5 cells; THP-1 cells	SARS-CoV-2; SARS-CoV-2 RNA	RIG-I/MDA5 knockout; RIG-I/MDA5 siRNA; poly(I:C)	Small viral RNAs derived from SARS-CoV-2 bearing duplex structures and 5’-triphosphates activate the RIG-I signaling and contributes to delayed IFN response in COVID-19.	^418^
	RIG-I/MDA5	Viral RNA	A549 cells; THP-1 cells; HeLa cells; HEK293 cells	SARS-CoV-2; SARS-CoV-2 RNA	MDA5 knockout	Isolated SARS-CoV-2 RNA fragments prompt IFN responses via RIG-I and MAD5. 200-base RNA fragments preferentially signal through RIG-I but not MDA5.	^861^
	RIG-I/MDA5-MAVS	dsRNA	Human PBMCs; human monocyte-derived DCs; THP-1 cells	SARS-CoV-2; SARS-CoV-2–infected cells; RNA from SARS-CoV-2–infected cells; poly(I:C)	OAS1/OAS2/RNase L/RIG-I/MDA5/MAVS knockout; OAS1/OAS2/RNase L shRNA; RIG-I agonist 5’-triphosphate dsRNA	Human myeloid cells and monocytes with OAS-RNase L deficiencies display excessive inflammatory response to dsRNA and SARS-CoV-2 in a RIG-I/MDA5-MAVS-dependent manner.	^430^
	OAS-RNase L	dsRNA	Human PBMCs; human monocyte-derived DCs; THP-1 cells	SARS-CoV-2; SARS-CoV-2–infected cells; RNA from SARS-CoV-2–infected cells; poly(I:C)	OAS1/OAS2/RNase L/RIG-I/MDA5/MAVS knockout; OAS1/OAS2/RNase L shRNA; RIG-I agonist 5’-triphosphate dsRNA	OAS1, OAS2 or RNase L-deficient human myeloid cells, THP-1 cells and DCs show exaggerated inflammatory responses to dsRNA, SARS-CoV-2 and RNA from infected cells.	^430^
Sepsis	TLR9	Bacterial DNA	Mice; mouse BMDMs	CLP; LPS	Endosomal acidification inhibitor chloroquine	Serum bacterial DNA in sepsis partly comes from gut translocation. Bacterial DNA worsens disease and activates macrophages via TLR9.	^166^
		CpG-DNA	Mice	*Staphylococcus aureus*	TLR9 knockout	CpG-DNA enhances antibacterial immunity by protecting immune cells and boosting bacteria-reactive antibodies via TLR9.	^694^
			RAW264.7 cells; mouse primary peritoneal macrophages	*Staphylococcus aureus*	TLR9 knockout	CpG-DNA activates TLR9, promoting phagocytosis and autophagy in *Staphylococcus aureus*-stimulated macrophages for antibacterial immunity.	^696^
			Mice; mouse primary cardiomyocytes	CpG-DNA	TLR9 knockout	CpG-DNA induces cardiac inflammation and impairs cardiomyocyte contractility in a TLR9-dependent manner.	^508^
		Fungal DNA	Mice; mouse peritoneal macrophages, splenocytes and CD4 + T cells	*Candida albicans*; candidal DNA	Endosomal acidification inhibitor chloroquine	Candidal DNA pretreatment boosts cytokine release, T cell proliferation, inhibits kidney contamination and reduces mortality in *Candida albicans* infection, which could be reversed by chloroquine.	^454^
			Mice; mouse BMDMs and BMDCs	*Candida albicans*; candidal DNA	TLR9/MyD88/IFN-β knockout	*Candida albicans* and its DNA trigger IFN response in cDCs via TLR9 and MyD88.	^451^
			Mice; mouse BMDCs	*Cryptococcus neoformans*; cryptococcal DNA	TLR9/MyD88 knockout; endosomal acidification inhibitor chloroquine; TLR9 inhibitor ODN 2088 and ODN 2114; DNA methylase; DNase	Cryptococcal DNA activates BMDCs in a TLR9-dependent manner.	^452^
		mtDNA	Human PBMCs and neutrophils	LPS	Endosomal acidification inhibitor chloroquine	LPS stimulates monocytes to release mtDNA, which weakens neutrophil chemotaxis and suppresses immune antimicrobial function.	^862^
			Mice; human, chimpanzee and mouse RBCs	LPS; cecal slurry model; CpG-treated RBCs transfusion	TLR9 knockout	TLR9 on the surface of RBCs binds mtDNA during sepsis, causing morphological changes, promoting RBC clearance by splenic macrophages and leading to acute anemia.	^59^
			Mice	LPS; intraperitoneal administration of mtDNA	TLR9 inhibitor ODN 2088	LPS-challenged mice show increased circulating mtDNA, which induces ALI and systemic inflammation via TLR9.	^483^
			Mice; NR8383 cells	LPS; intratracheal administration of mtDNA	TLR9 siRNA	mtDNA promotes sepsis-induced lung injury by activating the TLR9-MyD88-NF-κB pathway in mouse lungs and alveolar macrophages.	^482^
			Mice; mouse/ human hepatocytes	LPS; CLP	TLR9 siRNA; TLR9 mutation	Sepsis-induced mitochondrial biogenesis in hepatocytes is dependent on mtDNA-TLR9-mediated autophagy.	^500^
			Mice	CLP	TLR9 knockout; DNase I	mtDNA activates TLR9 and contributes to cytokine production, tubular damage and AKI during sepsis.	^513^
		NETs	Mice; Caco2 cells	LPS; NET administration	TLR9 inhibitor ODN 2088	NETs increase in sepsis and trigger ERS via TLR9, leading to intestinal epithelial cell death and barrier dysfunction.	^517^
	cGAS-STING	mtDNA	Mouse BMDMs	LPS; NLRP3 activator ATP or nigericin	cGAS/STING knockout	Oxidized mtDNA activates the cGAS-STING pathway in LPS-primed mouse macrophages stimulated with NLRP3 activators.	^236^
			Mice; human/mouse lung microvascular endothelial cells	LPS; CLP	cGAS knockout; cGAS siRNA; STING agonist CMA; TBK1 overexpression	LPS activates gasdermin D that forms mitochondrial pores and induces mtDNA leakage into cytosol in endothelium. Released mtDNA activates the cGAS-STING pathway and impair vascular regeneration by suppressing YAP1 signaling in septic mice.	^457^
			Mice; mouse primary peritoneal macrophages	LPS	cGAS/STING knockout; cGAS siRNA; STING overexpression; diABZI	Cytosolic mtDNA activates cGAS-STING-NLRP3 axis that contributes to LPS-induced ALI.	^489^
			Mice; HK-2 cells	LPS	cGAS inhibitor RU.521; STING agonist DMXAA; mtDNA depletion by ethidium bromide	The cGAS-STING pathway senses cytosolic mtDNA and promotes NLPR3 inflammasome that contributes to sepsis-induced AKI.	^515^
	cGAS	Bacterial DNA	Mouse BMDMs	LPS; CLP; bacterial DNA	cGAS knockout	Bacterial DNA enhances LPS-induced proinflammatory response in mouse macrophages partly via cGAS-dependent manner.	^455^
			Mice; mouse BMDMs	HSV-1; vesicular stomatitis virus	cGAS/STING/IFN-β/IFNAR/MyD88 knockout	Gut microbiota vesicles with bacterial DNA enter circulation, priming antiviral immunity and enhancing HSV-1 and vesicular stomatitis virus clearance via the cGAS-STING-IFN axis.	^456^
		Cytosolic DNA	Mice; mouse isolated thoracic aortas and mesenteric arteries; rat aortic smooth muscle cells and primary aortic endothelial cells	LPS	cGAS knockout; cGAS agonist G3-YSD; STING siRNA; TBK1 siRNA	cGAS in vascular endothelial cells detects cytosolic DNA and generates cGAMP that is then exported to neighboring VSMCs. PKG in VSMCs is then activated independently of STING, which triggers vessel relaxation and contributes to hypotension and tissue hypoperfusion during sepsis.	^458^
		mtDNA	Mice; mouse BMDMs	LPS; CLP	cGAS knockout	mtDNA-activated cGAS triggers pro-inflammatory effects in LPS-stimulated macrophages, contributing to septic hyper-inflammation in wild-type mice compared to cGAS knockout mice.	^455^
			Mice; mouse peritoneal macrophages, BMDMs and alveolar macrophages	LPS	cGAS knockout	Upon LPS stimulation, cGAS mediates macrophage polarization to the inflammatory phenotype via the mtDNA-mTORC1 axis.	^718^
	STING	mtDNA	Mice; mouse BMDMs	LPS; CLP	STING knockout	In septic mice, mtDNA accumulates in splenic DCs, inducing immunoparalysis via STING signaling.	^466^
			Mice; mouse BMDMs	CLP	STING knockout; STING agonist DMXAA; DNase I	Overactivation of mtDNA-STING signaling in the gut causes hyper-inflammation, epithelial cell apoptosis, barrier dysfunction and lethal sepsis.	^518^
			Mice; mouse BMDMs; RAW264.7 cells	LPS; CLP; intraperitoneal administration of mtDNA	STING knockout; TBK1 siRNA	STING is activated by mtDNA, impedes lysosomal acidification and disturbs autophagic flux in macrophages via IFN, aggravating sepsis-induced ALI.	^488^
			Mice; mouse primary Kupffer cells and hepatocytes	LPS	STING knockout; STING agonist DMXAA; DNase I	Mitochondrial fission and VDAC1 pore formation release mtDNA in LPS-treated Kupffer cells, activating STING signaling via Drp1, leading to hepatocyte death, liver injury and systemic inflammation.	^499^
		NETs	Mice; human umbilical vein endothelial cells	CLP; NET administration	STING inhibitor H151; DNase I	NETs activate STING pathway in endothelial cells via TLR2 and cause inflammation and coagulation in the lungs, contributing to the poor outcome of sepsis-induced ALI.	^493^
			Mice	LPS	STING inhibitor H151; DNase I	NETs worsen lung injury by activating the cGAS-STING pathway in LPS-induced ALI.	^494^
	RNA‐polymerase III/RIG-I	Bacterial DNA	Mice; mouse BMDMs	*Listeria monocytogenes*; bacterial DNA	RIG-I/MDA5/MyD88/TRIF/ASC knockout	*Listeria monocytogenes* DNA transcription by RNA polymerase III activates RIG-I and induces IFN.	^863^
	TLR7	miRNA	Mice; mouse BMDMs; human pulmonary artery and microvascular epithelial cells	CLP; miRNA-146-5p administration	TLR7/miRNA-146 knockout	TLR7 in macrophages senses extracellular miRNA-146a-5p and triggers pulmonary inflammation and endothelial barrier dysfunction via TNF-α, contributing to sepsis-induced ARDS.	^496^
			Mice; mouse BMDMs	CLP; intraperitoneal fecal slurry	TLR7/TLR3/MyD88/TRIF knockout; TLR7 agonist imiquimod	TLR7 knockout preserves clotting and reduces procoagulant response in CLP or fecal slurry-challenged mice. Exogenous miRNA-146a stimulates tissue factor production by macrophages via TLR7.	^475^
			Mice; mouse BMDMs	CLP	TLR7/MyD88/TLR3/TRIF knockout; RNase; miRNA inhibitor; miRNA-146a mimics	Septic mice EVs contain miRNAs (miRNA-34a, miRNA-122, miRNA-146a) that induce proinflammatory cytokine production by macrophages via TLR7-MyD88 pathway.	^473^
			Mice; mouse primary microglia and astrocytes	CLP	TLR7/miRNA-146 knockout; TLR7 agonist imiquimod	In sepsis, plasma miRNA-146-5a activates TLR7, recruiting monocytes and neutrophils, and contributing to blood-brain barrier disruption and encephalopathy.	^505^
			Mice; mouse BMDMs	CLP; miRNA-146a-5p administration	miRNA-146a knockout; TLR7 agonist imiquimod	Exogenous miRNA-146a-5p activates immunity via UU-motif interaction with TLR7, while miRNA-146a knockout protects mice from CLP-induced sepsis.	^827^
			Mice; RAW 264.7 cells; mouse primary microglia, astrocytes and neurons	CLP; EV administration	TLR7/MyD88 knockout; miRNA inhibitors against miRNA-146a, -122, -34a, and -145a	Plasma EVs from septic mice could be taken up by brain cells, inducing microglial activation via cargo miRNAs and TLR7 as well as cerebral immune response via MyD88 signaling.	^506^
		Bacterial RNA	Mice; HEK293 cells	*Listeria monocytogenes*	RIG-I siRNA; RNase A	RIG-I detects a *Listeria monocytogenes* RNA-Zea complex, promoting IFN response.	^864^
	RIG-I	Host RNA	Human primary normal lung fibroblasts; MEFs; HEK293T cells	HSV-1	RIG-I/MDA5/cGAS/IFI16 knockout; RIG-I/*RNA5SP141* siRNA; poly(I:C)	In infected cells, host RNA *RNA5SP141* translocates from the nucleus to cytoplasm while its interacting proteins are downregulated. *RNA5SP141* subsequently binds to RIG-I and induces antiviral immunity against HSV-1.	^858^
	RIG-I/MDA5	Bacterial RNA	Mice; mouse BMDMs	*Listeria monocytogenes*; bacterial RNA	RIG-I/MDA5/MyD88/TRIF/ASC knockout	Intracellular *Listeria monocytogenes* RNA in macrophages stimulates IFN and inflammasome signaling via RIG-I and MDA5.	^863^
Trauma	TLR9	mtDNA	Human PMNs	mtDNA; mitochondrial DAMPs	-	Mitochondrial DAMPs, including mtDNA and formyl peptides, activate PMNs via CpG/TLR9 and promote Ca2+ flux.	^522^
		NETs	Mouse traumatic brain injury model; HT-22 cells	-	TLR 9 inhibitor ODN 2088; PAD inhibitor Cl-amidine; DNase I	The inhibition of NETs formation, degradation of NETs or TLR9 inhibition reduces ERS and neuronal apoptosis, improving neurological outcome.	^865^
	AIM2 inflammasome	cfDNA	Mice burn and stroke model; human burn and stroke samples; mouse BMDMs and T cells	-	AIM2/caspase 1 knockout; caspase-1 inhibitor VX-765; anti-IL-1β antibody	cfDNA from burns or strokes activates the AIM2 inflammasome in myeloid cells, triggering IL-1β-induced FasL expression, which induces T cell apoptosis and infection susceptibility.	^542^
	TLR3	Host dsRNA	Mouse lung contusion; mouse alveolar macrophages	-	TLR3/TRIF knockout; anti-TLR3 antibody; TLR3/dsRNA complex inhibitor	Injured cell-derived dsRNA drives acute inflammation and progressive lung injury after lung contusion via TLR3.	^549^
MI	cGAS-STING	Cytosolic DNA	Mouse LCA ligation; mouse BMDMs; THP-1 cells	-	cGAS knockout; cGAS mutation	The cGAS-STING pathway senses DNA from necrotic heart tissue, hindering repair. cGAS deficiency promotes M2-like macrophages, limits remodeling, preserves function and improves MI outcomes.	^563^
	AIM2 inflammasome	mtDNA	Mouse LCA ligation; mouse ventricular myocytes and cardiac macrophages	-	-	Impaired mitophagy in type 2 diabetes leads to cytosolic mtDNA accumulation and AIM2 inflammasome activation in cardiomyocytes and macrophages, causing cell death and increased ischemic heart failure susceptibility.	^568^
	IFI16/IFI204	mtDNA	Mouse LCA ligation; cell hypoxia and nutrient deprivation; AC16 cells	-	IFI16/IFI204 shRNA; VDAC inhibitor VBIT-4	IFI16/IFI204 recognizes mtDNA leakage and activates inflammasome formation, while shRNA targeting IFI16/IFI204 suppresses inflammasome activation, myocardial apoptosis, cardiac dysfunction and remodeling following MI.	^571^
	ZBP1	mtDNA	Mouse LCA ligation; mouse myocytes	mtDNA	ZBP1 knockout; ZBP1/TLR9 siRNA; ZBP1 overexpression	ZBP1 detects mtDNA and protects against MI by suppressing TLR9-mediated inflammation in cardiomyocytes and alleviating cardiac dysfunction.	^572^
Myocardial IR	TLR9	cfDNA	Mouse LCA ligation and reperfusion	-	TLR9 knockout; DNase I	Ischemic cardiomyocyte-released cfDNA and HMGB1 promote systemic inflammation, activate splenic leukocytes and worsen myocardial IRI via the RAGE-TLR9 pathway.	^557^
		mtDNA	Langendorff-perfused isolated mouse heart	-	TLR9 knockout; DNase I	mtDNA released from necrotic cardiomyocytes in myocardial IRI activates TLR9, causing inflammation and injury. TLR9 deficiency or mtDNA digestion improves cardiac function and reduces necrosis and inflammation.	^558^
			Rat LCA ligation and reperfusion; H9c2s cells	-	Endosomal acidification inhibitor chloroquine	Circulating mtDNA increases infarct size and aggravates cardiac IRI via TLR9-p38 MAPK pathway.	^559^
			Mouse LCA ligation and reperfusion; mouse primary peritoneal macrophages	-	TLR9 inhibitor ODN 2088; DNase I	The NLRP3 inflammasome in splenic monocytes is activated during myocardial IRI, triggering systemic inflammation and worsening cardiac injury via mtDNA-TLR9 signaling.	^561^
	cGAS-STING	cfDNA	Mouse LCA ligation and reperfusion; mouse splenic leukocytes	-	cGAS inhibitor RU.521; anti-STING antibody; anti-IRF3 antibody; IFNAR1 knockout; anti-IFNAR1 monoclonal antibody	Cardiogenic cfDNA together with HMGB1 exacerbates myocardial IRI by activating cGAS-STING-IRF3 axis and type I IFN response in splenic pDCs.	^564^
		mtDNA	Mouse LCA ligation and reperfusion; diabetic mice; H9C2 cells	-	STING inhibitor H151; mtDNA depletion by ethidium bromide	The mtDNA cytosolic leakage caused by impaired mitochondrial fusion could activate cGAS-STING pathway that facilitates the pathogenesis of diabetic myocardial IRI.	^566^
			Mouse LCA ligation and reperfusion; mouse primary cardiac myocytes and fibroblasts	-	cGAS/STING siRNA	In ischemic and peripheral areas of mouse IR hearts, small EVs derived from cardiomyocytes could be internalized by fibroblasts and deliver mtDNA that activates cGAS-STING pathway to promote fibroblast proliferation and cardiac fibrosis.	^567^
	TLR7	Host RNA	Mouse LCA ligation and reperfusion; cell hypoxia; rat/mouse primary neonatal cardiomyocytes	-	TLR3/TRIF/IFNAR knockout	RNA released from necrotic macrophages and cardiomyocytes induces myocardial inflammation, cardiomyocyte apoptosis and cardiac dysfunction after myocardial IR partly via TLR3-TRIF pathway.	^574^
Hepatic IR	TLR9	cfDNA	Mouse segmental hepatic warm IR; mouse primary hepatocytes, neutrophils and liver non-parenchymal cells	-	TLR9 knockout; TLR9 inhibitor inhibitory CpG; DNase I	DNA released from necrotic hepatocytes increases liver non-parenchymal cells and induces cytokine release from neutrophils via TLR9. Inhibiting TLR9 protects against hepatic IRI.	^584^
	cGAS-STING	cfDNA	Mouse partial hepatic warm IR	-	-	Hepatocyte oxidative DNA damage induced by IR triggers cGAS-STING activation in macrophages.	^237^
		mtDNA	Mouse partial hepatic warm IR; Hep3B cells; mouse hepatocytes and liver macrophages	-	STING knockout; cGAS inhibitor G150; STING inhibitor H151; mPTP inhibitor cyclosporin A; VDAC inhibitor VBIT-4; BAX/BAK inhibitor MSN-125	The release of mtDNA exacerbates hepatic IRI via cGAS-STING pathway. Inhibiting mtDNA release offers hepatoprotection in liver IRI.	^586^
	STING	mtDNA	Mouse partial hepatic warm IR; hepatocyte HR; mouse BMDMs, primary liver macrophages and hepatocytes	-	STING knockout; STING inhibitors C-178 and H151	Hepatocyte mtDNA released during hepatic IR could activate macrophage STING signaling and increase inflammatory cytokine production. Inhibiting STING protects against hepatic IRI.	^587^
			Mouse partial hepatic warm IR; hepatocyte HR; mouse primary hepatocytes, Kupffer cells and BMDMs	-	STING inhibitor C-176; STING siRNA	The increased mtDNA activates NLRP3 signaling in macrophages from aged liver after ischemia/reperfusion in a STING-dependent manner.	^589^
	AIM2 inflammasome	cfDNA; cytosolic DNA	Mouse partial hepatic IR	-	AIM2 siRNA	Host DNA can activate the AIM2 inflammasome in the liver, driving excessive inflammation after IR.	^585^
Renal IR	TLR9	mtDNA	Mouse right nephrectomy and left renal IR	-	TLR9 knockout; TLR9 agonist ODN 1668	Intestinal TLR9 protects against ischemic kidney injury and remote organ dysfunction by inhibiting IL-17A release from Paneth cells. MtDNA from dying renal cells activates TLR9 in remote organs.	^592^
	cGAS-STING	mtDNA	Mouse left renal IR; HK-2 cells	-	-	RIPK3-induced mtDNA release activates the cGAS-STING-NF-κB pathway, increasing inflammation and worsening renal IRI.	^594^
			Moue bilateral renal IR; mouse proximal tubular cells	-	-	The leakage of mtDNA triggered by Drp1-Fis1 interaction induces cGAS-STING activation and promotes inflammation that exacerbates renal IRI.	^595^
			Moue bilateral renal IR; cell HR; mouse renal proximal tubular epithelial cells and primary renal tubular cells	-	cGAS knockout; cGAS siRNA	The release of mtDNA induced by PGAM5-mediated BAX dephosphorylation activates cGAS-STING pathway and promotes inflammation in renal IRI.	^596^

*AAV* adeno-associated virus, *AIM2* absent in melanoma 2, *AKI* acute kidney injury, *ALI* acute lung injury, *ARDS* acute respiratory distress syndrome, *ASC* apoptosis-associated speck-like protein containing a caspase recruitment domain, *ATP* adenosine triphosphate, *BAK* Bcl-2 homologs antagonist/killer, *BALF* bronchoalveolar lavage fluid, *BAX* Bcl-2-associated protein X, *BMDCs* bone marrow-derived dendritic cells, *BMDMs* bone marrow-derived macrophages, *cDCs* conventional dendritic cells, *cfDNA* cell-free DNA, *cGAMP* 2´3´-cyclic-GMP-AMP, *cGAS* cyclic GMP-AMP synthase, *CLP* cecal ligation and puncture, *CMA* 10-carboxymethyl-9-acridanone, *COVID-19* coronavirus disease 2019, *CpG* cytosine-phosphate-guanosine, *crRNA* CRISPR RNA*, CXCL8* CXC motif chemokine ligand 8, *DAMP* damage-associated molecular pattern, *DCs* dendritic cells, *DDX* DEAD-box helicase, *DHX* DExH-box helicase, *diABZI* dimeric amidobenzimidazole, *DMXAA* 5,6-Dimethylxanthenone-4-acetic acid, *DNA* deoxyribonucleic acid, *DNase* deoxyribonuclease, *Drp1* dynamin-related protein 1, *dsRNA* double-stranded RNA, *E. coli Escherichia coli*, *ERS* endoplasmic reticulum stress, *EVs* extracellular vesicles, *FasL* Fas ligand, *Fis1* fission 1, *G3-YSD* G3-ended Y-form Short DNA, *HMGB1* high mobility group box 1, *HR* hypoxia/reoxygenation, *HSV-1* herpes simplex virus 1, *IAV* influenza virus A, *IFI* interferon-γ-inducible protein, *IFN* interferon, *IFNAR* interferon alpha/beta receptor, *IFNLR1* interferon-α/β receptor 1, *IL* interleukin, *IL-1Ra* interleukin-1 receptor antagonist, *IR* ischemia/reperfusion, *IRF* interferon regulatory factor, *IRI* ischemia/reperfusion injury, *LCA* left coronary artery, *LPS* lipopolysaccharides, *MAPK* mitogen-activated protein kinase, *MAVS* mitochondrial antiviral signaling protein, *MDA5* melanoma differentiation-associated protein 5, *MEFs* mouse embryonic fibroblasts, *MI* myocardial infarction, *miRNA* microRNA, *MLKL* mixed lineage kinase domain like pseudokinase, *mPTP* mitochondrial permeability transition pore, *MRSA* methicillin-resistant *Staphylococcus aureus*, *mtDNA* mitochondrial DNA, *mTORC1* mammalian target of rapamycin complex 1, *MyD88* myeloid differentiation primary response 88, *nDNA* nuclear DNA, *NETs* neutrophil extracellular traps, *NF-κB* nuclear factor kappa-light-chain-enhancer of activated B cells, *NLRP3* NLR family pyrin domain containing 3, *NOD2* nucleotide-binding oligomerization domain-containing protein 2, *OAS* 2′-5′ oligoadenylate synthase, *ODN* oligodeoxynucleotides, *PAD* protein-arginine deiminase, *PBMCs* peripheral blood mononuclear cells, *pDCs* plasmacytoid dendritic cells, *PGAM5* phosphoglycerate mutase 5, *PKG* cGMP-dependent protein kinase, *PMNs* polymorphonuclear neutrophils, *poly(I:C)* polyinosine:polycytidylic acid, *PPMO* peptide-conjugated phosphorodiamidate morpholino oligomers, *RAGE* receptor for advanced glycation end products, *RBCs* red blood cells, *Ref*. reference, *RIG-I* retinoic acid-inducible gene I, *RIPK3* receptor-interacting protein kinase 3, *RNA* ribonucleic acid, *RNA5SP141* 5S ribosomal RNA pseudogene 141, *RNase* ribonuclease, *ROS* reactive oxygen species, *rRNA* ribosomal RNA, *RSV* respiratory syncytial virus, *SARS-CoV-2* severe acute respiratory syndrome coronavirus 2, *sgRNA* single guide RNA, *shRNA* short hairpin RNA, *siRNA* small interfering RNA, *ssRNA* single-stranded RNA, *STING* stimulator of IFN genes, *TBK1* TANK-binding kinase 1, *TLR* Toll-like receptor, *TREX1* three prime repair exonuclease 1, *TRIF* TIR (Toll/interleukin-1 receptor) domain-containing adaptor protein inducing interferon beta, *VDAC* voltage-dependent anion channel, *VSMCs* vascular smooth muscle cells, *YAP1* yes-associated protein 1, *ZBP1* Z-DNA binding protein 1

### Bacterial pneumonia-related ARDS

#### DNA sensing

Bacteria responsible for ARDS include *Staphylococcus aureus*, *Streptococcus pneumoniae, Pseudomonas aeruginosa* and *Legionella pneumophila*.^263,264^ Bacterial DNA causes lung and systemic inflammation, increases endothelial permeability and induces ALI presumably via TLR9 that is upregulated in pDCs from patients with pneumonia.^265–268^ *Staphylococcus aureus* also secrete bacterial DNA via membrane vesicles that could be sensed by TLR9 in macrophages and activate IFN response.^165^ Besides, in methicillin-resistant *Staphylococcus aureus* (MRSA)-induced pneumonia, self-DNA detection by TLR9 drives an inflammatory response, decreases bacterial load and inhibits lung consolidation.^269^ In line with this, TLR9 is requisite for antibacterial response in *Legionella pneumophila*-infected mice.^270^ Whereas, TLR9 exacerbates respiratory infection of *Pseudomonas aeruginosa* via impairing bacterial clearance ability of lung macrophages and reducing production of proinflammatory cytokines and nitric oxide.^271^

Intracellular infection directly exposes microbial NA to DNA sensors. For example, *Legionella pneumophila* releases bacterial DNA into cytosol and activates cGAS-STING to induce IFN response.^272^ This signaling is required for the clearance of *Legionella pneumophila* by macrophages in mouse lungs.^273^ Intracellular infection of *Burkholderia pseudomallei* induces cell fusion, micronuclei formation and DNA damage that activates cGAS-STING-mediated autophagic cell death.^190^ cGAS-STING pathway also senses *Streptococcus pneumoniae* DNA.^274,275^ In addition, STING-mediated type I IFN response is triggered by *Streptococcus pneumoniae* hydrogen peroxide and main virulence factor pneumolysin via mitochondrial oxidative damage and mtDNA release.^186,276^ However, a previous study conversely argued that cGAS-STING pathway has limited role in antibacterial immunity against *Streptococcus pneumoniae* in human and mice, as STING deficiency does not influence bacterial load and production of IL-1β, IL-6 and tumor necrosis factor α (TNF-α).^274^ This assumption raises a question of the additional function of cGAS-STING signaling during microbial infection. One possible answer is that STING suppresses cell death in macrophages to facilitate bacterial control, while STING deficiency enhances macrophage necroptosis, microbial reproduction and mortality in *Staphylococcus aureus* pneumonia.^277^

The production of inflammasome-caspase-1-dependent IL-1β and IL-18 is critical for bacterial pneumonia-related ARDS.^278^ *Streptococcus pneumoniae* DNA activates the AIM2 inflammasome in macrophages,^275^ which is crucial for innate immune response to the infection.^279^ Deletion of AIM2 dampens caspase-1 activation in *Streptococcus pneumoniae*-stimulated macrophages.^279^ Myeloperoxidase-DNA complex (marker of NETs) and caspase-1 levels in human bronchial aspirates are correlated with severity of gram-negative bacterial pneumonia-induced ARDS and increased in non-survivors compared with survivors.^280^ Application of AIM2 siRNA confirmed that NETs released during ARDS induce pyroptosis of alveolar macrophages in a DNA- and AIM2-dependent way.^280^ Besides, *Streptococcus pneumoniae* DNA upregulates IFN-β expression through a ZBP1-STING-TBK1-IRF3 axis that contributes to bacterial clearance.^281^

#### RNA sensing

RNA-sensing TLRs are activated during pulmonary bacterial infection. Lung samples from patients with *Klebsiella pneumoniae* exhibit higher expression of TLR3 compared with normal lung samples.^282^ TLR3 detects dsRNA released from necrotic cells during bacterial pneumonia.^282^ Furthermore, *Streptococcus pneumoniae* RNA stimulates TLR3-TRIF pathway and subsequent IL-12p70 secretion from human DCs.^283^ Outer membrane vesicles produced by *Pseudomonas aeruginosa* contain small RNAs with hairpin structure, which stimulate IFN production from macrophages via TLR3.^284^ Furthermore, TLR7 expression is upregulated during *Pseudomonas aeruginosa* pneumonia in mice,^285^ while TLR8 is the main sensor of pyogenic bacteria including *Staphylococcus aureus*, *Streptococcus agalactiae* and *Streptococcus pneumonia* in human monocytes and macrophages.^286–288^ TLR8 activated by *Staphylococcus aureus* RNA induces IFN-β and IL-12 production in human monocytes and monocyte-derived macrophages via TAK1-IKKβ-IRF5 pathway.^287^

The role of RLR in bacterial pneumonia remains unclear. Microarray analysis demonstrated enhanced RIG-I signaling in alveolar macrophages infected with *Streptococcus pneumoniae*, *Klebsiella pneumoniae* or *Staphylococcus aureus*.^289^ RNA from *Legionella pneumophila* upregulates type I IFN expression in macrophages via RIG-I.^290^ However, in *Legionella pneumophila*-infected human lung epithelial cells, the IFN-β induction is mediated by IRF3 and MAVS but not RIG-I or MDA5.^291^

### Viral pneumonia-related ARDS

#### TLR9

IAV is the predominant viral pathogen causing ARDS.^292^ TLR9 expression in DCs and monocytes is upregulated in IAV patients, which is associated with increased inflammatory cytokines in plasma and decreased viral burden in respiratory samples.^293^ IAV also increases TLR9 expression in non-infected macrophages from mice.^294^ IAV-induced mtDNA release in lung structural cells activates TLR9-mediated inflammatory and facilitates secondary bacterial infection.^294,295^ A review even argued that TLR9 may be inactivated in influenza-associated ARDS.^296^ Therefore, it is not solid that DNA activates TLR9 to regulate viral pneumonia-related ARDS.

#### cGAS-STING

mtDNA release into cytoplasm of macrophages triggers aberrant cGAS-STING activation and aggravates inflammatory response in viral pneumonia.^297^ IAV M2 protein induces mtDNA release into cytoplasm in a MAVS-dependent manner, driving cGAS/DDX41-dependent antiviral immunity.^298^ STING-dependent immune response restrains IAV replication, which amplifies from infected cells to neighboring cells via gap junctions.^298^ Interestingly, the production of IFN to control IAV requires a STING-dependent and cGAS-independent pathway.^299^ In contrast, nonstructural protein 1 of IAV impedes STING-dependent signaling via interference with DNA recognition.^298^ IAV also directly interacts with STING via hemagglutinin fusion peptide to inhibit IFN generation.^299^ A genome-wide screen identified three prime repair exonuclease 1 as pro-viral factor that facilitates IAV replication by degrading cytosolic mtDNA and inhibiting cGAS-STING activation.^300^ Besides, overactive STING may lead to inflammation and ARDS. Airway exposure to STING agonist dimeric amidobenzimidazole (diABZI) induces self-DNA release, NET formation, the upregulation of various DNA sensors (including cGAS, AIM2, DDX41 and IFI204) and STING-dependent type I IFN response in lung, together with TLR9-mediated neutrophilic inflammation, ZBP1-mediated PANoptosis and ARDS.^301^

#### AIM2 inflammasome

The role of the AIM2 inflammasome in influenza is recently reviewed by Australian researchers.^302^ Host DNA could be detected by AIM2 to induce protective immune response against IAV infection.^303^ IAV activates the AIM2 inflammasome, promotes host DNA release and inflammation in the lung, while AIM2 knockout reduces lung injury and improves survival without compromising viral clearance and adaptive immunity.^304^ IAV M2 and PB1-F2 proteins induce oxidization and cytosolic release of nDNA and mtDNA in mouse macrophages, initiating AIM2 inflammasome-dependent IL-1β production.^234^

#### Other DNA sensors

ZBP1 expression is upregulated in the lung during IAV infection, particularly in mouse alveolar epithelial cells, lung fibroblasts, bone marrow derived macrophages and human peripheral blood mononuclear cells (PBMCs).^130,305^ ZBP1 activates the NLRP3 inflammasome and programmed cell death in IAV-infected cells, while ZBP1 knockout reduces inflammation and epithelial damage, protecting mice from IAV infection.^306^ However, ZBP1 recognizes viral proteins and its activation requires RIG-I signaling, while its potential role in DNA sensing during IAV infection remains unknown.^306,307^

#### TLR3

TLR3 is the primary sensor that initiates pro-inflammatory response to influenza infection.^308^ Its upregulation in murine lungs, human lung epithelial cells and DCs post IAV challenge has been confirmed by many studies.^293,309–311^ In detail, TLR3 is responsible for IFN-λ induction and NF-κB activation in human airway epithelial cells following IAV stimulation.^310,312^ IAV-induced TLR3 activation in airway epithelial cells promotes ciliary activity and cilia-driven flow via ATP autocrine, facilitating the mucociliary clearance of invading viruses.^313^ The increased TLR3 expression in DCs from influenza patients negatively correlates with viral loads in respiratory tracts of patients.^293^ In murine pDCs, TLR system including TLR3 rather than RIG-I signaling is preferentially activated to produce IFN-α when exposed to RNA viruses.^42^ Interestingly, IAV-induced upregulation of TLR3 in human DCs sensitizes the cells to pneumococcal RNA, suggesting the possible involvement of TLR3 in secondary infection following IAV.^283^ TLR3-downstream IFN-λ production by lung DCs in response to viral RNA further acts on lung epithelial cells and induces barrier damage, leading to susceptibility to lethal bacterial superinfection in mice.^314^ In addition, murine mastocytoma cells are another responder to IAV, producing IL-6, IFN-γ, TNF-α and CCL-2 via TLR3.^315^

#### TLR7/8

TLR7 is required by immune cells in response to IAV. Type I IFN production by murine pDCs upon recognition of influenza genomic RNA is dependent on the TLR7-MyD88 pathway.^34,35,316^ In macrophages, TLR7 activation promotes NADPH oxidase 2-dependent oxidative burst following IAV infection.^317^ TLR7 is also indispensable for murine neutrophil activation and TNF-α production upon IAV recognition.^318^ Additionally, IAV induces pulmonary IFN-γ production, cluster of differentiation (CD) 69 expression in lung NK cells and the degranulation of splenic NK cells in mice via TLR7.^319^ However, TLR7 may facilitate viral replication upon sublethal IAV challenge by recruiting viral target cells to airway.^320^ Besides, IAV-activated TLR7 in human platelets leads to C3 secretion, followed by neutrophil-DNA release and NETosis.^321^

TLR8 expression is significantly increased in monocytes and DCs from patients with influenza, presenting negative and positive correlation with respiratory tract viral loads and plasma levels of IL-8, respectively.^293^ Of note, TLR7 and TLR8 activate distinct signaling pathways that perform different functions in human monocytes during RNA virus infection. Human monocytes produce T helper 17 cell polarizing cytokines via TLR7, and type I IFN and T helper 1 polarizing cytokines via TLR8.^322^ TLR7 deletion impairs CD4^+^ T cell response during IAV infection but does not disrupt CD8^+^ T cell response.^323^ Furthermore, TLR7-MyD88 pathway is proposed to govern the protective adaptive immune responses to IAV.^324^ Early research found that TLR7-MyD88 supports B cell proliferation, IgG1 production and anti-influenza antibody isotype switching.^325^ During IAV infection in aged mice, a Fas^+^GL7^-^ effector B cell population dominates the immune response and contributes to faster recovery, higher antibody titers and potential virus neutralization, which is derived from IgD^+^ age-associated B cell progenitors in a T cell-independent but TLR7-dependet fashion.^326^

#### RIG-I

RIG-I is the dominant RNA sensor for IFN production in response to RNA viruses.^40,307^ During infection of IAV and human respiratory syncytial virus (RSV), RIG-I rather than MDA5 is considered indispensable for antiviral immunity.^308,327^ Impaired RIG-I signaling in monocytes from the elderly is believed to contribute to reduced antiviral resistance to IAV.^328^ In IAV-infected ferrets, bronchioles are the first source of IFN-α and pro-inflammatory cytokines upon RIG-I activation.^329^ RIG-I is as essential as TLR3 in optimizing IFN production by human lung epithelial cells against IAV, presumably via MAVS-IRF3 axis.^330,331^

Intriguingly, RIG-I has recently been found in the nucleus, although it was previously known as a cytoplasmic RNA sensor. In A549 cells, nuclear RIG-I detects IAV RNA replication, triggering type I IFN via MAVS signaling and promoting antiviral immunity.^332^ Exosomes released from IAV-infected A549 cells could suppress the expression of RIG-I in uninfected A549 cells, indicating a possible mechanism of effective IAV replicaiton.^333^ Endogenous miRNA-136 upregulated by IAV infection also triggers RIG-I signaling and enhances L-6 and IFN-β expression in airway epithelial cells.^334^ Moreover, experiments with RIG-I- or MDA5-deficient cells showed that the IRF3 activation in influenza B virus-infected mouse embryonic fibroblasts requires RIG-I but not MDA5.^335^ RIG-I deficiency impairs viral clearance and increases mortality in IAV-infected mice,^323^ but some studies reported that deleting RIG-I or MAVS doesn’t reduce survival or innate cytokine responses.^336^ In sublethal IAV infection, RIG-I can enhance viral replication by recruiting target cells to the airway.^320^ Apart from influenza viruses, RSV also increases RIG-I expression in human epithelial cells, leading to the upregulation of NOD-like receptor family CARD domain containing 5 and major histocompatibility class I.^337^

As for immune cells, the mRNA levels of RIG-I are remarkably upregulated in circulating leukocytes from influenza patients.^293,338^ RIG-I-MAVS pathway is essential for migratory DC activation, viral antigen presentation and polyfunctional T cell responses against IAV, protecting epithelial cells and hematopoietic cells via downstream IFN production.^323^ Unlike murine pDCs that require TLR system for IFN-α production, murine cDCs and fibroblasts display strong RIG-I-dependent type I IFN response following RNA virus infection.^42^ Macrophages also show strong RIG-I activation upon viral infection, with increased RIG-I signaling in alveolar macrophages infected by IAV and RSV.^289^ Alveolar macrophages are the major source of type I IFN during RSV infection via MAVS-dependent RLR signaling.^339^ In human primary NK cells, IAV triggers NF-κB activation and type-I IFN response via RIG-I.^340^ Early activation of RIG-I signaling contributes to quick and potent NK cell responses that control pulmonary IAV replication and promote survival in mice.^341^ Furthermore, defective IAV RNA increases the surface presentation of human leukocyte antigen proteins in human lung epithelial cells via RIG-I/MAVS pathway.^342^ The RIG-I mRNA levels are also upregulated in human mast cell line following IAV challenge, mediating cytokine and chemokine production.^343,344^

#### MDA5

The role of MDA5 during IAV infection is less understood. Although RIG-I is regarded as the primary sensor of IAV, MDA5 also contributes to the cellular defense against IAV.^345^ MDA5 mRNA levels are higher in PBMCs and leukocytes of influenza patients than in healthy controls, correlating with increased IFN-γ, IL-10, IL-8, IP-10 and MCP-1 levels.^293,338^ In human airway epithelial cells, IAV-induced IFN-β induction is partly dependent on MDA5.^311^ Similar to RIG-I, MDA5 expression in uninfected epithelial cells is suppressed by exosomes released from IAV-infected epithelial cells, which facilitates viral spread.^333^ MDA5 expression is also increased in human mast cell line and murine eosinophils exposed to IAV.^343,346^

#### Other RNA sensors

Besides major RNA receptors, the OAS/RNase L system aids the response to viral pneumonia by activating NLRP3 during IAV infection. RNase L-cleaved RNA promotes NLRP3 inflammasome activation with DHX33 and MAVS.^347^ Nucleotide-binding oligomerization domain-containing 2, an intracellular pattern recognition receptor, senses viral ssRNA genome and induces type I IFN response via MAVS-IRF3 axis in cells infected with IAV or RSV.^348^ Some proteins synergize with RIG-I to promote viral RNA recognition. For instance, DDX6 serves as a co-sensor of RIG-I in IAV infection, binding directly to viral RNA to enhance RIG-I signaling and IFN-β induction.^349^ DEAH-box RNA helicase DHX16 recognizes IAV RNA segments and directly interacts with viral RNA via its RNA helicase motif, forming a complex with RIG-I to optimize IFN induction during influenza infection.^350^ Unexpectedly, PKR is not required for IFN-α and IFN-β production by murine dendritic cells in response to IAV.^351^

DNA sensors like IFI16 enhance RNA recognition during viral infection. IFI16 binds influenza RNA and RIG-I, boosting RIG-I activation in IAV infection.^352^ In addition, IFI16 upregulates RIG-I expression through direct binding to RIG-I promoter and facilitating RNA polymerase II recruitment to the promoter.^352^ ZBP1 is also a sensor of both DNA and RNA. ZBP1 recognizes IAV genomic RNA with its Zα domain and activates RIPK-3-dependent apoptosis and necroptosis.^353^ However, a subsequent study found that purified RNA is insufficient to activate ZBP1-dependent cell death.^307^ Instead, RIG-I-MAVS-IFN-β signaling upon IAV RNA recognition, ubiquitination and viral ribonucleoprotein complexes are all required by ZBP1 activation and downstream programmed cell death.^307^ Another research further illustrated that ZBP1 senses defective viral genomes and Z-RNA generated during IAV replication, eliciting RIPK3-mediated MLKL activation in the nucleus and downstream necroptosis of IAV-infected cells.^354^ Importantly, ZBP1 requires its Zα2 domain to sense RNA and regulate influenza-mediated PANoptosis.^353,355^ ZBP1 knockout mice are characterized by both impaired ability to control IAV infection and increased susceptibility to IAV infection.^353^

### COVID-19

The COVID-19 pandemic has raised global concerns and strained ICUs, with 15-30% of hospitalized patients developing COVID-19-associated ARDS, which has a poor prognosis due to complications and high mortality.^356,357^ Incredible numbers of studies have been conducted all over the world in order to understand the development of COVID-19 and explore effective therapy. Here, we focus on NA sensing in COVID-19 as a key critical care topic (Table 2).

#### cGAS-STING

COVID-19 had been early regarded as a STING disorder with delayed and potent IFN response.^358^ STING expression in human blood cells is upregulated during the infection of severe acute respiratory syndrome coronavirus 2 (SARS-CoV-2).^359^ cGAS and STING expression in blood leukocytes is associated with severe or long COVID-19.^360^ Likewise, the lungs of COVID-19 patients present high cGAS-STING activity and type I IFN production in endothelial cells and macrophages,^361^ while the activation of neutrophils by SARS-CoV-2 infectious particles also depends on STING-IRF3 signaling.^362^ Moreover, single-cell immune profiling uncovers high type I IFN activity in T cells and natural killer cells from severe COVID-19 patients while this activity is low in healthy individuals.^363^

It is recently reviewed that self-DNA aggravates the severity of COVID-19 via cGAS-STING-IFN axis and inflammatory cytokine release.^364,365^ Mechanistically, SARS-CoV-2 induces mitochondrial dysfunction in pulmonary endothelial cells and activates cGAS–STING pathway via mtDNA release, leading to type I IFN response and cell death.^361^ The mtDNA-mediated cGAS-STING activation restricts SARS-CoV-2 replication at a post-entry stage in angiotensin converting enzyme 2 (ACE2)-positive airway-derived cell lines.^366^ The same scenario could be found in human lung epithelial cell lines, as SARS-CoV-2 induces cytoplasmic accumulation of chromatin and mtDNA that activates cGAS-STING-induced IFN production.^367,368^ Furthermore, genomic DNA and mtDNA from infected human airway epithelial cells is taken up by PBMCs, triggering inflammasome activation and IL-1β production in SARS-CoV-2-primed PBMCs in a STING-dependent way.^369^ Cell fusion and formation of micronuclei, on the other hand, could be induced by SARS-CoV-2 spike protein and contribute to cGAS-STING activation as well.^370,371^

#### TLR9

In severe COVID-19 patients, TLR9 activity is elevated compared to both healthy controls and patients with mild COVID-19.^296,372^ The mRNA level of TLR9 in nasopharyngeal epithelial cells is higher in COVID-19 patients than in non-COVID-19 controls, which correlates with clinical parameters and inflammatory biomarkers of disease severity such as neutrophil–lymphocyte ratio and C-reactive protein.^373^ SARS-CoV-2 RNA is present in plasma exosomes from most COVID-19 patients, which significantly upregulates TLR9 expression in CD4^+^ T cells and CD14^+^ monocytes.^374^ TLR9 synergizes with TLR4 to contribute to hyperinflammatory state in severe COVID-19 cases.^296^ Even in recovered patients, TLR9 expression in whole blood remains higher than non-infected individuals.^375^ In human umbilical vein endothelial cells, SARS-Cov-2 could induce mitochondrial dysfunction and mtDNA release, activating TLR9 signaling and inflammatory response.^376^ Whereas, transcript levels of TLR9 in PBMCs from healthy controls, as well as mild and severe-COVID-19 patients, are comparable.^377^ Specifically, single-cell RNA sequencing demonstrates that TLR9 expression in pDCs from severe COVID-19 patients is undetectable.^378^

#### AIM2 inflammasome

The activation of AIM2 inflammasome is mainly located in monocytes during COVID-19.^379^ Junqueira et al. found that SARS-CoV-2 uptake by monocytes triggers pyroptosis through AIM2 and NLRP3 inflammasomes, halting virus production.^380^ Furthermore, the plasma levels of pyroptotic makers (gasdermin D, interleukin-1 receptor antagonist and IL-18) are elevated in COVID-19 patients and correlate with disease severity.^380^ AIM2 inflammasome activation may be linked to lung fibrosis in COVID-19. In post-COVID patients with fibrosis, PBMCs show heightened responses to AIM2 ligand poly(deoxyadenylic-tymidylic) with increased pro-fibrotic cytokine production.^381^ Although host mtDNA is speculated to activate the AIM2 inflammasome in COVID-19,^380^ direct experimental evidence is absent.

#### Other DNA sensors

ZBP1 expression is higher in immune cells from COVID-19 patients, particularly non-survivors.^382^ IFN-induced ZBP1 activation causes macrophage death and increases lethality in infected mice^382^, though its activation by DNA remains unclear.

#### TLR3

TLR3 mRNA expression in blood cells from COVID-19 patients is associated with disease severity and prognosis.^383,384^ Among severe COVID-19 patients, those with mechanical ventilation or die have lower mRNA levels of TLR3 in circulating leukocytes compared with those with supplementary oxygen and survive.^385^ TLR3 expression levels in blood also negatively correlate with lung function and neurological outcome in ICU patients with COVID-19.^386^ Patients with secondary bacterial infections exhibit upregulated TLR3 compared to those without.^387^ However, TLR3 expression levels in nasopharyngeal specimens are comparably between ICU and non-ICU patients with COVID-19.^388^

Serum and endotracheal aspirate samples from COVID-19 patients in ICU potently activate TLR3 signaling in TLR reporter cells.^389^ Plasma exosomes from COVID-19 patients contain viral dsRNA that activates TLR3 and stimulates the production of IL-6 and TNF from PBMCs.^374^ In lung epithelial cells, TLR3-dependent type I IFN induction is required to mediate immune response against SARS-CoV-2.^390^ In addition, TLR3 restrains COVID-19-associated lung vascular remodeling, as evident by that TLR3 knockout augments pulmonary artery remodeling and endothelial apoptosis in SARS-CoV-2-infected mice.^391^ Interestingly, TLR3 deficiency increases the susceptibility of mice to SARS-CoV-2 but does not increase the mortality.^392^ Instead, mice with TRIF deletion are more vulnerable to COVID-19, as evident by exacerbated weight loss, viral burden, mortality, systemic inflammation, infiltrating immune cells, respiratory dysfunction and lung pathology.^392^ Besides, SARS-CoV-2 induces cellular senescence via TLR3.^393^

#### TLR7/8

TLR7 and TLR8 are linked to COVID-19 severity.^394–396^ TLR7 expression is higher in bronchoalveolar lavage cells and circulating neutrophils from severe patients compared to controls.^372,384^ SARS-CoV-2 upregulates TLR7 expression in mouse lungs as well.^397^ One research revealed more TLR8 mRNA than TLR7 mRNA in bronchioloalveolar aspirates from COVID-19 patients.^398^ However, circulating leukocytes in COVID-19 patients show upregulated TLR7 and downregulated TLR8.^385^ In contrast, recovered individuals have higher TLR7 and TLR8 mRNA levels in blood samples compared to unvaccinated and uninfected individuals.^375,399^ Furthermore, a single-cell RNA sequencing study found lower TLR7 and TLR8 mRNA levels in bronchoalveolar lavage fluid from severe COVID-19 patients compared to mild cases.^400^ Reduced TLR7 and TLR8 expression in bronchoalveolar lavage cells is also found in non-survived COVID-19 cases compared to the survivors.^387^

TLR7 knockout mice show impaired IFN induction, viral clearance and antibody production, leading to increased disease severity in SARS-CoV-2 infection.^397^ In human PBMCs, SARS-CoV-2 triggers strong type I and III IFN responses along with inflammatory cytokine and chemokine production via TLR7/8 signaling, even without viral replication.^401^ Likewise, GU-rich ssRNA derived from SARS-CoV-2 genome triggers TLR8-dependent IL-1β production in human macrophages.^402^ In neutrophils, SARS-CoV-2 ssRNA activates TLR8 and subsequent production of TNF, CXCL8, and interleukin-1 receptor antagonist as well as NET release.^403^ SARS-CoV-2 infectious particles further activate human neutrophils to release IL-8, produce reactive oxygen species and form NETs in a TLR7/8-dependent way.^362^ Besides, SARS-CoV-2-primed platelets derive EVs and deliver miRNA to neutrophils where miRNA-21 and miRNA let-7b activates TLR7/8, enhances NET formation in a NADPH oxidase-dependent manner, and upregulates IL-1β, TNF and IL-8 expression.^404^ Surprisingly, TLR7 exists on the cell membrane of human red blood cells (RBCs) with the capacity to bind to SARS-CoV-2 RNA in vitro, but its biological function is unknown.^405^

The interaction between SARS-CoV-2 ssRNA and TLR7/8 activates human DCs and drives IFN production and Th1 polarization.^406^ In particular, TLR7-dependent type I IFN production by pDCs is essential for antiviral immunity against SARS-CoV-2.^390^ pDCs, though resistant to SARS-CoV-2 infection, can sense the virus and produce IFN-α and IFNλ1 via the TLR7-MyD88 pathway.^407,408^ TLR7 also regulates macrophage-pDC interactions in COVID-19.^409^ Lung macrophages, though not directly infected, can sense SARS-CoV-2 through phagocytosis of infected cells, release IL-6 and TNF as well as activate pDCs via cell contact, which boosts IL-6 production in monocyte-derived macrophages.^408,409^

#### RIG-I

Compared to healthy subjects, COVID-19 patients display a decrease in RIG-I mRNA levels in blood samples.^410^ Plasma level of RIG-I protein is associated with disease progression, control of viral shedding, SARS-CoV-2-specific T cell response and the amount of S protein-binding IgG in COVID-19 patients.^411^ Analysis of nasopharyngeal swabs showed that RIG-I in nasopharynx is activated by intracellular viral RNA.^412^ Low expression of RIG-I in nasopharynx is a risk factor for severe pneumonia or ARDS among SARS-CoV-2-infected patients.^413^ Single-cell RNA sequencing of nasal swabs showed that children have higher RIG-I expression in upper airway epithelial cells, macrophages and dendritic cells than adults in early infection.^414^ This, along with more immune cells in the airway and stronger cytokine-mediated interaction with epithelial cells, may explain the lower incidence of severe COVID-19 in children.^414,415^

RIG-I mRNA in circulating leukocytes is elevated in SARS-CoV-2 patients compared to healthy donors.^338^ Transcriptome analysis showed RIG-I upregulation in pulmonary endothelial cells of mice with SARS-CoV-2 pneumonia.^416^ Additionally, single-cell RNA sequencing revealed RIG-I transcription in mesenchymal stem cells from bronchoalveolar lavage fluid of severe COVID-19 patients.^417^ Mechanistically, RIG-I but not MDA5 recognizes SARS-CoV-2-derived small viral RNAs bearing duplex structures and 5’-triphosphates, contributing to a delayed but robust IFN response in the late stage of COVID-19.^418^ RIG-I also binds to the 3’ untranslated region of the SARS-CoV-2 RNA that directly blocks viral RNA-dependent RNA polymerase and abrogates SARS-CoV-2 replication in human lung cells in a MAVS- and IFN-independent manner.^419^ On the other hand, self-assembling short duplex RNAs trigger IFN induction via binding directly to RIG-I and protect against SARS-CoV-2 in human lung airway and alveolus chips as well as mice.^420^ Importantly, SARS-CoV-2 detection in lung epithelial cells via RIG-I is proposed as a key driver of pro‐inflammatory macrophage activation that triggers hyperinflammatory state in COVID-19.^421^ One study suggests that RIG-I deletion does not impact IFN response but is linked to increased expression of ACE2, the SARS-CoV-2 entry receptor, in human epithelial cells.^422^

#### MDA5

MDA5 is activated by SARS-CoV-2 RNA in nasopharynx.^412^ Like RIG-I, MDA5 expression is higher in airway epithelial cells and immune cells in children than in adults, possibly explaining lower severe COVID-19 rates in children.^414,415^ In addition, upregulated MDA5 is observed in circulating leukocytes, PBMCs and nasopharyngeal epithelial cells from COVID-19 patients compared to healthy controls.^338,423,424^

MDA5-MAVS axis is requisite for type I and III IFN induction in human bronchial epithelial cells and monocytes as a part of defense against SARS-CoV-2 infection.^422,425^ MDA5-mediated SARS-CoV-2 detection in lung epithelial cells is thought to drive macrophage activation and the hyperinflammatory response.^421^ Furthermore, SARS-CoV-2 infection triggers a delayed MDA5-dependent IFN response, limiting viral replication in pluripotent stem cell-derived airway cells.^426^ However, a study found that MDA5-mediated IFN induction fails to control viral replication in lung epithelial cells.^427^ For further reference, the role of MDA5 in COVID-19 is discussed in another review, particularly in the aspects of anti-MDA5 antibody and genetic variation.^428^

#### Other RNA sensors

PKR and OAS-RNase L are activated in SARS-CoV-2-infected lung cell line Calu-3 and A549.^429^ Mesenchymal stem cells in bronchoalveolar lavage samples from severe COVID-19 patients exhibit upregulated PKR compared with cells from mild patients.^417^ Additionally, autosomal recessive OAS-RNase L deficiencies are responsible for SARS-CoV-2-related multisystem inflammatory syndrome in children.^430^

### Fungal pneumonia-related ARDS

Respiratory fungal infection typically occurs in immunocompromised patients. Although fungi are not the major causes of ARDS, *Aspergillus fumigatus* and *Pneumocystis jirovecii* are closely associated with ARDS.^263,264,431^ TLR9 in macrophages and DCs participates in the detection and antifungal activities against *Aspergillus fumigatus* and *Pneumocystis jirovecii*.^432,433^ The cGAS-STING pathway is activated in *Aspergillus fumigatus*-infected mice, aiding host defense.^434^ Most DNA sensor ligands are fungal DNA,^172^ but the role of self-DNA sensing in fungal pneumonia remains underexplored, requiring further research.

Likewise, RNA sensing mediates antifungal immunity. TLR3-TRIF pathway is involved in epithelial cell-mediated protective tolerance to *Aspergillus fumigatus* by activating indoleamine 2,3-dioxygenase, a key regulator of T cells.^435^ The MDA5-MAVS-dependent IFN response to *Aspergillus fumigatus* dsRNA activates neutrophil antifungal responses in mice.^436^

## Nucleic acid sensing in sepsis

Sepsis is a life-threatening condition caused by a disordered immune response to infections, leading to organ dysfunction and mortality.^437,438^ The heterogeneity of sepsis patients hinders understanding its pathophysiology and developing personalized treatments.^439^ An increasing number of studies highlight the release of NA during sepsis, emphasizing a potential critical role of NA sensing in the pathophysiology of sepsis.

### DNA sensing in sepsis

#### TLR9

TLR9 expression in circulating neutrophils is higher in critically ill patients who do not survive within 30 days.^440^ The protein levels of TLR9 in monocytes from septic patients and controls are comparable,^441^ while soluble TLR9 in serum is lower in septic patients than healthy individuals.^442^ The activation of TLR9 in neutrophils is associated with poor outcome in cecal ligation and puncture (CLP)-treated mice, which downregulates CXC chemokine receptor 2 expression and reduces neutrophil migration to the site of infection.^443^ In fibroblastic reticular cells, the activation of TLR9 signaling reduces their chemokine generation and suppresses the recruitment of peritoneal B cells in CLP-challenged mice, facilitating the development of sepsis.^444^ TLR9 deletion further promotes the development of bacteremia in mice after intranasal administration of *Streptococcus pneumoniae*.^445^

Bacterial DNA enhances LPS-induced proinflammatory response in mouse macrophages partly in a TLR9-dependent manner.^166^ Bacterial CpG motifs activate TLR9, triggering cytokine release in sepsis.^446,447^ Their administration increases TNF production and mortality in CLP-treated mice.^448,449^ However, another study demonstrated that CpG-ODN-treated mice exhibit reduced lung injury, decreased bacterial load in peritoneal fluid and lower mortality following CLP compared with control counterparts.^450^ Besides, TLR9 senses fungal unmethylated CpG-DNA and triggers antifungal immune response via a MyD88-dependent manner in candidiasis and cryptococcosis.^172,451–453^ *Candida* DNA pretreatment could protect against disseminated *Candida albicans* infection, which could be reversed by Chloroquine, a endosomal TLR inhibitor.^454^

On the other hand, TLR9 is associated with sepsis-related anemia. TLR9 on the surface of RBCs could bind to free DNA derived from mitochondria, bacteria and plasmodia.^59^ The DNA-bound RBCs lose normal morphology and function, promoting RBCs clearance by splenic macrophages and causing acute anemia in a TLR9-dependent manner.^59^ Septic patients with anemia are characterized by increased levels of RBC-associated mtDNA compared with septic patients without anemia.^59^

#### cGAS-STING pathway

In LPS-stimulated macrophages, bacterial DNA enhances proinflammatory response in a cGAS-dependent manner.^455^ Interestingly, membrane vesicles from gut microbiota contain bacterial DNA that could enter circulation to prime systemic antiviral immunity and enhance the clearance of HSV-1 and vesicular stomatitis virus via cGAS-STING-IFN-I axis.^456^ The oxidized mtDNA enters the cytosol to activate cGAS-STING pathway in LPS-primed mouse macrophages stimulated with NLRP3 activators.^236^ LPS also activates GSDMD that forms mitochondrial pores and induces mtDNA release into the cytosol in endothelial cells.^457^ Released mtDNA activates cGAS-STING pathway and impairs vascular regeneration by suppressing yes-associated protein 1 signaling in septic mice.^457^ An interesting study reported that cGAS is related to sepsis-associated low blood pressure independently of STING.^458^ Specifically, cGAS in vascular endothelial cells detects cytosolic DNA and generates cGAMP. The cGAMP is then exported from endothelium to neighboring vascular smooth muscle cells via multidrug resistance protein 1 and volume-regulated anion channel. This transportation activates cGMP-dependent protein kinase 1 in vascular smooth muscle cells and triggers vessel relaxation, contributing to hypotension and tissue hypoperfusion during sepsis.^458^ On the other hand, STING activation triggers necroptosis in septic mice. Necroptosis further enhances STING signaling via the blockage of STING-dependent autophagy, STING degradation and extracellular release of STING.^459^ STING also drives septic thrombosis, as platelets with active STING exhibit enhanced granule secretion, and could interact with neutrophils to promote NET formation in sepsis.^460^ NETs could enhance thrombin generation via both intrinsic pathway of coagulation and a platelet-dependent mechanism.^461–463^ EVs released from neutrophils undergoing NETosis could be engulfed by macrophages and initiate cGAS-STING-dependent IL-6 production presumably via inducing mtDNA release.^464^ In addition, mtDNA, nDNA and bacterial DNA have procoagulant and platelet-stimulating effects,^465^ but whether they act through the cGAS-STING pathway or other signaling mechanisms remains unclear.

STING signaling can also induce immunosuppression in sepsis. In septic mice, cytoplasmic mtDNA accumulation in splenic DCs activates STING, leading to immunoparalysis with reduced costimulatory molecules, increased IL-10 and impaired T cell proliferation.^466^ Moreover, STING activates poly(ADP-Ribose)-polymerase 1 and poly(ADP-ribose) polymer, and triggers necrotic death of T cells in sepsis.^467^ STING also induces apoptosis in CD4^+^ T cells from LPS-treated mice.^468^

#### AIM2-like receptors

The association between AIM2-like receptors and DNA in sepsis is insufficiently studied. The gene expression of AIM2, IL-1β and IL-18 in PBMCs from patients with septic shock exhibits only an increased tendency (and not significance) when compared with healthy controls.^469^ IFI204, the murine homolog of human IFI16, mediates bacterial clearance, macrophage inflammation response and extracellular trap formation during *Staphylococcus aureus* infection, indicating the vital role of IFI204 in host defense against bacterial infection.^117^ Nevertheless, whether AIM2-like receptors are activated by non-self- or self-DNA in sepsis remains inadequately understood, therefore further investigation is needed.

### RNA sensing in sepsis

#### TLR3

TLR3 expression in PBMCs correlates with disease severity, elevated cytokine levels, and 28-day mortality in patients with sepsis compared to healthy controls.^470^ LPS also induces TLR3 upregulation in murine alveolar macrophages.^471^

#### TLR7/8

TLR7 expression is increased in human neutrophils^384^ and murine splenocytes^472^ during sepsis. EVs in septic mice contain miRNAs that induce proinflammatory cytokine production by macrophages via the TLR7-MyD88 pathway.^473^ TLR7 deficiency reduces cytokine levels, improves bacterial clearance, promotes leukocyte recruitment and phagocytosis, alleviates peritoneal inflammation and AKI, and decreases mortality in murine sepsis models.^474^ TLR7 is involved in coagulation in murine sepsis, with TLR7 knockout mice showing preserved clotting, platelet counts and tissue factor levels.^475^ miRNA-146a stimulates tissue factor production via TLR7, while TLR3 knockout has little effect on coagulopathy.^475^ TLR7 also mediates platelet activation and platelet-leukocyte aggregate formation during CLP-induced sepsis.^476^

Further studies showed increased serum TLR8 expression in pediatric sepsis and identified it as a key immune-related gene in septic shock progression.^477,478^ However, the role of TLR8 in sepsis is much less well understood than that of TLR7.

### Sepsis-induced organ dysfunction

Sepsis-induced organ dysfunction is partly contributed by NA sensing. For instance, DNA–binding nanoparticles could protect against sepsis-induced multi-organ injury in mice by scavenging DNA.^479^ STING activation could exacerbate sepsis-induced multi-organ injury by promoting cGAS- and IFN-dependent ferroptosis in macrophages.^480^ Here, we discuss the role of NA sensing in sepsis-induced dysfunction in lungs, liver, brain, heart, kidneys and gut.

#### Sepsis-induced acute lung injury

It is speculated that cell-free mtDNA but not nDNA in lungs aggravates sepsis-induced ALI by activating alveolar macrophages.^481^ mtDNA drives lung injury in sepsis by activating TLR9-MyD88-NF-κB pathway.^482^ Intraperitoneal and intratracheal administration of mtDNA could induce ALI in a TLR9-dependent manner.^483,484^ TLR9 deficiency, on the other hand, improves survival of CLP-induced ALI mice.^485^ Additionally, RBCs bind to cell-free mtDNA via TLR9 and reduce lung injury by scavenging DNA.^58^ Moreover, LPS induces ZBP1-mediated necroptosis that triggers mtDNA release and activates TLR9-NF-κB pathway in macrophages, leading to lung injury in mice.^486^ It has been recently shown that ZBP1 deletion inhibits macrophages from pro-inflammatory polarization and pyroptosis by altering their metabolic status, thereby attenuating inflammatory cascade and endothelial damage in septic mice.^487^

Activated STING is detected in PBMCs from patients with sepsis-induced ALI.^488^ In addition, the mtDNA-cGAS-STING-NLRP3 axis contributes to LPS-induced ALI.^489^ In particular, STING impedes lysosomal acidification and disturbs autophagic flux in macrophages via IFN, aggravating sepsis-induced ALI.^488^ In the context of AIM2 inflammasome activation, the expression of AIM2 in immune cells within the lungs increases during LPS-induced ALI.^490^ The activation of AIM2 inflammasome is triggered by NETs and induces alveolar macrophage pyroptosis.^280^

Importantly, the formation of NETs during sepsis is trigged by microbial components or endogenous danger signals, and regulated by complex mechanisms presumably including HMGB1-TLR9-MyD88 pathway.^491,492^ NETs activate STING pathway in endothelial cells via TLR2 and cause inflammation and coagulation in the lungs, thereby contributing to the poor outcome of sepsis-induced ALI.^493^ Exogenous administration of NETs further aggravates lung injury, which could be reversed by STING inhibitors or knockdown of STING.^494^

Studies suggest TLR3 has little role in CLP-induced ALI, as its transcript level decreases in septic murine lungs, and TLR3 deletion does not protect CLP-treated mice from ALI.^485,495^ On the other hand, TLR7 in macrophages senses extracellular miRNA-146a-5p, triggers pulmonary inflammation and endothelial barrier dysfunction via TNF, contributing to sepsis-induced ARDS.^496^ Besides, MDA5 promotes nuclear translocation of IRF3 to initiate the transcription of signal transducer and activators of transcription 1, activating macrophage M1 polarization in septic ALI.^497^

#### Sepsis-induced acute liver injury

The cGAS-STING signaling is activated in sepsis-induced acute liver injury, along with enhanced type I IFN responses and hepatocyte death.^498^ Mitochondrial fission and formation of VDAC1 oligomer pores trigger cytosolic mtDNA release in Kupffer cells, which is further enhanced by mitochondrial oxidative stress.^499^ STING signaling in Kupffer cells is highly activated by mtDNA in a dynamin-related protein 1-dependent manner.^499^ Both knockout and pharmacological inhibitor of cGAS or STING could restore liver function, suppress systematic inflammatory response and protect against acute liver injury in septic mice.^498,499^ Additionally, CLP mouse model also exhibits high TLR9 expression in liver mononuclear cells.^448^ The mitochondrial biogenesis in hepatocytes during septic acute liver injury is also mediated by autophagy via mtDNA-TLR9 signaling.^500^ Pretreatment with CpG-DNA significantly increased serum TNF, aspartate aminotransferase and mortality in mouse LPS-induced hemorrhagic liver failure.^501^ However, an early study proposed that although CpG-ODN exacerbates early hepatic injury in LPS-stimulated mice, it offers long-term protection against LPS hepatotoxicity.^502^ Besides, application of DNase I and AIM2 siRNA confirmed that NETs released during LPS-induced ARDS trigger pyroptosis of alveolar macrophages in a DNA- and AIM2-dependent way.^280^

#### Sepsis-associated encephalopathy

In neonatal rat with sepsis-associated encephalopathy, TLR9 triggers neural PANoptosis via p38 MAPK pathway.^503^ The phosphorylation of STING in hippocampus is strongly enhanced in septic mice.^504^ STING signaling facilitates neuronal necroptosis and cognitive dysfunction, leading to the development of sepsis-associated encephalopathy.^504^

A study found that miRNA-146-5a levels increase during sepsis and are sensed by TLR7, promoting monocyte and neutrophil recruitment, blood-brain barrier disruption and encephalopathy in mice.^505^ The research group also discovered that plasma EVs from septic mice activate microglia and cerebral immune responses via TLR7 and MyD88 signaling.^506^

#### Sepsis-induced cardiomyopathy

STING-IRF3 activation leads to NLRP3 inflammasome formation and sepsis-induced cardiac injury.^507^ CpG-DNA induces cardiac inflammation and impairs cardiomyocyte contractility in a TLR9-dependent manner,^508^ while CpG-DNA pretreatment restores cardiac function in CLP-induced sepsis.^509^ Furthermore, TLR3 knockout mice show improved cardiac function, reduced inflammation and better survival in CLP-induced sepsis, but these benefits are reversed by adoptive transfer of wild-type bone marrow stromal cells.^510,511^ In addition, TLR7 deficiency aggravates LPS-induced cardiac dysfunction, while TLR7 overexpression improves Ca^2+^ handling in ventricular myocytes and restores cardiac function via cAMP-protein kinase A-phospholamban pathway.^512^

#### Sepsis-induced acute kidney injury

The mtDNA levels in plasma and peritoneal cavity are increased in CLP-treated mice, which contributes to inflammatory cytokine production, tubular damage and septic AKI via TLR9.^513^ TLR9 activation in DCs induces IL-17A production from γδ T cells, facilitating the development of sepsis-induced AKI.^514^ Additionally, cGAS-STING pathway senses cytosolic mtDNA and promotes NLRP3 inflammasome that contributes to sepsis-induced AKI.^515^ Furthermore, STING mRNA expression levels in blood and protein levels in plasma are significantly higher in patients with sepsis-associated AKI than healthy controls, while the latter positively correlate with disease severity.^516^

#### Sepsis-induced intestinal injury

NETs could trigger endoplasmic reticulum stress via TLR9 and induce intestinal epithelial cell death, impairing intestinal barrier function.^517^ Furthermore, in patients with abdominal sepsis, there is a significant upregulation of STING in PBMCs and intestines, which correlates with intestinal inflammation and enterocyte damage.^518^ Likewise, intestinal STING activation by mtDNA is intense in CLP mouse model, causing intestinal epithelial cell apoptosis and intestinal barrier dysfunction.^518^

## Nucleic acid sensing in trauma

Trauma results from exposure to energy (thermal, kinetic, chemical, etc.) that exceeds a threshold, causing physical injury. It ranges from minor injuries to life-threatening polytrauma.^519,520^ Particularly in the latter case, where many cells are damaged or destroyed, there is a substantial release of immunostimulatory NA, which may contribute to the critical condition of polytraumatized patients.

### DNA sensing

cfDNA is released in both traumatic injury and perioperative trauma, with distinct origins in each scenario (Table 1).^521^ The primary source of cfDNA release is damaged cells from direct trauma, though systemic and secondary factors also contribute. cfDNA and other DAMPs at the injury site can trigger a systemic inflammatory response.^522^ This, in turn, prompts additional cfDNA release through end-organ damage and NETosis.^523^

The level of plasma cfDNA upon admission of trauma patients holds promise for evaluating disease severity,^524–529^ though its prognostic value can fluctuate based on the trauma type. However, a comprehensive study demonstrating cfDNA’s prognostic significance as a trauma marker is lacking, with existing data presenting conflicting results.

cfDNA has been shown to promote the procoagulant component of disseminated intravascular coagulation,^530,531^ which is a severe complication of trauma.^532^ cfDNA can promote coagulation^533–536^ via activation of the contact system, enhanced clot stability^537^ and modulated fibrinolysis in a concentration-dependent manner with high concentrations blocking fibrinolysis via tissue Plasminogen Activator inactivation.^538^ In combination with similar histone-mediated effects, this can led to so called “immunothrombosis”.^539^

The proinflammatory responses are mediated by different DNA recognition pathways. mtDNA released by trauma triggers a TLR9-mediated proinflammatory systemic response and is tied to organ injury such as ARDS.^522,540^ In burn animal models, organ damages were shown to be mediated by cGAS-STING pathway, which detects leaking mtDNA as part of the stress response.^541^

In a similar model, AIM2 inflammasome activation causes a post-injury immunosuppression by triggering T cell apoptosis via Fas ligand-expressing monocytes.^542^ Inflammasome activation in traumatic brain injury patients triggers a GSDMD-dependent neuronal pyroptosis.^543,544^ The NLRP3 inflammasome is activated in burn patients and mediates metabolic dysregulation.^545,546^ NLRP3 activation in an animal burn model can be stopped by inhibiting mtDNA release.^547^

### RNA sensing

The clinical relevance of cfRNA recognized by RNA sensors in trauma is not well studied. One study showed increased plasma RNA levels after trauma in mice and humans.^548^ In a trauma mouse model, the inflammatory response was TLR7-dependent but TLR3-independent, although TLR3 is upregulated in lung contusion cases.^548,549^ In contused mice, dsRNA from injured cells drives inflammation and lung injury via TLR3.^549^ While trauma-related miRNAs influence the immune response,^550^ they are not the focus of this review.

## Nucleic acid sensing in ischemia

Ischemia occurs when blood and oxygen supply are reduced or blocked, often due to artery obstruction. Reestablishing blood flow can worsen cellular dysfunction and tissue damage, leading to ischemia/reperfusion injury (IRI), which commonly affects major organs like the heart, liver, and kidneys, especially after transplantation.^551^ Particularly, NA liberated from injured tissues and downstream sensing pathways activated by NA are gradually recognized as novel contributors of sterile inflammation in IRI (Table 1 and Table 2).^552^

### Myocardial infarction and ischemia/reperfusion injury

#### DNA sensing

Ischemic heart disease is one of the most life-threatening conditions and accounts for around 9 million deaths worldwide in 2017.^553^ Among patients who stay at ICU for non-cardiac diseases, 4–14% of them suffer from acute myocardial infarction (AMI).^554^

The cardiac expression of TLR9 in MI mice and dilated cardiomyopathy patients is significantly increased.^555,556^ Ischemic cardiomyocyte-released cfDNA, including mtDNA, along with free HMGB1, interdependently promote systemic inflammatory responses, activate splenic leukocytes and exacerbate myocardial IRI via the receptor for advanced glycation end products-TLR9 pathway.^557^ In particular, extracellular mtDNA activates TLR9 signaling in murine hearts, aggravating myocardial IRI.^558,559^ Surprisingly, cardiomyocytes themselves express TLR9 and internalize mtDNA from the circulation, which activates NF-κB and induces mitochondrial dysfunction and cell death.^560^ The NLRP3 inflammasome in splenic monocytes is also activated by myocardial IRI, evoking systemic inflammation at the early stage of reperfusion and aggravating cardiac ischemic injury in a mtDNA-TLR9-dependent manner.^561^ Exogenous administration of mtDNA further increases cardiac infarct size via TLR9-p38 MAPK pathway.^559^ Intriguingly, TLR9 is vital for post-MI heart repair. One research found that TLR9 promotes the proliferation and differentiation of myofibroblasts to prevent cardiac rupture in MI.^562^

The cGAS-STING pathway senses DNA released from necrotic tissue in infract heart and impedes cardiac repair.^563^ Cardiogenic cfDNA together with HMGB1 exacerbates myocardial IRI by activating cGAS-STING-IRF3 axis and type I IFN response in splenic pDCs.^564^ Plasma dsDNA increased during ST-elevated MI activates cGAS in platelets and directly potentiates platelet activation and thrombus formation via STING-NLRP3-caspase-1-IL-1β pathway.^565^

In addition, cytosolic release of mtDNA caused by impaired mitochondrial fusion could activate cGAS-STING that facilitates the pathogenesis of diabetic myocardial IRI.^566^ In specific, small EVs derived from cardiomyocytes could be internalized by fibroblasts and deliver mtDNA to activate cGAS-STING pathway that promotes fibroblast proliferation and cardiac fibrosis in mouse myocardial IRI.^567^ Additionally, AIM2 inflammasome is closely associated with the susceptibility of ischemic heart failure in type 2 diabetes mellitus mice, as the impaired mitophagy results in the accumulation of cytosolic mtDNA and the activation of AIM2 inflammasome in cardiomyocytes and cardiac macrophages, which leads to increased cardiomyocyte death and altered macrophage phenotype.^568^ The expression of ASC, caspase-1, IL-1β and IL-18 is increased in leukocytes of coronary artery disease patients, indicating inflammasome activation.^569^ IL-1β and IL-18 facilitates contractile dysfunction in myocardial IR.^570^ IFI16/IFI204 recognizes mtDNA release and activates inflammasome formation post MI, while short hairpin RNA targeting IFI16/IFI204 suppresses inflammasome activation, myocardial apoptosis, cardiac dysfunction and remodeling following MI.^571^ ZBP1 also detects mtDNA, however it unexpectedly protects against MI by suppressing TLR9-mediated inflammation in cardiomyocytes and alleviating cardiac dysfunction.^572^

#### RNA sensing

Ischemia-induced cardiac inflammation seems to be predominantly mediated by TLR3/7/8 signaling.^573^ RNAs released from necrotic macrophages and cardiomyocytes induce myocardial inflammation, cardiomyocyte apoptosis and cardiac dysfunction post myocardial IR partly via TLR3-TRIF pathway.^574^ TLR3 is required for the regeneration of damaged neonatal myocardium following MI via glycolytic-dependent activation of yes-associated protein 1 and downstream miRNA-152 expression.^575^ In addition, TLR3 in cardiomyocytes promotes autophagy induction and contributes to heart failure post MI, while TLR3 knockout suppresses autophagy, reduces infarct size, alleviates heart failure and improves survival.^576^ In line with this, mice with TLR3 deficiency exhibit decreased production of pro-inflammatory cytokines, attenuated cardiac damage and reduced leukocyte infiltration following cardiac IRI.^577^ However, the upregulation of TLR3 in PBMCs is associated with the risk of recurrent MI post coronary stent implantation in elderly patients.^578^

Upregulation of TLR7 expression could be seen in human and murine ischemic myocardium post MI.^579^ To be more specific, high expression of TLR7 is observed in cardiac infarct zone rather than remote non-infarct area of murine MI model.^556^ Furthermore, single-cell RNA sequencing demonstrated that TLR7 transcriptive levels in PBMCs are increased in AIM patients with plaque rupture.^580^ TLR7 is involved in acute cardiac ischemic injury and chronic remodeling. TLR7 knockout reduces acute ventricular rupture risk, cardiac matrix degradation, myocardial damage and inflammation from bone marrow-derived cells.^579^ TLR7-MyD88 signaling in response to extracellular cardiac RNA induces pro-inflammatory cytokine production from murine cardiomyocytes, neutrophils and macrophages.^581^ However, one study also proposed that TLR7-MyD88-IRF5 pathway mediates the harmful role of emotional stress-induced sympathetic outflow in myocardial IRI.^582^

### Hepatic ischemia/reperfusion injury

Hepatic IRI is a complication in liver transplantation, shock and trauma.^583^ Research has revealed that DNA released from necrotic hepatocytes induces cytokine release from neutrophils via TLR9, facilitating the progression of hepatic IRI.^584^ Host DNA may also contribute to AIM2 inflammasome activation in the liver and promote excessive inflammatory response following IR.^585^ Besides, the expression of cGAS and STING in the liver is upregulated in hepatic IRI.^586^ Hepatocyte oxidative DNA damage and extracellular mtDNA release induced by IR activate cGAS-STING pathway in macrophages.^237,586,587^ Elevated mtDNA released from hepatocytes post-IR activates STING signaling in macrophages, worsening inflammation and liver damage.^588^ The increased mtDNA also activates NLRP3 signaling in macrophages from aged livers post IR in a STING-dependent manner.^589^ However, a study found that the deficiency of cGAS but not STING actually exacerbates hepatic IRI by suppressing hepatocyte autophagy and promoting mitochondrial dysfunction and apoptosis.^590^

### Renal ischemia/reperfusion injury

Renal IRI contributes to AKI, leading to renal dysfunction and increased mortality rates.^591^ It is hypothesized that mtDNA is released from dying renal cells and potentially activates TLR9 in kidney and remote organs.^592,593^ Interestingly, intestinal TLR9 protects against ischemic AKI and remote organ dysfunction by inhibiting IL-17A release from Paneth cells.^592^ mtDNA also activates cGAS-STING signaling in the kidneys and increases inflammation that exacerbates renal IRI.^594–596^ Additionally, cGAS-STING activation promotes renal fibrosis following IR.^597^ Likewise, the expression of STING in kidney tubules is significantly increased.^598^ Besides, in post-renal transplantation patients, the protein levels of AIM2, ASC and cleaved caspase-1 are significantly increased compared with healthy controls.^599^ Furthermore, renal IRI triggers TLR3 upregulation and early TLR3 activation in kidney, which is associated with renal inflammation and tubular damage post renal RI in mice.^600^ Single-cell RNA sequencing of renal samples from ischemic AKI patients showed that injured proximal tubules express genes enriched in RIG-I signaling.^601^

## Nucleic acid sensing beyond critical illness

NA sensing in non-critical conditions including cancer, autoimmune disease, metabolic disorder and aging has been extensively discussed for years, earlier than the exploration in critical illness. Therefore, understanding the contribution of NA sensing to non-critical disease sheds light on corresponding research in the context of critical illness. Nevertheless, the exact molecular mechanisms of receptor activation and signal transduction of the NA sensors discussed for the role in illness here, are not the focus of this review. Instead, for a complete picture regarding the structure and processing of their ligands as well as all signaling events following their activation, we refer to the exhaustive reviews from others.^3–6^

### Cancer

In recent years, NA sensing has been studied mostly for its role in cancer immunotherapy. Data on the development of synthetic agonists for NA sensors such as cGAS, STING, RIG-I, TLR3/7/8/9 and their efficacy in cancer immunotherapy have been extensively reviewed elsewhere.^602^ The notion that targeted activation of these sensors restricts the growth of transplanted tumors, sparks the questions, if their physiological activation exerts tumor-suppressive activities over a life time and thus acting tumor suppressive. Looking at knockout mice, so far, there is no report of premature spontaneous tumor development in cGAS, STING, RLR or IFN-α/β receptor-deficient mice. Deficiency for STING or cGAS and STING had no impact on the tumor-free survival of mice lacking the tumor suppressors p53^603^ or RnaseH2,^604^ respectively, suggesting that in these tumor prone models cGAS-STING signaling is redundant for the control of spontaneous endogenous tumorigenesis in mice. However, cGAS,^605^ STING,^605^ RIG-I,^606,607^ MAVS^608^ and IFN-α/β receptor 1-deficient mice^609^ develop a higher tumor burden in an azoxymethane/dextran sulfate sodium colon cancer model. Furthermore, targeted sequencing of the RIG-I gene in 425 blood samples from colon cancer patients revealed two types of frame-shift mutations in five different patients.^607^ In mice harboring these mutations RIG-I protein levels were reduced, but at the same time the inflammatory response was increased. Consequently, RIG-I frames-shift mice had a higher tumor burden in following azoxymethane/dextran sulfate sodium treatment, further corroborating a role of the RLR-pathway in controlling tumor development.^607^ Collectively, these data imply a role of intracellular NA sensors in controlling tumor development in response to certain environmental triggers with inflammatory properties.

Frequently, the relevance of NA sensing pathways in tumor control is inferred by studying their transcript levels reported for different tumor entities in cohort datasets such as TCGA. However, transcription of these sensors is regulated by inflammatory stimuli, which themselves are subject to endogenous and exogenous triggers that are highly perturbed in cancer patients. Thus, NA sensor expression levels reported in cohort databases likely reflect a dynamic and therefore complex response pattern. With a few exceptions, such as like total absence of expression, in most of these cases studying transcript levels will not allow to draw conclusions about their functional involvement in the pathogenesis of the respective malignancy. For instance, correlation analysis of intratumoral STING transcript levels with overall survival for different cancers implicated high STING expression with better prognosis.^610–612^ Conversely, several other studies associated increased expression and activation of the STING pathway with tumor progression in different cancer entities.^613–615^ These observations illustrate a highly context-dependent role of NA sensing in the control of endogenous spontaneous tumorigenesis^616^ and likely represent a rich area for fundamental discoveries to come.

### Autoimmunity

Acute or low-level chronically increased cytokines increase the density of costimulatory molecules on antigen-presenting cells and thereby increase the chance of activating auto-reactive lymphocytes in the absence of exogenous danger. This scenario most likely explains the association between increased signaling of endosomal TLRs with the development of systemic autoimmunity. This has first been demonstrated by the strict requirement of the DNA sensing TLR9 on B cells in the generation of anti-DNA autoantibodies.^617^ However, for TLR9 there is currently no evidence that the overexpression of the sensor causes spontaneous autoimmunity. In contrast, TLR7 gene duplications^618^ and the escape of TLR7/8 genes from chromosomal X-inactivation^619^ were highly associated with a loss of immunological self-tolerance, suggesting that RNA sensing through these sensors is less controlled compared to DNA sensing by TLR9. In support of this, gain of function mutations in TLR7 were recently found to cause monogenic lupus.^620^ Protein unc-93 homolog B1 is an essential regulator of endosomal TLR activity. Unc-93 homolog B1 variants have been shown to specifically underlie TLR7-dependend early onset, as well as childhood systemic lupus erythematosus (SLE) by regulating TLR7 sorting and stability at the endosomal compartment.^621–625^

The cGAS-STING intracellular DNA sensing pathway has been extensively studied for its role in the development of systemic autoimmunity. Over the last decade, chronic activation or overactivation of the cGAS-STING pathway has been identified to drive a large fraction of monogenic disorders that were grouped together under the term type I interferonopathies.^626^ This work was largely pioneered by work on the molecular mechanisms underlying Aicardi Goutières syndrome (AGS), a rare autoimmune encephalopathy. The majority of AGS cases identified to date are caused by loss of function mutations in genes that control the abundance of extrachromosomal DNA or the accessibility of nDNA for cGAS.^626^ Hence, these mutations increase the amounts endogenous dsDNA ligands of cGAS and overcome the natural threshold for activation of the enzyme. In addition to a clinical overlap in manifestations between AGS and SLE, mutations in AGS genes like *TREX1*, *RNASEH2B* and *SAMHD1* were identified in SLE patients, strongly suggesting them as risk factors of polygenic lupus and as drivers of monogenic subtypes like familial chilblain lupus.^626^ Following these fundamental discoveries, the inflammatory components of many syndromes that are caused by deficiencies in DNA repair enzymes like ATM^627^ or BLM^628^ and others have also been shown to be driven by excessive cGAS-STING signaling. Although several loss of function mutations in genes that restrain spontaneous cGAS overactivation under homeostatic conditions have been identified as drivers of systemic autoimmune conditions, gain of function mutations in the sensor itself have not been identified in the population. In contrast, gain of function mutations in STING lock the protein in a conformational state that constantly triggers trafficking and activation leading to the development of a pediatric inflammatory disease called STING-associated vasculopathy with onset in infancy.^629,630^ Interestingly, clinical symptoms of this disease seem to rather develop due to increased NF-κB as compared to IRF3 signaling, thus diverting from the pure type I interferonopathy paradigm.^631,632^ Activation and deactivation of STING signaling is tightly controlled by balancing between recycling and degradation of the activated sensor, which is achieved through controlling its trafficking between the endoplasmic reticulum, the Golgi and the lysosome.^633^ Defects in the timely and adequate regulation of STING trafficking for instance cause coatomer protein subunit alpha syndrome^634,635^ and Niemann pick disease type c.^636^

Similar to excessive amounts of misplaced DNA, failure to regulate the abundance of endogenous dsRNA can activate dsRNA sensors and drive autoinflammation and autoimmunity.^156,637–639^ However, in the absence of a strong accumulation of endogenous RNA, this process is sensitive to cGAS-mediated priming of MDA5 expression.^603^ Hence, therapeutic targeting of cGAS or STING would abrogate inflammation.^640^ Gain of function mutations in MDA5 render the sensor hyperresponsive to imperfect endogenous ligands or cause its nucleation even in the absence of a ligand, resulting in the development of a spectrum of systemic autoimmune diseases, most notably monogenic AGS^637,638,641^ and such mutations increase the susceptibility to SLE.^642,643^ Gain of function mutations in the gene *DDX58* encoding RIG-I, the sister dsRNA sensor of MDA5, can cause Singleton-Merten syndrome, a systemic autoimmune that is characterized by a prominent bone erosion phenotype.^626,644,645^

### Metabolic disorder

NA sensing can impact on metabolic processes from two perspectives: Metabolic stress, for instance during malnutrition, can compromise organ function leading to the release of NA from damaged or dying cells promoting inflammation. Alternatively, NA release during infection can trigger metabolic reprogramming, increasing metabolic stress and adaptation.^5^ In disease, identifying the primary event is challenging, as both processes respond to feedback regulation. The outcome of NA sensor activation on metabolism depends on the cell type and organ involved.^5,646^

Activation of TLR9 in mouse macrophages that reside in fatty tissue is likely to release chemokines like CCL2 attracting immune cells and driving fatty tissue inflammation as seen in obesity.^647^ Similar disease-promoting effects have been described for TLR9-dependent proinflammatory cytokine release from liver resident macrophages and TLR9-driven release of IFN-α by pDCs in nonalcoholic steatohepatitis^648,649^ and type 1 diabetes,^650^ respectively. Conversely, the activation of TLR9 in B cells can induce a regulatory phenotype characterized by the release of IL-10, which dampens inflammation and protects mice from type 1 diabetes.^651^ Similar observations have been reported for cGAS. Oxidized nDNA or mtDNA has been shown to activate cGAS in resident macrophages of the liver and the fatty tissue but also directly in adipocytes.^499,647,652,653^ This local activation of the cGAS-STING pathway drives inflammation and liver steatosis, ultimately promoting obesity.^652,654,655^ Activation of cGAS in pancreatic islet cells can cause inflammation through the activation of IRF3 and NF-κB pathways.^656^ However, it seems that in these cells, cGAS-STING pathway maintains glucose homeostasis by regulating insulin release through the expression of the PAX6 transcription factor.^657^ More generally, type I IFNs control fatty acid oxidation and metabolically wire the immune response to cell physiology.^658^ For instance, regulatory T cells rely on importing fatty acids for lipid synthesis and impaired uptake of fatty acids results in mitochondrial damage, decreased oxidative phosphorylation and the release of mtDNA that activates cGAS. Under these conditions cGAS activation boosts the suppressive phenotype of regulatory T cells by increasing the release of IL-10.^659^

### Aging

The immune system has been implicated in driving physiological ageing already in the 1960s.^660^ In tissues, immune-driven aging, or “inflammaging”, is marked by impaired leukocytes, arrested non-hematopoietic cells, and increased basal expression of pro-inflammatory cytokines and type I IFNs. The altered dormant state of aged cells is known as senescence.

The cGAS-STING pathway has been implicated in driving senescence and different age-related pathologies.^661,662^ Over a lifetime, cGAS is believed to become sporadically activated by endogenous dsDNA ligands originating from the activity of endogenous retroelements, defective mitochondria or aberrant genome maintenance. Studies in human and mice implicate epigenetic regulators as critical factors that suppress retroelement transcription. For example, Sirtuins (SIRTs) belong to the group of highly conserved deacetylases, which target histones and other proteins to regulate transcription. One of its members SIRT6 ribosylates the transcriptional repressor KRAB-associated protein 1 to enable heterochromatin formation at promoters of long interspersed nuclear elements 1(L1), the only retrotransposition-active family of human endogenous retroelements. With age SIRT6 function declines, leading to an increased transcription of L1, which activated the cGAS-STING pathway and drove premature ageing in *Sirt6*-deficient mice.^663^ Similar observations have been made in ageing human fibroblasts, which after extensive passaging displayed deregulated expression of chromatin regulators as well as L1 restrictions factors, resulting in a cGAS-STING-dependent increase of the tonic type I IFN signal.^664^ Such aged-fibroblasts entered the state of senescence, a hallmark of aged tissues. Intriguingly, most age-related symptoms as well as the increased expression of IFN-regulated genes were reversed by knocking down L1 or blocking reverse transcription, suggesting loss of control over endogenous retroelements as one cause of cGAS-STING-driven inflammaging.^663,664^ A similar mechanism could underly the development of age-related macular degeneration in its incurable end stage geographic atrophy (GA). In GA increased expression of L1 drives reverse transcription of the Arthrobacter luteus elements, which cause mitotic stress leading to the release of mtDNA, another major endogenous ligand for cGAS. In aged retinal pigment epithelial cells of GA patients, activation of cGAS by mtDNA results in non-canonical inflammasome activation and cell death, leading to blindness.^665^ Chronic inflammation of the central nervous system has been implicated in the development of neurodegeneration and cognitive decline. Specifically, in microglia, the central nervous system-resident phagocytes, cGAS is activated through the detection of mtDNA following a loss of mitochondrial integrity and signals through STING. Pharmacologic inhibition of STING reverted central nervous system-intrinsic inflammation and reduced signs of cognitive decline in mice.^666^ Impaired mitochondrial integrity also releases dsRNA into the cytosol where it activates RIG-like receptors RIG-I and MDA5 to drive senescence.^667^

The third important source of endogenous cGAS ligands that drive aging is nuclear genomic DNA. Under physiological conditions, nDNA-induced cGAS activation in age-associated pathologies is associated with a compromised integrity of the nuclear envelope or its complete breakdown caused by altered function or abundance of lamins, causing a group of diseases that are collectively referred to as laminopathies. Under situations of stress, like exposure to ionizing radiation, lamin B1 was found reduced resulting in DNA containing protrusions with no or severely reduced nuclear envelope function and the occurrence of cytoplasmic chromatin.^662^ Such cells activated cGAS and subsequently entered a state of senescence, a hallmark of aging. Similarly, age-related decline in the activity of the yes-associated protein/transcriptional coactivator with PDZ-binding motif transcriptional regulators reduce lamin B expression and thereby weaken the nuclear envelope resulting cGAS-driven senescence.^668^ In the Hutchinson–Gilford progeria syndrome deleterious mutations in lamin A result in a compromised nuclear envelope enabling cGAS to bind to nuclear dsDNA and drive premature inflammaging.^669^ Intriguingly, loss of function mutations in the barrier to autointegration factor are associated with Néstor-Guillermo progeria syndrome,^670,671^ most likely because under physiological conditions barrier to autointegration factor restricts cGAS from accessing nDNA in the situation when the nuclear membrane is compromised.^672^

B cells produce antibodies for protection against infections, toxins and aberrant cells. Under certain conditions, antibodies can be directed against healthy tissues, resulting in autoimmunity. Auto-reactive B cells are enriched in a subpopulation of follicular B cells that are characterized by expression of the integrin alpha X (ITGAX and CD11c) and the transcription factor T-bet.^673,674^ This subpopulation was found expanded in the elderly and hence termed age-associated B cells (ABCs). ABCs have been shown to respond stronger to TLR7 and TLR9 stimuli than to B cell receptor engagement with autoantibody production and the release of several cytokines including TNF, but also IL-10 and IL-4.^673^ In mice, chronologic expansion of ABCs depends on TLR7 signaling, but not on TLR9, implicating sensing of endosomal RNA degradation products as a driver of aging-induced autoimmunity.^674^ TLR7 escapes X-inactivation, resulting in increased TLR7 signaling in immune cells of women.^619^ Intriguingly, the percentage of ABCs among B cells in women over the age of 60 years is doubled compared to younger women or men of any age.^674^ In female rheumatoid arthritis patients the frequency of ABCs positively correlates with age, further supporting an important role of RNA sensing through TLR7 as a driver of age-related autoimmune conditions.^674^

## Therapeutic strategies targeting nucleic acid sensing

### Nucleic acid clearance

The accumulation of NA in critical illness results from both aberrant release and impaired clearance. DNase activity is notably reduced in conditions like COVID-19, influenza, sepsis and trauma. Low DNase levels correlate with increased mortality in COVID-19,^675^ and severe COVID-19 patients show significantly lower plasma DNase levels, hindering NET clearance.^676^ Similarly, influenza and septic patients also exhibit reduced DNase activity.^677,678^ In sepsis, DNase clears NETs to prevent clot formation and host injury.^679^ Trauma patients have elevated circulating DNase upon admission,^680^ while myocardial infarction patients show higher DNase activity.^681^ DNase activity also correlates with cfDNA levels in cardiac diseases including AMI.^220^

**Fig. 5:**
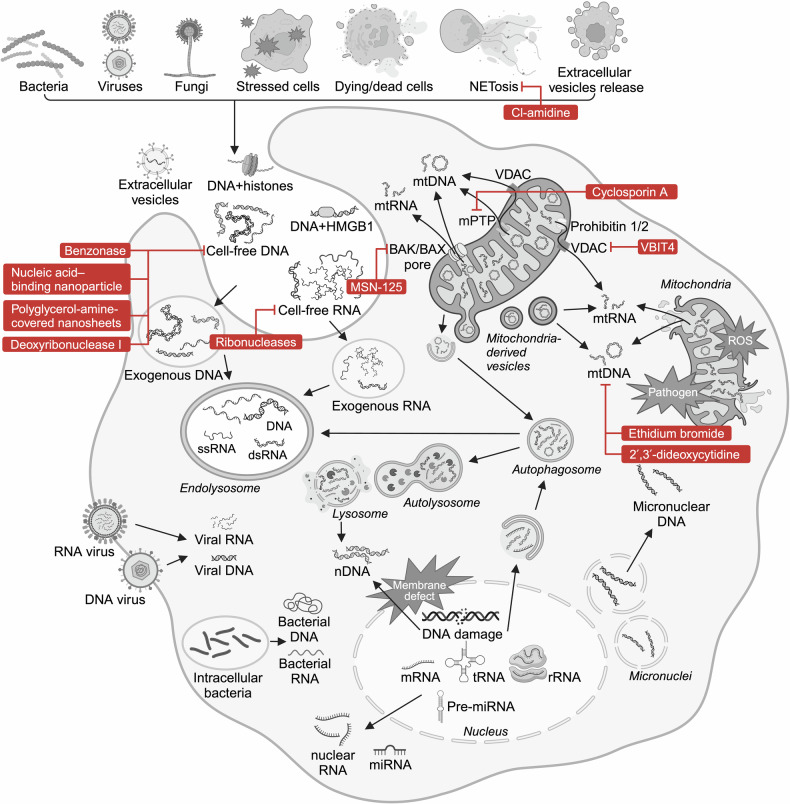
Targeting ligands for nucleic acid sensors offers a potential treatment for critical illness management. Inhibition of nucleic acid release and clearance of nucleic acids ideally limit the activation of nucleic acid sensors at the source. Inhibition of nucleic acid release includes restraining the formation of neutrophil extracellular traps by Cl-amidine as well as suppressing mitochondrial DNA leakage by MSN-125, cyclosporin A and VBIT4. Clearance of nucleic acids includes reducing extracellular DNA and RNA by nucleases and nanomaterials, as well as deleting intracellular mitochondrial DNA by ethidium bromide and 2′,3′-dideoxycytidine. Figure created with BioRender.com

Intravenous administration of recombinant human DNase I, a drug approved by the US Food and Drug Administration, degrades NETs and attenuate coagulopathy in mice with ALI.^682^ DNase I administration also reduces inflammatory cytokine production, organ damage, bacterial translocation and mortality in CLP-treated mice by degrading extracellular mtDNA and NETs.^481,518,683,684^ In myocardial IRI, DNase I is proposed as a cardioprotective agent because clearing extracellular mtDNA and NETs reduces inflammation, suppresses myocardial necrosis and improves cardiac function.^558,685,686^ Correspondingly, DNase I or polyglycerol-amine-covered nanosheets alleviates renal IRI and restores kidney function via scavenging cfDNA.^687–690^ Besides, suppressing cytosolic DNA release and aberrant accumulation is another way of limiting DNA sensing activation. Inhibiting mtDNA release by cyclosporin A (an mPTP inhibitor), VBIT4 (a VDAC inhibitor) and MSN-125 (a BAX/BAK inhibitor) could obstruct cGAS-STING activation and alleviate hepatic IRI (Fig. 5).^586^

In common with DNA clearance, RNA digestion limits RNA sensor activation. In myocardial IRI, RNase degrades serum RNA, reducing myocardial apoptosis, cardiac cytokines and leukocyte infiltration in mice.^574^ In liver IRI, RNase attenuates hepatic damage, inflammatory cytokine release and hepatocyte apoptosis.^691^

### TLR9

**Fig. 6:**
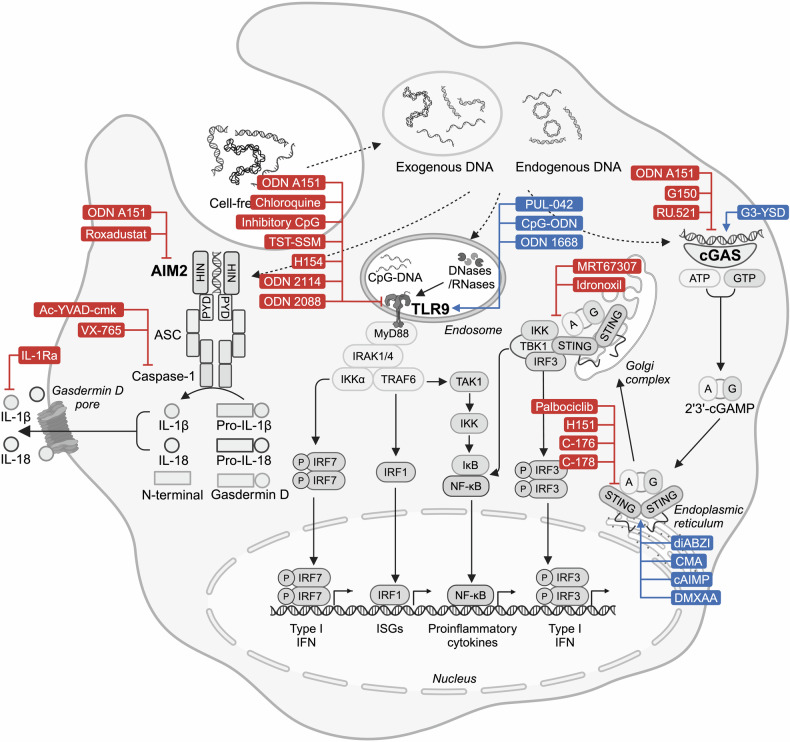
Targeting DNA sensing as a potential therapeutic strategy for critical illness. Manipulation on DNA sensing protects against critical illness. Pharmacological inhibitors (red) or activators (blue) directly regulate key proteins in distinct DNA sensing pathways, including TLR9, cGAS, STING, TBK1/IKKε, AIM2, caspase-1 and IL-1β. Figure created with BioRender.com. Ac-YVAD-cmk N-acetyl-tyrosyl-valyl-alanyl-aspartyl chloromethyl ketone, cAIMP cyclic adenosine-inosine monophosphate, CMA 10-carboxymethyl-9-acridanone, G3-YSD G3-ended Y-form short DNA, IL-1Ra interleukin-1 receptor antagonist, TST-SSM thiostrepton encapsulated in phospholipid sterically stabilized micelles

Exogenous DNA molecules including CpG-ODN are well-investigated agents to target DNA sensors (Fig. 6). However, their therapeutic role is contradictory and largely dependent on the dual functions of DNA sensing pathways in different diseases. CpG-ODN provides protection against infection in mice, promoting pathogen killing and host survival post IAV or bacterial challenges via TLR9-MyD88 and RIG-I-MAVS pathways.^692,693^ In particular, CpG-pretreated mice exhibit resistance to *Staphylococcus aureus* infection partly owning to TLR9-dependent production of bacteria-active antibodies and TLR9-mediated phagocytosis and autophagy in macrophages.^694–696^ In murine model of sepsis, CpG-ODN reduces lung injury, bacterial load and mortality.^450,697^ In addition, CpG-ODN attenuates sepsis- or IR-induced acute cardiac dysfunction.^509,698^ However, opposing findings exist: CpG administration raises TNF levels and mortality in CLP-treated mice, increases aspartate aminotransferase and mortality in LPS-induced liver failure, and induces cardiac inflammation and impairs cardiomyocyte contractility via TLR9.^448,501,508^ PUL-042, a TLR2/6 and TLR9 agonist drug, has been shown to improve survival of in IAV-infected mice.^699^ Besides, our previous study showed that ODN A151, an inhibitor targeting TLR9, cGAS and AIM2, promotes T helper 17 cell responsiveness and increases murine resistance to *Candida albicans* infection.^700^

Importantly, B cell-intrinsic TLR9 induces a tolerogenic response, offering protection against autoimmune diseases like lupus.^701–703^ However, its role in critical illness remains unclear. Notably, intestinal TLR9 inhibits IL-17A release from Paneth cells, protecting against ischemic AKI and remote organ dysfunction.^592^ Therefore, the potential role of TLR9-induced tolerance in critical diseases warrants further investigation.

In contrast, TLR9 genetic deletion and pharmacological inhibitors reduce inflammation, splenic apoptosis, kidney injury, liver dysfunction and mortality in septic mice.^704–707^ TLR9 deficiency also provides cardiac protection and survival improvement in murine sepsis.^510,708^ Intriguingly, transferring TLR9-deficient DCs to wild-type mice could restrain bacterial infection, increase peritoneal granulocytes and decreases mortality post CLP challenge.^706^ In addition, the protective effects of TLR9 inhibitors are observed in renal IRI as well. Nanoparticle-based delivery of selective TLR9 antagonist targeting kidney could reduce renal tubular necrosis and inflammatory response during ischemic AKI.^709^ Specific deletion of TLR9 in renal proximal tubular suppresses tubular necrosis, kidney apoptosis and tubulointerstitial fibrosis following renal IRI.^593,710^ Chloroquine inhibits endosome acidification and TLR function, alleviating renal injury and systemic inflammation in sepsis and renal IRI, possibly through TLR9.^704,711^ The effect of TLR9 deletion in myocardial IRI appears to be controversial. On one hand, TLR9 deficiency could improve cardiac function and suppress myocardial necrosis and inflammation.^558^ On the other hand, TLR9 knockout mice show higher rates of cardiac rupture and mortality after MI, likely due to reduced myofibroblasts, impaired matrix production, increased apoptosis and reduced angiogenesis.^555^

### cGAS-STING

The cGAS-STING pathway is a key target for various diseases, particularly in COVID-19. STING agonists diABZI and adenine monophosphate-inosine monophosphate activate the IFN response and inhibit SARS-CoV-2 replication in human airway cells, stem cell-derived cardiomyocytes and mice.^712–714^ diABZI-4 induces proinflammatory cytokine release, activates immune cells and reduces SARS-CoV-2 load in the lungs.^715^ Inhibition of the cGAS-STING pathway impairs innate immune activation and promotes SARS-CoV-2 replication in human airway cells.^366^ However, excessive cGAS-STING activation may worsen disease, while the STING inhibitor H151 reduces lung inflammation, weight loss and mortality in SARS-CoV-2-infected mice.^361^ TBK1-IKKε inhibitors Idronoxil and MRT67307 also attenuate hyper-inflammation during SARS-CoV-2 infection.^716^ Nevertheless, one study found that STING deficiency had little impact on disease progression, viral load, cytokine release or immune cell infiltration in mice infected with SARS-CoV-2.^717^ Further research is needed to explore the cGAS-STING axis as a potential COVID-19 therapy.

Inhibiting cGAS-STING protects mice from sepsis, suggesting that overactivation of this pathway accelerates sepsis progression. cGAS deficiency improves survival, reduces cytokines, alleviates leukopenia and organ injury in CLP-induced sepsis.^455^ Deletion of cGAS promotes macrophage M2 polarization via mtDNA-mTOR complex 1 pathway, thereby improving sepsis outcome.^718^ STING knockout in CLP mouse model suppresses inflammatory response, restores intestinal barrier dysfunction, inhibits microbial translocation and improves survival and hypothermia, while STING agonist exacerbates these pathological changes.^518,719^

Likewise, targeting cGAS-STING protects against sepsis-induced multiorgan dysfunction. STING deletion, siRNA, and inhibitors H151 and C-176 reduce LPS-induced lung inflammation and ALI by preventing immune cell recruitment to vascular endothelial cells.^488,720^ H151 reduces intestinal injury, restores renal function and improves seven-day survival in septic mice.^721,722^ C-176 alleviates sepsis-induced AKI by suppressing cell senescence, apoptosis, and macrophage infiltration and M1 polarization in murine kidneys.^516,723^ Both knockout and pharmacological inhibitor of cGAS or STING could restore liver function and protect against sepsis-induced acute liver injury.^498,499^ In sepsis-associated encephalopathy, cGAS deletion alleviates cognitive impairment and type I IFN production in the hippocampus.^724^ Moreover, STING deficiency offers cardioprotection by inhibiting myocardial inflammation, cardiomyocyte death and cardiac dysfunction in septic mice.^507^

Manipulation in cGAS-STING pathway is a promising strategy for the management of ischemic disease. The inhibitors of cGAS-STING pathway are proposed as potential therapies for AMI and hepatic IRI.^586,587,725,726^ The deficiency of cGAS triggers M2-like macrophage phenotypes, restricts pathological remodeling, preservers cardiac function and improves the outcome of MI.^563^ cGAS depletion in platelets or the FDA-approved cGAS-STING inhibitor Palbociclib alleviates myocardial IRI in mice.^565^ Furthermore, STING inhibitors reduce organ damage in IRI of the heart, kidneys and liver.^588,727,728^

### Other DNA sensors

Whether AIM2 and ZBP1 are promising targets for clinical treatment lacks sufficient investigation. AIM2 deletion dampens caspase-1 activation in *Streptococcus pneumoniae*-stimulated macrophages.^279^ Roxadustat pretreatment suppresses AIM2 inflammasome activation, restrains kidney damage and preserves renal function, protecting against ischemic AKI in mice.^599^ In addition, ZBP1 knockout protects mice against IAV infection by reducing inflammation and epithelial damage.^306^

### Tarting RNA-sensing pathways

**Fig. 7:**
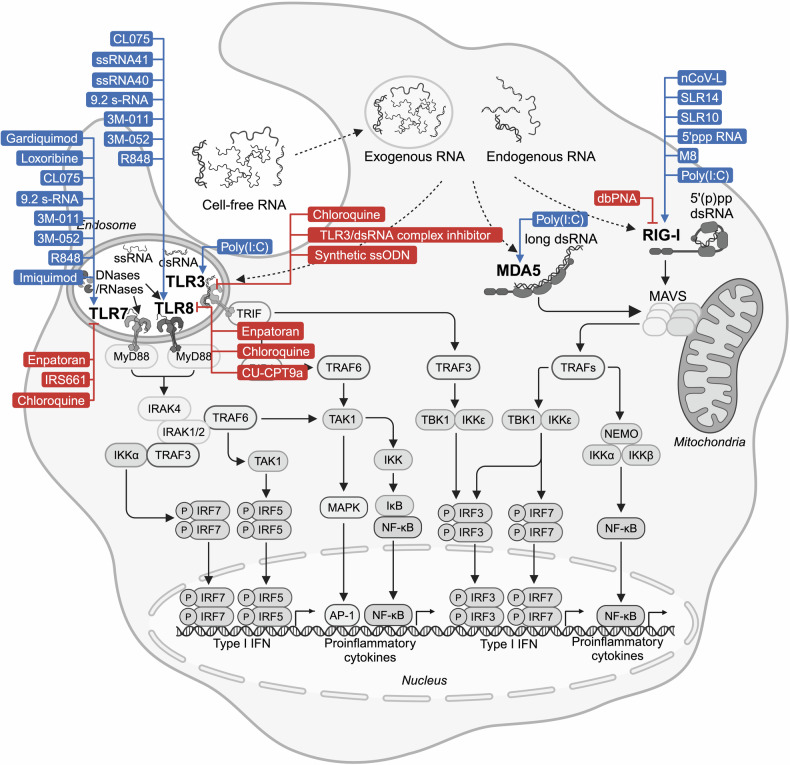
Targeting RNA sensing presents a promising clinical approach for critical illness treatment. Pharmacological inhibitors (red) or activators (blue) directly manipulating RNA sensors including TLR3, TLR7, TLR8, RIG-I and MDA5 offer therapeutic options in critical illness. Figure created with BioRender.com. 5’ppp RNA 5’triphosphorylated RNA, dbPNA double-stranded RNA-binding peptide nucleic acid, IRS661 immunoregulatory sequence 661, SLR stem-loop RNA, ssODN single-stranded oligonucleotide

Poly(I:C), a ligand for TLR3, RIG-I, and MDA5 (Fig. 7), is mainly investigated in experimental models. In mice, poly(I:C) pretreatment boosts pro-inflammatory cytokines, reduces lung damage and virus loads, and protects against IAV.^729^ Treatment with poly(I:C) protects against COVID-19 in mice, with reduced viral load, limited inflammation and increased suvival.^730^ Notably, in vitro poly(I:C) stimulation increases viral entry proteins – neuropilin-1 in lung and nasal fibroblasts, and ACE2 and transmembrane serine protease 2 in nasal epithelial cells.^731^

In mice subjected to CLP and subsequent intranasal administration of *Pseudomonas aeruginosa*, poly(I:C) improves bacterial clearance and promotes survival.^732^ Poly(I:C) pretreatment significantly alleviates liver injury and mortality of LPS-challenged mice.^733^ Additionally, poly(I:C)-activated mesenchymal stem cells display enhanced immunosuppressive capacity and improve the survival of mice with CLP-induced sepsis.^734^ Mesenchymal stem cells treated with poly(I:C) differentiate into the anti-inflammatory phenotype, attenuate renal injury, reduce systemic inflammation and promote antioxidant capacity in mice with renal IRI.^735^ As for acute myocardial IRI, the protective effects of poly(I:C) pretreatment include reducing infarct size, preserving cardiac function, restoring autophagic flux and downregulating the expression of inflammatory cytokines and apoptotic molecules.^736,737^ Nevertheless, the detrimental role of poly(I:C) is reported by many studies. Poly(I:C) triggers hyperinflammation, NET formation and ALI in mice.^738–740^ Mice pretreated with poly(I:C) exhibit impaired clearance of *Streptococcus pneumoniae* and MRSA in the lungs.^741^ Consistent with this, peritoneal macrophage activation and bacterial clearance in septic mice are impaired by poly(I:C) pretreatment.^742^ The mixed results suggest that poly(I:C)'s protective effects depend on disease type and timing, favoring its use as a preventative rather than therapeutic treatment.

### TLR3

TLR3 is key to antiviral immunity, and TLR3 agonists can protect against viral infections. Intranasal pretreatment with TLR3 ligands has been shown to protect mice from lethal IAV challenges.^743^ Yet, studies using knockout mice show that blocking TLR3 signaling protects against IAV. TLR3 knockout mice are more resistant to IAV, with reduced pulmonary inflammation and damage, improved viral clearance and improved survival.^309,744–746^ Correspondingly, TLR3 inhibition by single-stranded ODNs suppresses IAV replication in monocyte-derived DCs and reduces viral loads in murine lungs.^747^ Of note, IAV-infected mice with TLR3 deficiency have a higher risk of developing bacteremia after *Streptococcus pneumoniae* infection, suggesting TLR3’s role in preventing post-influenza secondary infections.^445^ In contrast, TLR3 deletion, inhibition or neutralization enhances alveolar macrophage bactericidal and phagocytic capacity, improving survival in *Klebsiella pneumoniae*-challenged mice,^282^ suggesting a context sensitive benefit/harm by using TLR3 antagonists.

### TLR7/8

Imiquimod, an FDA-approved TLR7 agonist for actinic keratosis and warts, is proposed as a potential anti-SARS-CoV-2 drug,^748^ particularly for early-stage COVID-19.^749^ In addition, intranasal delivery of imiquimod restrains viral load, weight loss, pulmonary inflammation and neutrophil infiltration, and increases antibody titers in the airway against IAV infection in mice.^750^ R848, a TLR7/8 agonist, suppresses SARS-CoV-2 replication in murine lungs and tracheas but not nasal turbinates.^751^ It promotes immune cell maturation, T cell proliferation and myelopoiesis, reducing weight loss and viral loads in IAV- or RSV-infected mice.^752,753^ R848 also improves bacterial clearance and survival in septic mice, promoting peritoneal phagocytosis in a type I IFN-independent manner.^472,754^ Other TLR7/8 agonists, 3M-052 and 3M-011, protect murine and ferrets from IAV challenge.^755,756^ TLR7/8 agonist 9.2 s-RNA stimulates the production of C-X-C motif chemokine ligand 10 during IAV challenge but fails to alleviate severe signs of infection in mice.^757^

TLR7 inhibitors reduce capillary leakage, enhance macrophage phagocytosis, improve bacterial clearance and survival in mice with *Pseudomonas aeruginosa* infection.^285^ The TLR7/8 inhibitor enpatoran (M5049) underwent phase 1 and 2 trials for COVID-19, but failed to meet its primary efficacy goal of shortening recovery time.^758,759^ In regard to IAV infection, TLR7 inhibitor, immunoregulatory sequence 661, impairs pDC-derived type I IFN production but preserves epithelial-derived IFN production.^316^ Via blocking IFN response in pDCs and neutrophil chemoattractant release in monocytes, immunoregulatory sequence 661 alleviates influenza-related immunopathy and improves survival of influenza-infected mice.^316^ Furthermore, TLR7 genetic deletion delays the progression of secondary pneumococcal infection with unaffected survival rates in IAV-infected mice.^760^

### RIG-I

The RIG-I activating ODN ligand, 5’-triphosphate RNA, protects mice from IAV infection for up to 7 days, rescuing them from lethal influenza and preventing secondary streptococcal infection in a type I IFN-dependent manner.^757^ Prophylactic application of triphosphate RNA inhibits RSV replication in human, mouse and ferret airway cell lines in vitro*,* and in mouse and ferret lungs in vivo.^761^ In line with this, 5’ triphosphate RIG-I agonist M8 limits IAV replication, promotes viral clearance and improves survival of mice.^762^ Delivery of M8 through nanostructure virus-like particles blocks ongoing viral replication in previously infected cells.^763^ In addition, the short dsRNA serving as both IAV nucleoprotein siRNA and RIG-I agonist strongly suppresses IAV infection in cell culture and in mice.^764^

Pre- and post-treatment with synthetic triphosphate RNA improves survival in mice with lethal SARS-CoV-2 by up to 50% and 25%, respectively, reducing viral loads, lung inflammation, and increasing neutralizing antibodies, with better protection than recombinant type I IFN.^765^ Stem-loop RNA 14, a RIG-I agonist, shows therapeutic potential for viral control and antiviral protection against acute and chronic SARS-CoV-2 infection in mice.^766^ Likewise, mice pretreated with the RIG-I agonist stem-loop RNA 10 are characterized by promotion of M1 polarization of pulmonary macrophages, reduced weight loss and improved survival following IAV infection.^767^ Another RIG-I agonist, nCoV-L, also protects mice against lethal SARS-CoV-2 infection.^768^

Synthetic RNA designed to limit virus replication can disrupt RIG-I activity. A short chemically modified dsRNA-binding peptide nucleic acid can bind the viral RNA structure, inhibit influenza replication and interfere with RIG-I recognition, potentially controlling influenza immunopathology.^769^

## Future perspectives

Numerous NA sensors activate unique signaling pathways that drive critical illness progression, raising an important question: why are there so many sensors for a single biomolecule type? From our point of view, there are several explanations. First, while some NA sensors detect similar types of NA, they specialize in recognizing different NA structures, providing effective and specific threat detection with redundancy if one sensor fails. Second, NA sensors are distributed across cellular compartments, allowing continuous monitoring of danger signals from various sources. Third, overlapping sensors can synergize to amplify NA-mediated signals, enhancing immune responses and cell-type-specific responses in different cells targeted by different pathogens. Still, further research is needed to clarify the roles of these distinct NA sensors in critically ill patients.

It is not always black and white when it comes to the role of NA sensing in critical illness. Indeed, non-self-NA sensing is vital for infection control while chronic self-NA sensing promotes undesired host injury. The dual functions of NA detection pose a great challenge of maintaining balance between effective immune response against pathogens and limited tissue damage under inflammatory stress. Thus, restraining self-sensing without impairing pathogen recognition is significant for disease management, and the mechanistic understanding of how NA sensors distinguish self-NA from foreign NA is important. Under normal conditions, signaling from self-NA is mostly inhibited. Spatial separation, nucleosome tethering, modifications of self-NA and nuclease-mediated degradation are involved in self-sensing regulation.^244,770^ Besides, cell-free NA sensing is restrained by intracellular location of NA sensors and rapid degradation of extracellular NA.^132^ Nevertheless, the host immune system’s ability to detect non-self-NA with both sensitivity and specificity, despite the abundance of self-NA, is far more complex than our current knowledge encompasses. How to translate these basic discoveries into clinical applications to treat critical illness remains a challenge.

NA sensing serves as a bridge in the complex crosstalk between immune cells and tissue cells. NA sensing in non-immune cells initiates rapid responses including the secretion of cytokines and chemokines to alarm neighboring cells and recruit immune cells, the production of anti-pathogen proteins and cell death.^244^ Yet, whether this intercellular communication within and beyond the immune system via NA signaling fuels the development of critical illness is a pressing area of research. On the other hand, while cfDNA release is well-documented in critical illness, cfRNA and its clinical relevance are less explored, unlike in non-critical diseases like cancer. The complexity of cfRNA types, along with challenges in processing, extraction and detection, complicates clinical interpretation. Nonetheless, given the role of RNA sensing pathways in critical diseases and the insights gained from cfDNA research, cfRNAs hold promise as potential biomarkers for diagnosis, prognosis and management in critical illness.

Growing evidence indicates that critical illness syndromes like sepsis and ARDS are highly heterogeneous and would benefit from a personalized medicine approach.^771^ Given the significant variability in immune responses to injury between individuals, understanding NA sensing in critically ill patients could transform critical care through more customized treatment strategies. Profiling each patient’s NA sensor activity may help clarify immune status and guide precise interventions – dampening overactive sensors to reduce inflammation or activating certain sensors to strengthen immune defenses in high-risk cases. NA sensor profiles and cell-free NA could also serve as biomarkers to track disease progression, facilitating timely and targeted therapies. Leveraging NA sensing insights could improve patient outcomes and usher in a new era of precision medicine in intensive care.

## Conclusion

In summary, NAs as DAMPs and PAMPs derived from pathogen, cell stress, cell death and active release mechanisms, increase in different critical conditions and drive disease progression. Primarily through the activation of TLR3/7/8/9, cGAS-STING, AIM2, RIG-I and MDA5 signaling pathways, NAs initiate both host immunity and tissue damage, thereby promoting disease progression. Strategies such as the nuclease application, NA administration, genetic deletion and pharmacological agonists or antagonists have unveiled NA sensing as a significant contributor to critical conditions including ARDS, sepsis, trauma and ischemic diseases. A deeper comprehension of various NA variants and their downstream sensing pathways holds promise for the development of therapeutic interventions in critical illness management.
